# *Mammut pacificus* sp. nov., a newly recognized species of mastodon from the Pleistocene of western North America

**DOI:** 10.7717/peerj.6614

**Published:** 2019-03-27

**Authors:** Alton C. Dooley, Eric Scott, Jeremy Green, Kathleen B. Springer, Brett S. Dooley, Gregory James Smith

**Affiliations:** 1 Western Science Center, Hemet, CA, USA; 2 Cogstone Resource Management, Inc., Orange, CA, USA; 3 Department of Biology, California State University, San Bernardino, San Bernardino, CA, USA; 4 Kent State University at Tuscarawas, New Philadelphia, OH, USA; 5 U.S. Geological Survey, Denver, CO, USA; 6 Department of Earth and Environmental Sciences, Vanderbilt University, Nashville, TN, USA

**Keywords:** Proboscidea, Mastodon, Pleistocene, Mammutidae

## Abstract

A new species of mastodon from the Pleistocene of western North America, *Mammut pacificus* sp. nov. is herein recognized, with specimens identified throughout California and from two localities in southern Idaho. This new taxon differs from the contemporaneous *M. americanum* in having narrower teeth, most prominently in M3/m3, as well as six sacral vertebrae, femur with a proportionally greater mid-shaft diameter, and no mandibular tusks at any growth stage. All known Pleistocene *Mammut* remains from California are consistent with our diagnosis of *M. pacificus*, which indicates that *M. americanum* was not present in California.

## Introduction

The American mastodon (*Mammut americanum*) is one of the iconic megafaunal mammals of the North American Pleistocene, with a widespread distribution across nearly every US state, Canada, and Mexico. This ubiquitous distribution played a central role in the formation of an American identity and the founding of North American vertebrate paleontology (Semonin, 2000).

There have been surprisingly few detailed studies of North American mammutids since Osborn’s (1936) original and seminal work, likely owing to a mistaken perception that a common taxon that has been recognized for over 250 years must also be well understood. Most studies of American mastodons, post Osborn (1936), have either been occurrence reports or focused on environmental context. In recent decades, our knowledge of mastodon anatomy has made strides (Green, 2006; Fisher, 2008, 2009; Hodgson et al., 2008a) as has our understanding of their disappearance within the context of the late Pleistocene extinction of the North American megafauna (Dreimanis, 1968; Alford, 1974; Barnosky et al., 2004; Saunders et al., 2010; Zazula et al., 2014; Widga et al., 2017). While the genus *Mammut* was divided into several different species during the first half of the 20th century (summarized in Osborn (1936)), these taxa have not withstood detailed scrutiny, lacking discovery of additional specimens. Therefore, even though some taxa have never been formally synonymized with *M. americanum* (e.g., *M. oregense*; Hay, 1926), it has been generally accepted that by the Pleistocene there was only a single, highly variable mammutid species in North America, *Mammut americanum*.

*Mammut* fossils are particularly common in the eastern US, especially in Florida, New York, the Midwestern states of Missouri, Indiana, and Illinois, and the Great Lakes region, including Ohio and Michigan; specimens from these areas have dominated studies of mastodons (Warren, 1852; Saunders, 1977, 1996; King & Saunders, 1984; Green, 2006; Fisher, 2008, 2009; Hodgson et al., 2008a; Widga et al., 2017). Several specific sites have produced numerous specimens and have a particularly large impact on mastodon studies, including Boney Spring, Jones Spring, and Trolinger Spring, all in Missouri (Saunders, 1977, 1996). In contrast, mastodons known from the western US have received relatively little attention. While isolated remains, primarily of teeth, were discovered in California as early as the 1860s, based on specimen labels from the collections at the University of California—Berkeley Museum of Paleontology, prior to the 1990s the only significant concentration of western mastodon remains was from the asphalt deposits at Rancho La Brea in southern California, and even these were relatively rare and made up a minuscule percentage of the Rancho La Brea fauna (Stock & Harris, 1992). Based on these limited remains, Stock & Harris (1992) suggested that Rancho La Brea mastodons were smaller than their eastern counterparts, while Trayler & Dundas (2009) found that Rancho La Brea mastodon molars, specifically m3s, were narrower than those from Missouri, although both of these studies considered the Rancho La Brea samples to be referable to *M. americanum*.

The sample of western mastodons was bolstered in recent years by two major discoveries in late Pleistocene deposits. Beginning in the early 1990s, the construction of Diamond Valley Lake reservoir in western Riverside County, California, uncovered more than 700 mastodon bones representing more than 100 individuals (Springer et al., 2009, 2010). In 2010, construction of the Ziegler Reservoir in Snowmass Village, Colorado, resulted in the discovery of at least 35 individuals (Fisher et al., 2014). These two localities are by far the largest concentration of mastodon remains discovered in the western US and informed this study significantly.

The Diamond Valley Lake fossil collection is housed at the Western Science Center (WSC) in Hemet, CA, USA, and a portion of that collection forms the majority of the WSC public exhibits. One of the most prominent individual exhibit specimens is a partial mastodon skeleton (catalog number WSC 18743, popularly known as “Max”), which was reported by Springer et al. (2009, 2010) as the largest mastodon known from the western US. In 2014, while preparing updated information panels for the exhibits, it was recognized that Max had small, narrow third molars despite the large size of other skeletal elements. An attempt to understand that observation ultimately led to the research project described herein. As we accumulated data and began to observe consistent (if partially overlapping) quantifiable differences in character distributions between eastern and western mastodons, we found that the simplest and most robust explanation for these differences is that we were observing two morphologically distinct lineages, justifying separate species designations for the two populations (see De Queiroz, 2007 for a discussion of morphospecies concepts and their relationship to lineage splits).

## Methods

Mammutid teeth in the below noted repositories were measured using digital calipers for maximum crown length and width at each loph or lophid. Additional measurements were obtained from published sources, including Bravo-Cuevas, Morales-García & Cabral-Perdoma (2015), Gidley (1926), Green (2006), Green & Hulbert (2005), Harington (1977), Hay (1923, 1926), Hibbard (1952), Hunt & Richards (1992), Lucas & Morgan (1997), Lundelius (1972), Mead, Haynes & Huckell (1979), Miller (1987), Osborn (1936), Pasenko (2011, 2012), Richards (1984), Richards, Whitehead & Cochran (1987), Schwimmer (1991), Silverstein (2017), Trayler & Dundas (2009), and Woodman & Branstrator (2008). Measurements for specimens from Trolinger Spring and Boney Spring were calculated from graphs published in Saunders (1977) using GraphClick (http://www.arizona-software.ch/graphclick/). Dental measurements are included in Tables S1 and S2. Measurements of postcranial elements follow Hodgson et al. (2008a). Dental terminology and wear descriptions follow Saunders (1977), and age estimations are given in African Equivalent Years (AEY) using tooth wear groupings (LG) from Laws (1966). Means and standard deviations were calculated using Apple Numbers, and Shapiro–Wilk tests and *T*-tests were conducted using Wizard v. 1.9.18. Comparisons to the Buesching mastodon, the Fowler Center mastodon, and the Routsong mastodon were made using 3D models of these specimens available at the University of Michigan Museum of Paleontology Online Repository of Fossils (https://umorf.ummp.lsa.umich.edu/wp/).

For inclusion in this study, all teeth needed to include at least state- or province-level locality information, and reasonable confidence as to geological age. Where radiometric dates were not available, specimens were grouped by North American Land Mammal Age (NALMA) following Bell et al. (2004). Only specimens that could be reliably assigned to either the Irvingtonian or Rancholabrean NALMA were included; some premolars from Florida were assigned as either Rancholabrean or Irvingtonian by the repository. When reliable measurements for the left and right tooth, in the same position from the same individual (in the case of in situ specimens), were obtained, either tooth was considered to be satisfactory for the statistical analyses. In such cases, one of the two teeth were chosen at random for measurement.

The electronic version of this article in portable document format will represent a published work according to the International Commission on Zoological Nomenclature (ICZN), and hence the new names contained in the electronic version are effectively published under that Code from the electronic edition alone. This published work and the nomenclatural acts it contains have been registered in ZooBank, the online registration system for the ICZN. The ZooBank Life Science Identifiers (LSIDs) can be resolved and the associated information viewed through any standard web browser by appending the LSID to the prefix http://zoobank.org/. The LSID for this publication is: urn:lsid:zoobank.org:pub:21ED43A5-6102-4D44-80C1-7D7A3D4651EA. The online version of this work is archived and available from the following digital repositories: PeerJ, PubMed Central, and CLOCKSS.

### Radiocarbon dating methods

At Diamond Valley Lake, we used radiocarbon (^14^C) dating of charcoal (charred vascular plants), wood, and small terrestrial gastropod shells of the Succineidae family to establish age control for deposits containing the vertebrate fossils. Charcoal and wood samples were treated using the standard acid-base-acid procedure (Trumbore, 2000). Clean, dry shells were broken and examined under a dissecting microscope to ensure that the interior whorls were free of secondary carbonate and detritus. Fossil shells that were free of detritus were etched with dilute HCl to remove 30–50% of the total mass prior to hydrolysis (Pigati, 2015).

Pretreated organic samples were combusted online in the presence of excess high-purity oxygen, whereas shell carbonate was converted to CO_2_ using American Chemical Society reagent grade 85% H_3_PO_4_ under vacuum at 80 °C until the reaction was visibly complete (∼1 h). For all samples, water and other contaminant gases (including SOx, NOx, and halide species) were removed using a combination of cryogenic separation and high-temperature fine wire copper and silver traps. The resulting purified CO_2_ gas was measured manometrically, converted to graphite using an iron catalyst and the standard hydrogen reduction process, and submitted for AMS ^14^C analysis. All ^14^C ages were calibrated using the IntCal13 dataset and CALIB 7.1html (Stuiver & Reimer, 1993; Reimer et al., 2013). Ages are presented in calibrated thousands of years before present (A.D. 1950), and uncertainties are given at the 95% (2σ) confidence level.

## Results

### Systematic paleontology

Order Proboscidea Illiger, 1811
Family Mammutidae Hay, 1922
Genus *Mammut* Blumenbach, 1799
*Mammut pacificus*, sp. nov.
urn:lsid:zoobank.org:act:BE79F9B4-1D49-415D-A250-6FB951CF81F6

**Holotype:** WSC 18743. Partial skeleton including largely complete cranium and mandible, with left and right M2/m2 and M3/m3, complete right tusk, distal 1/3 of left tusk, nearly complete sacrum and pelvis missing the anterior portion of the right ilium, distal end of the left femur, six vertebrae (fifth cervical, three posterior thoracic, two lumbar), and portions of at least eight ribs, LG XXII, 39 ± 2 AEY, and is interpreted as a male (based on body size, tusk size, and pelvic proportions), collected in 1995. The holotype is shown in Figs. 1–5, and measurements of key elements are included in Table 1. Key referred specimens are shown in Figs. 6–24 and listed in Table 2. Digital models of the holotype and key referred specimens are available on MorphoSource at https://www.morphosource.org/Detail/ProjectDetail/Show/project_id/687. Graphical comparisons of *M. pacificus* and *M. americanum* are shown in Figures 25–31.

**Figure 1:**
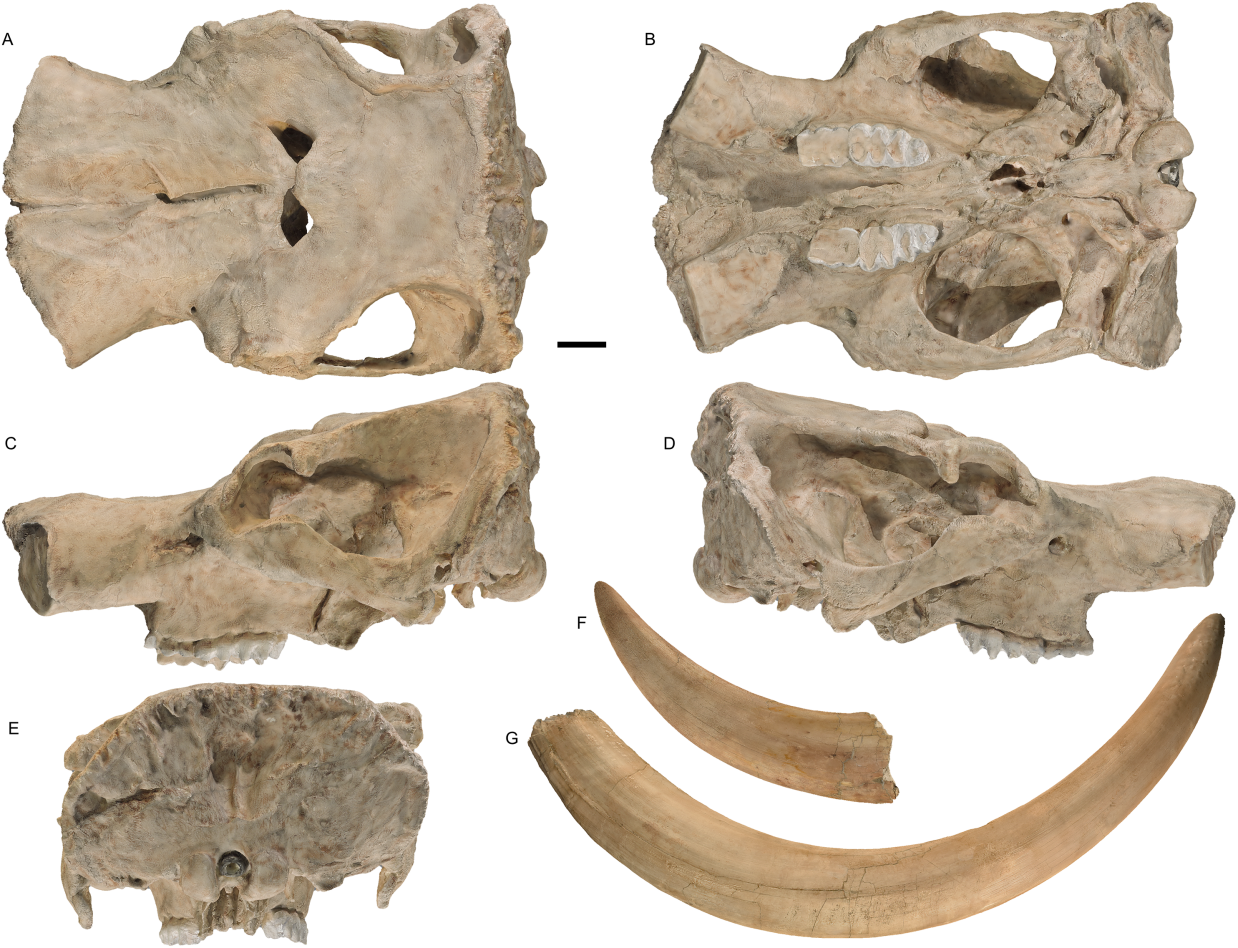
*Mammut pacificus* sp. nov., WSC 18743, holotype cranium and tusks. Cranium in: (A) dorsal, (B) ventral, (C) left lateral, (D) right lateral, (E) posterior, (F) distal end of left tusk (I1), lateral, and (G) right tusk (I1), lateral view. Teeth include left and right M2–M3. (A–E) are images of a resin cast of the holotype cranium on exhibit at the Western Science Center. All images are orthographic views of photogrammetric models. Scale = 10 cm.

**Figure 2:**
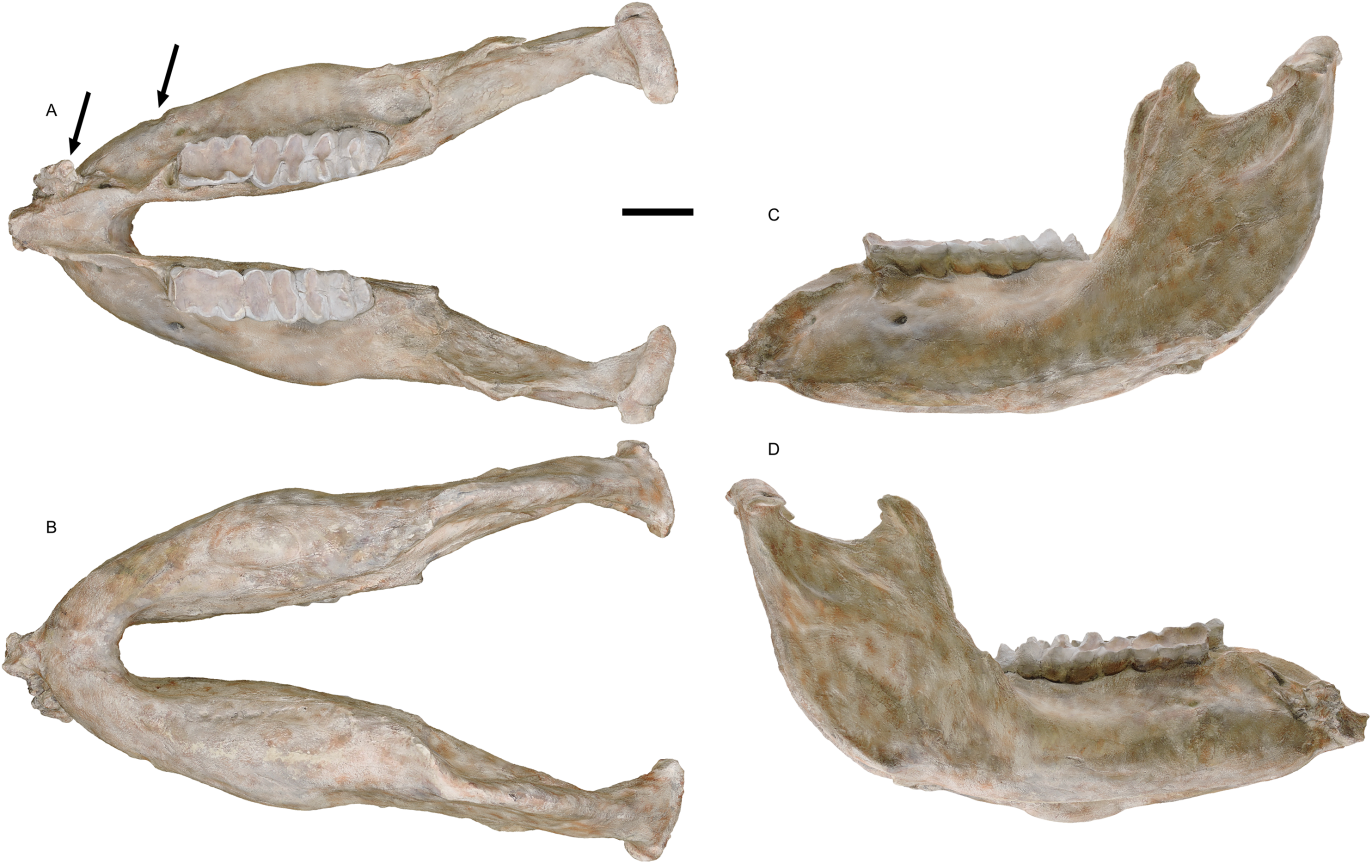
*Mammut pacificus* sp. nov., WSC 18743, holotype mandible. Mandible in: (A) dorsal, (B) ventral, (C) left lateral, and (D) right lateral. Teeth include left and right m2–m3. Images of a resin cast of the holotype mandible on exhibit at the Western Science Center. Arrows indicate pathologies mentioned in the text. All images are orthographic views of photogrammetric models. Scale = 10 cm.

**Figure 3:**
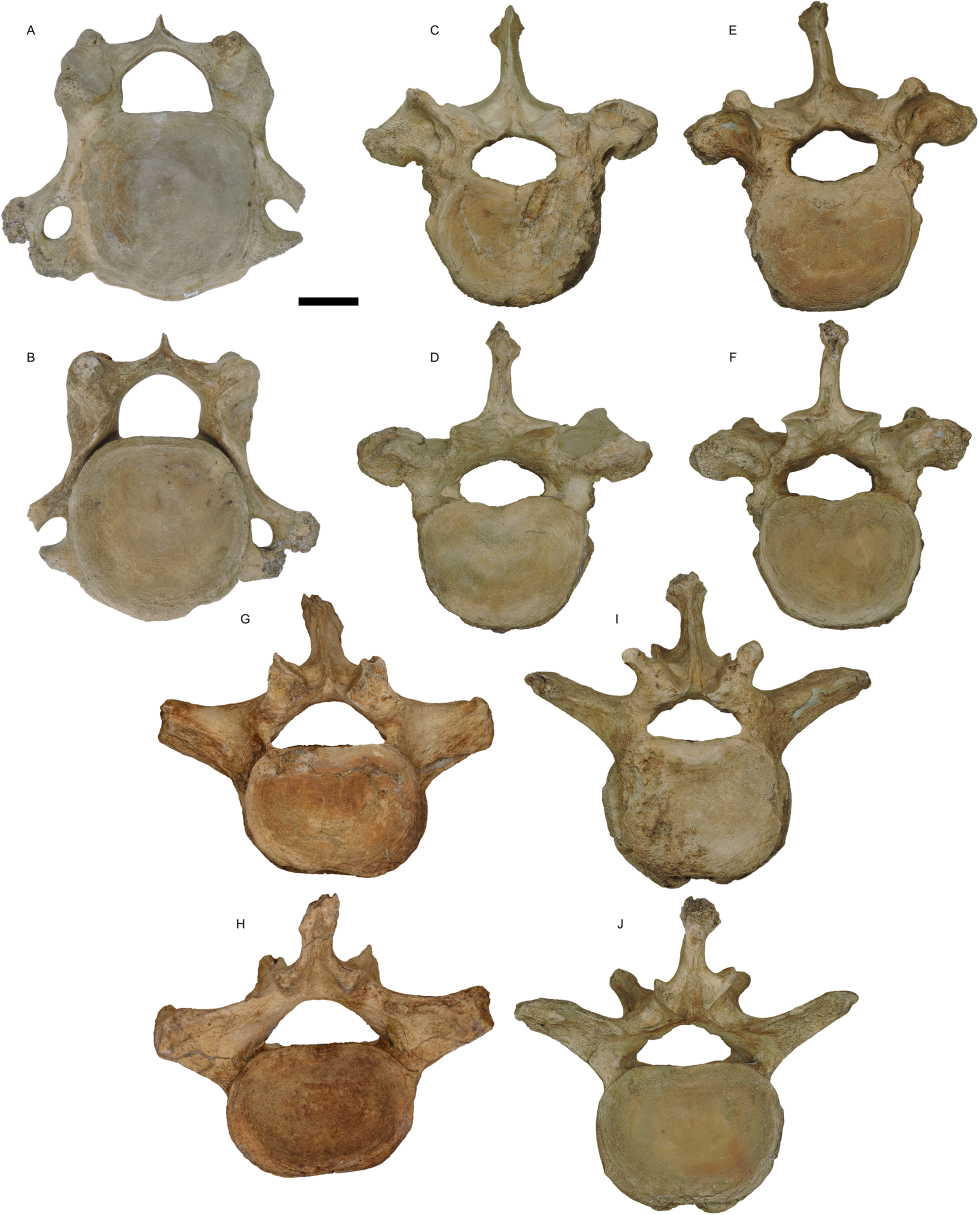
*Mammut pacificus* sp. nov., WSC 18743, holotype vertebrae. Fifth cervical vertebra in (A) anterior and (B) posterior views. Posterior thoracic vertebrae in (C) and (E) anterior and (D) and (F) posterior views. First lumbar vertebra in (G) anterior and (H) posterior views. Second or third lumbar in (I) anterior and (J) posterior views. (C–J) are orthographic views of photogrammetric models. Scale = five cm.

**Figure 4:**
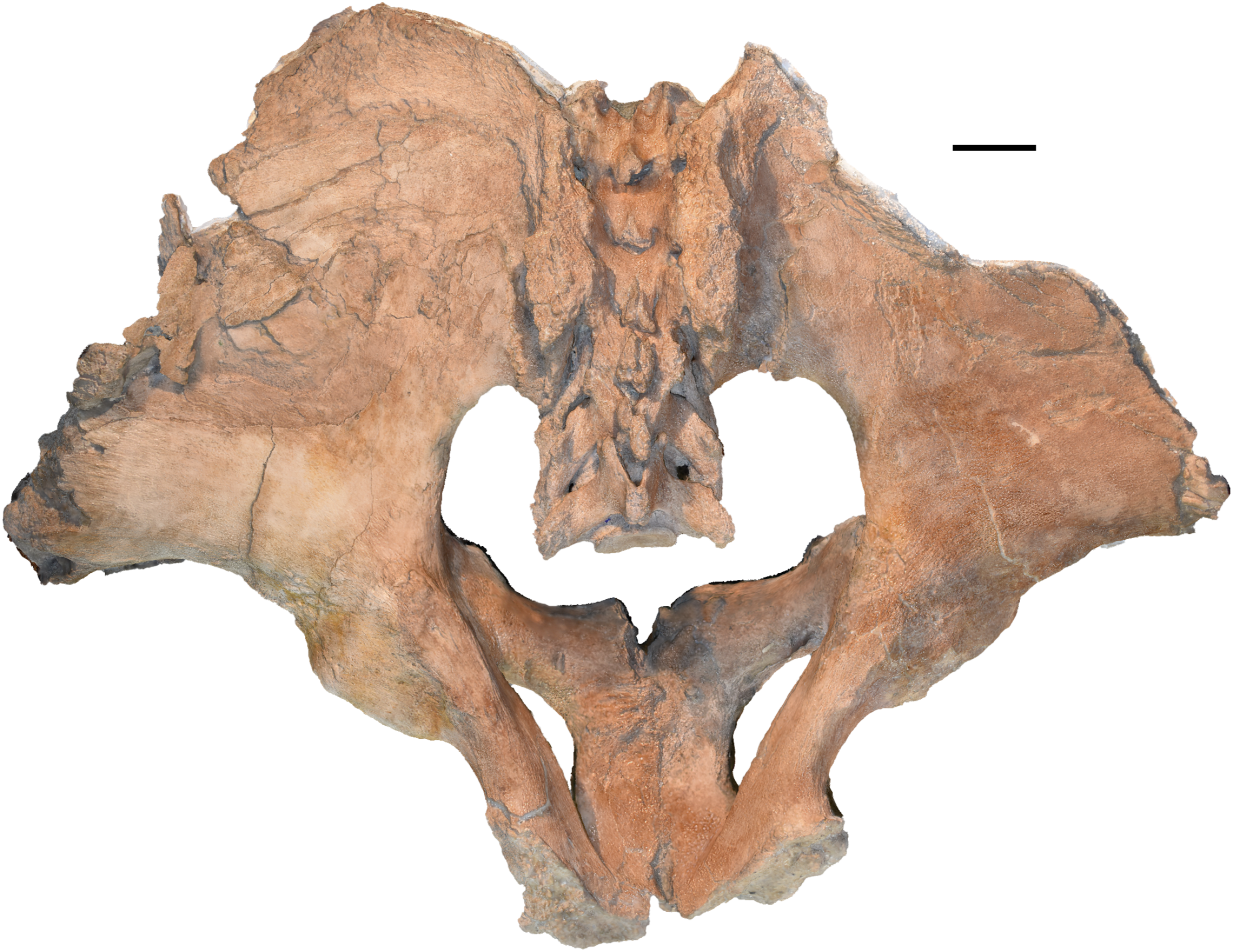
*Mammut pacificus* sp. nov., WSC 18743, holotype pelvis. Pelvis in dorsal view. Orthographic view of photogrammetric model. Scale = 10 cm.

**Figure 5:**
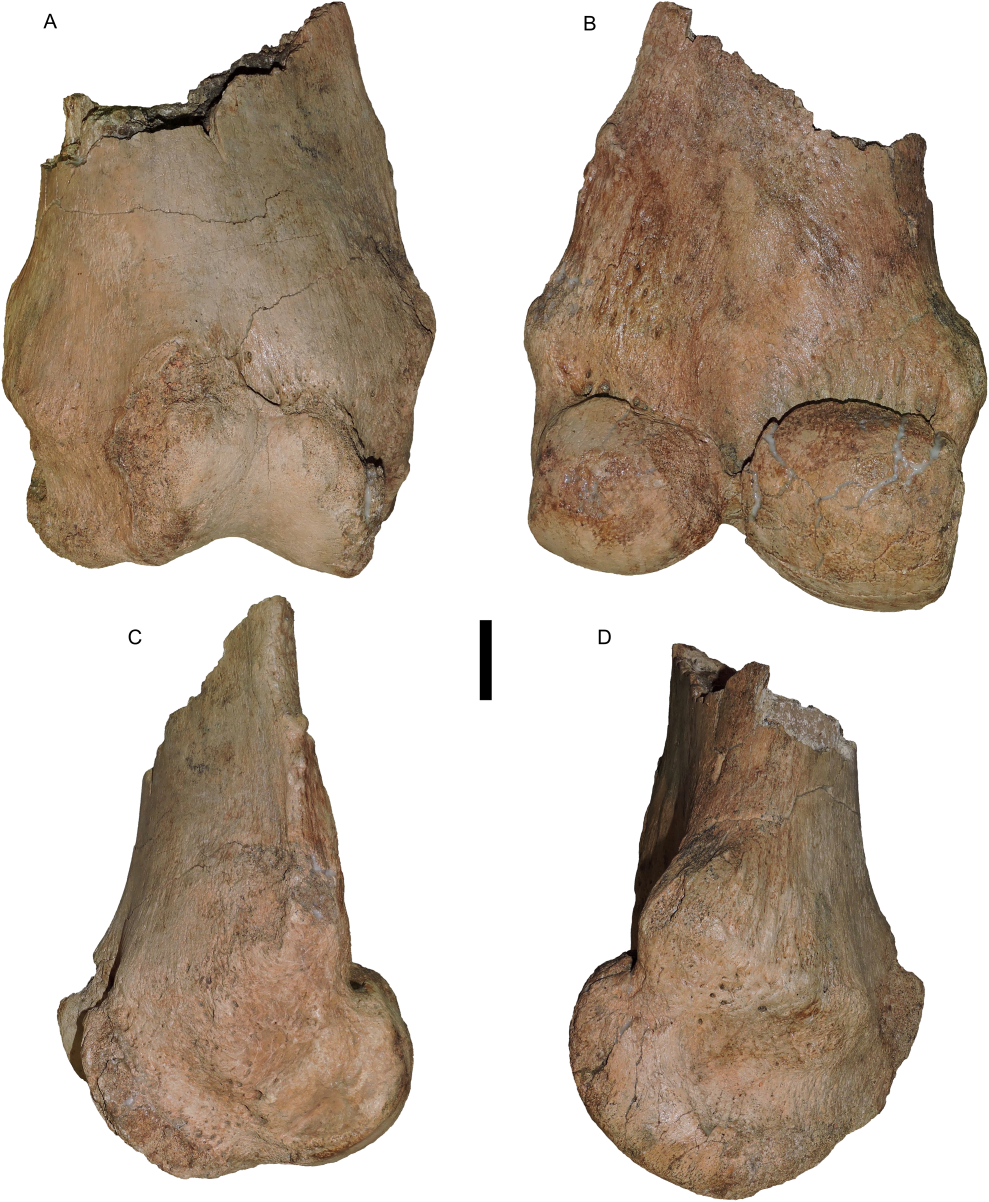
*Mammut pacificus* sp. nov., WSC 18743, holotype femur. Distal left femur in (A) anterior, (B) posterior, (C) lateral, and (D) medial views. Scale = five cm.

**Figure 6:**
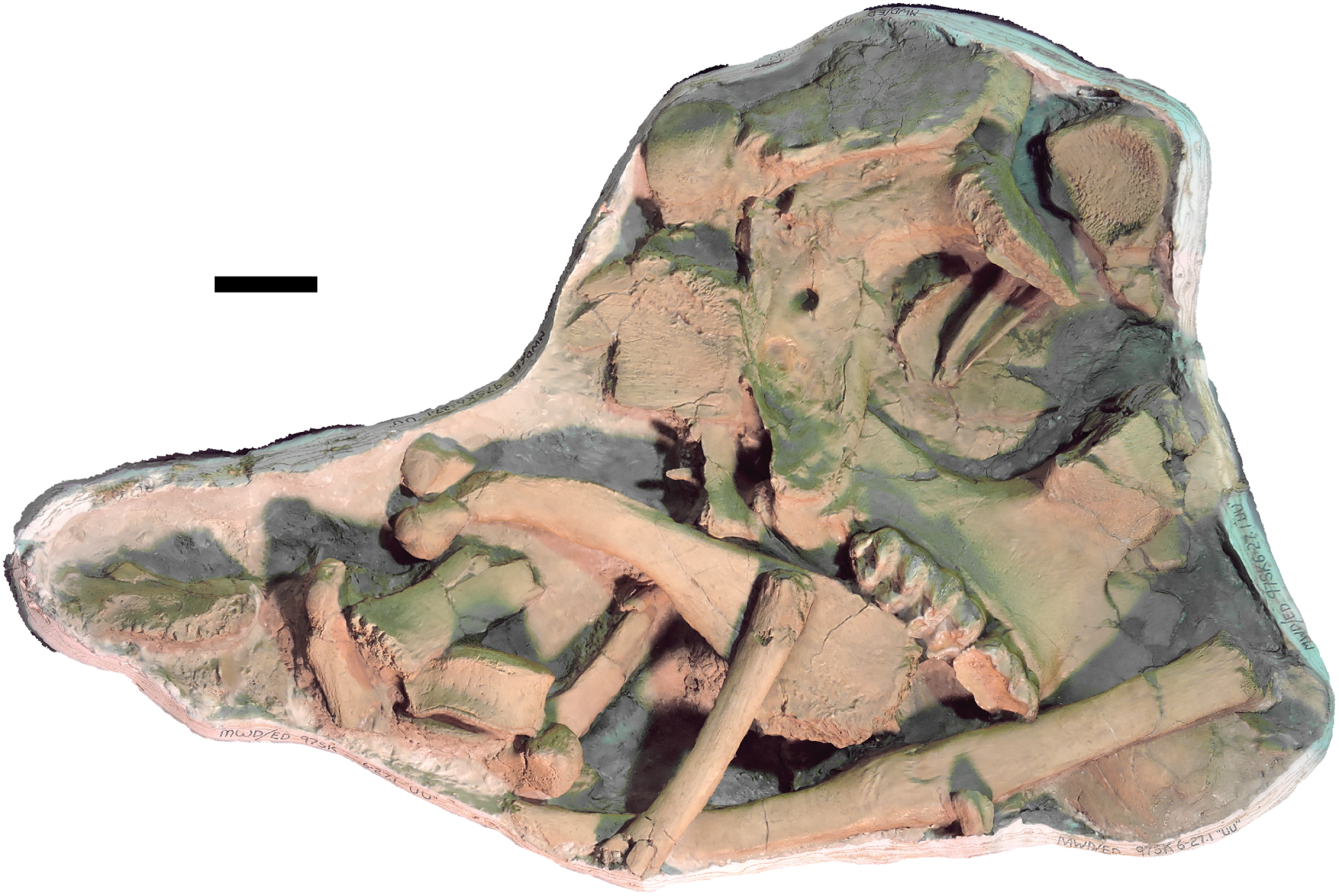
*Mammut pacificus* sp. nov., WSC 9622, referred cranium. Partial cranium in ventral view with right M2–M3, with associated ribs and vertebrae, in field jacket. Orthographic view of photogrammetric model. Scale = 10 cm.

**Figure 7:**
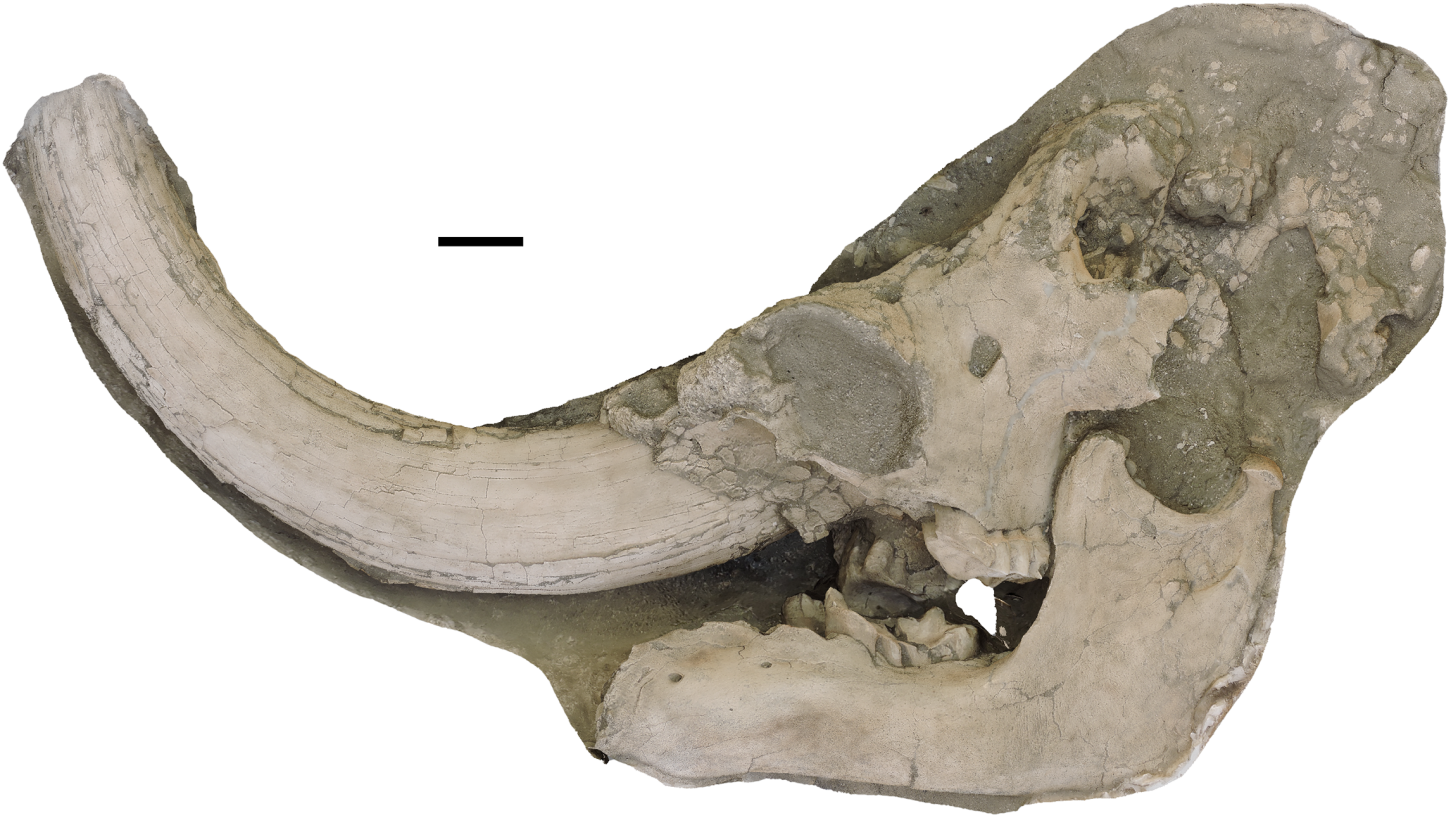
*Mammut pacificus* sp. nov., WSC 8817, referred skull. Partial cranium and mandible in left lateral view, with left and right M3 and m3, in field jacket. Orthographic view of photogrammetric model. Scale = 10 cm.

**Figure 8:**
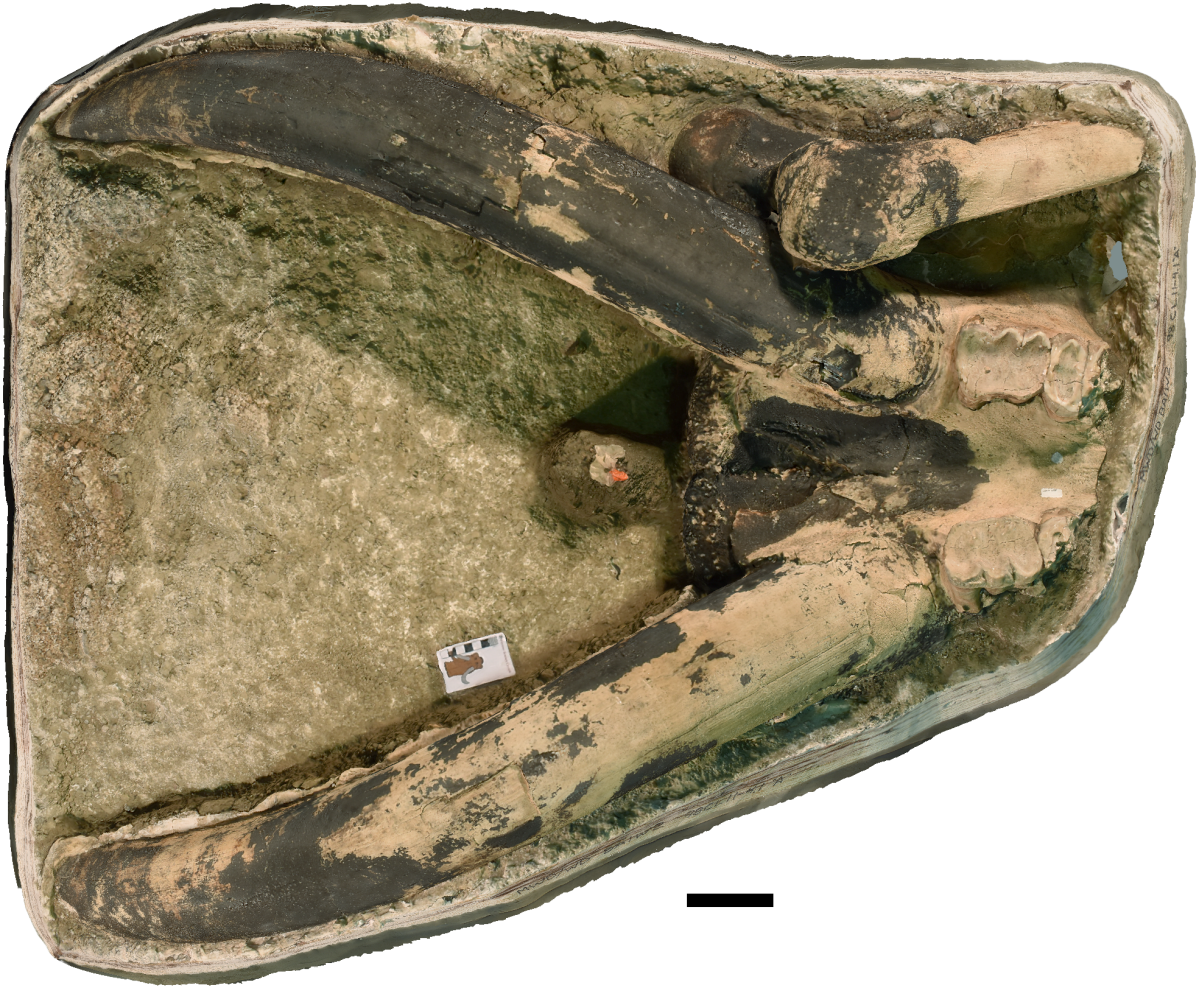
*Mammut pacificus* sp. nov., WSC 10844, referred cranium. Partial cranium in ventral view with left and right M2, and the anterior parts of left and right M3, with associated proximal left femur, in field jacket. Orthographic view of photogrammetric model. Scale = 10 cm.

**Figure 9:**
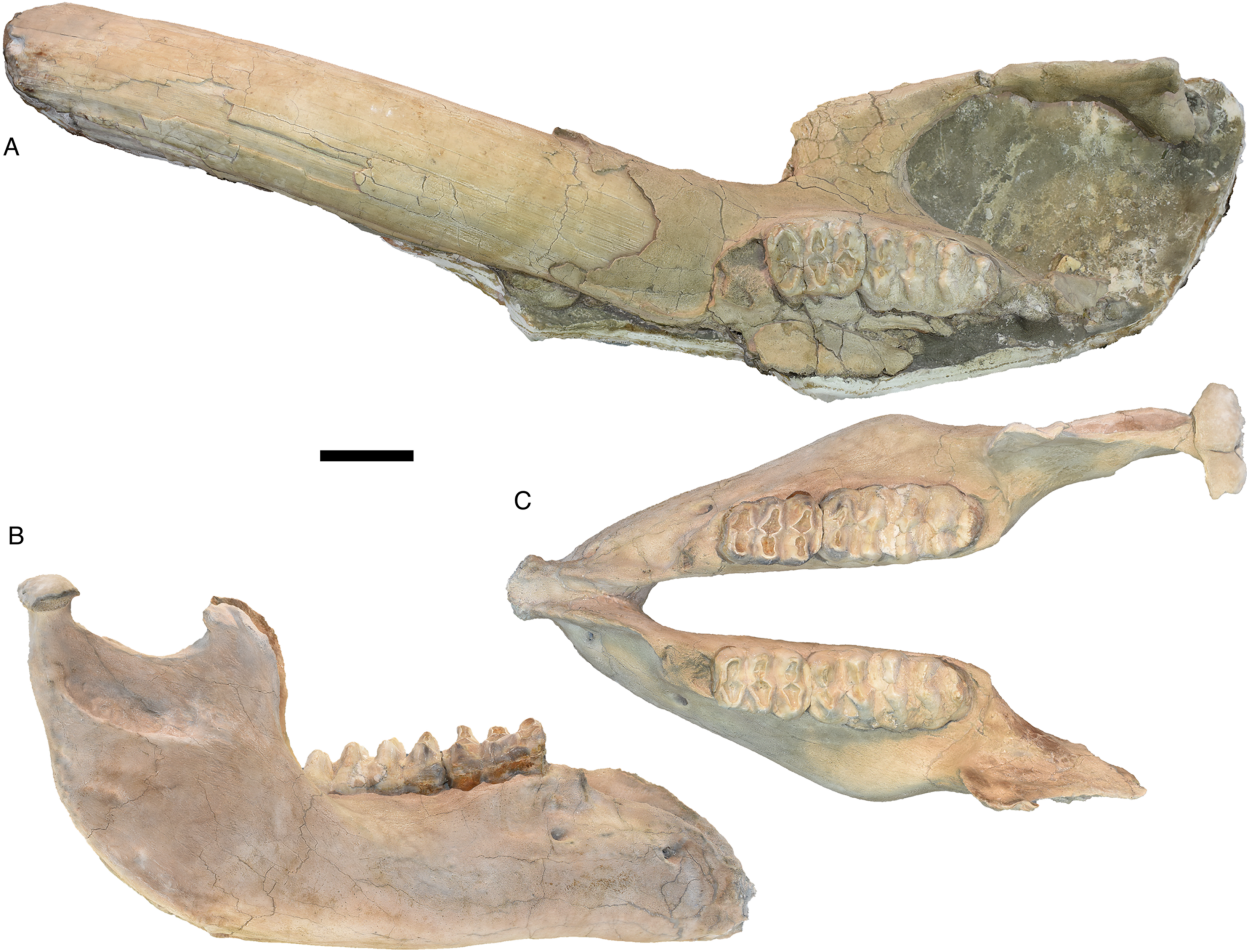
*Mammut pacificus* sp. nov., WSC 19730, referred skull. (A) Partial left cranium in ventral view, with left I1, M2, and M3, in field jacket, (B) mandible in right lateral view, and (C) mandible in dorsal view, with left and right M2–M3. Orthographic views of photogrammetric models. Scale = 10 cm.

**Figure 10:**
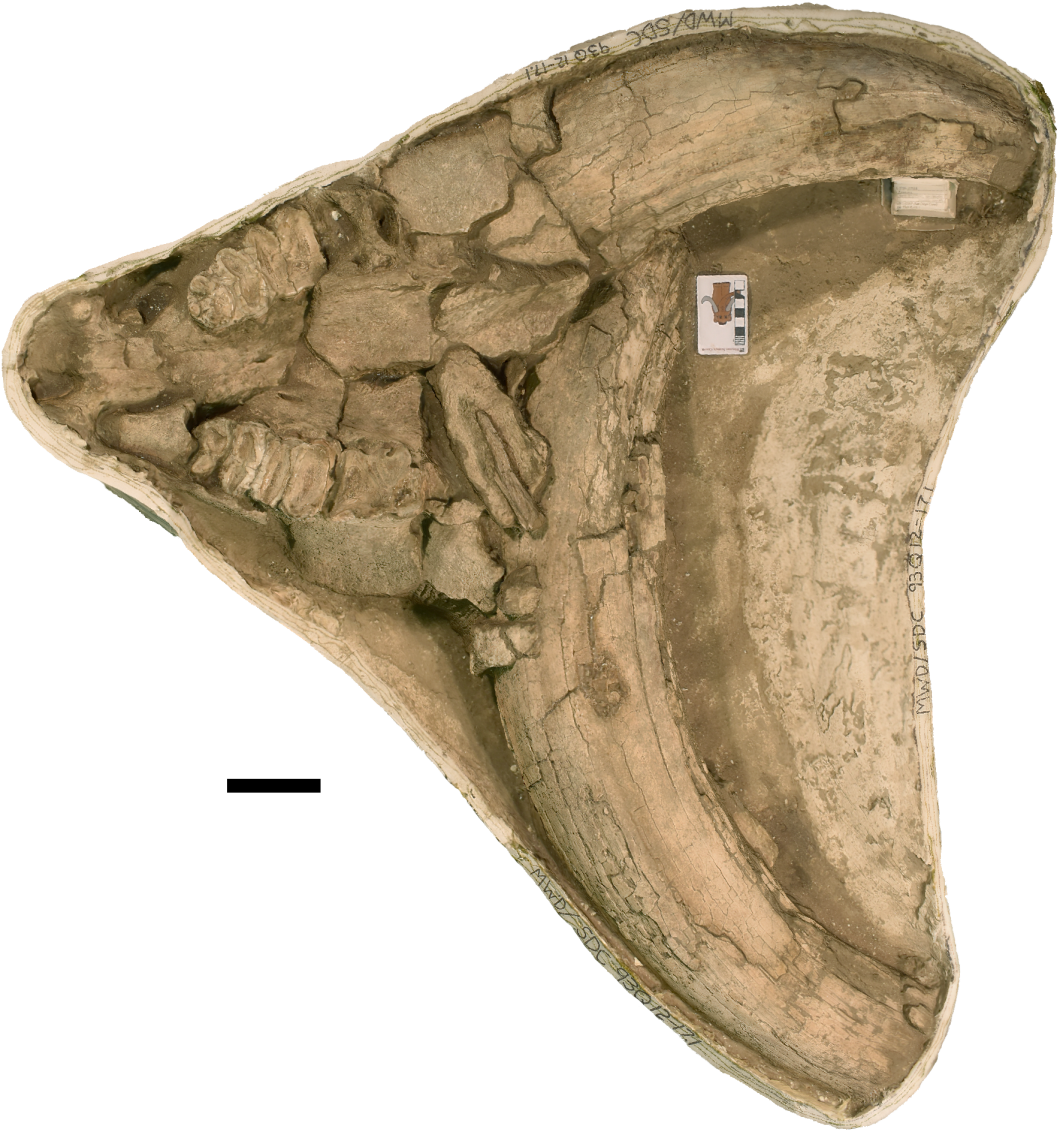
*Mammut pacificus* sp. nov., WSC 22587, referred cranium. Partial cranium in ventral view, with left M2 and left and right M3, in field jacket. Orthographic view of photogrammetric model. Scale = 10 cm.

**Figure 11:**
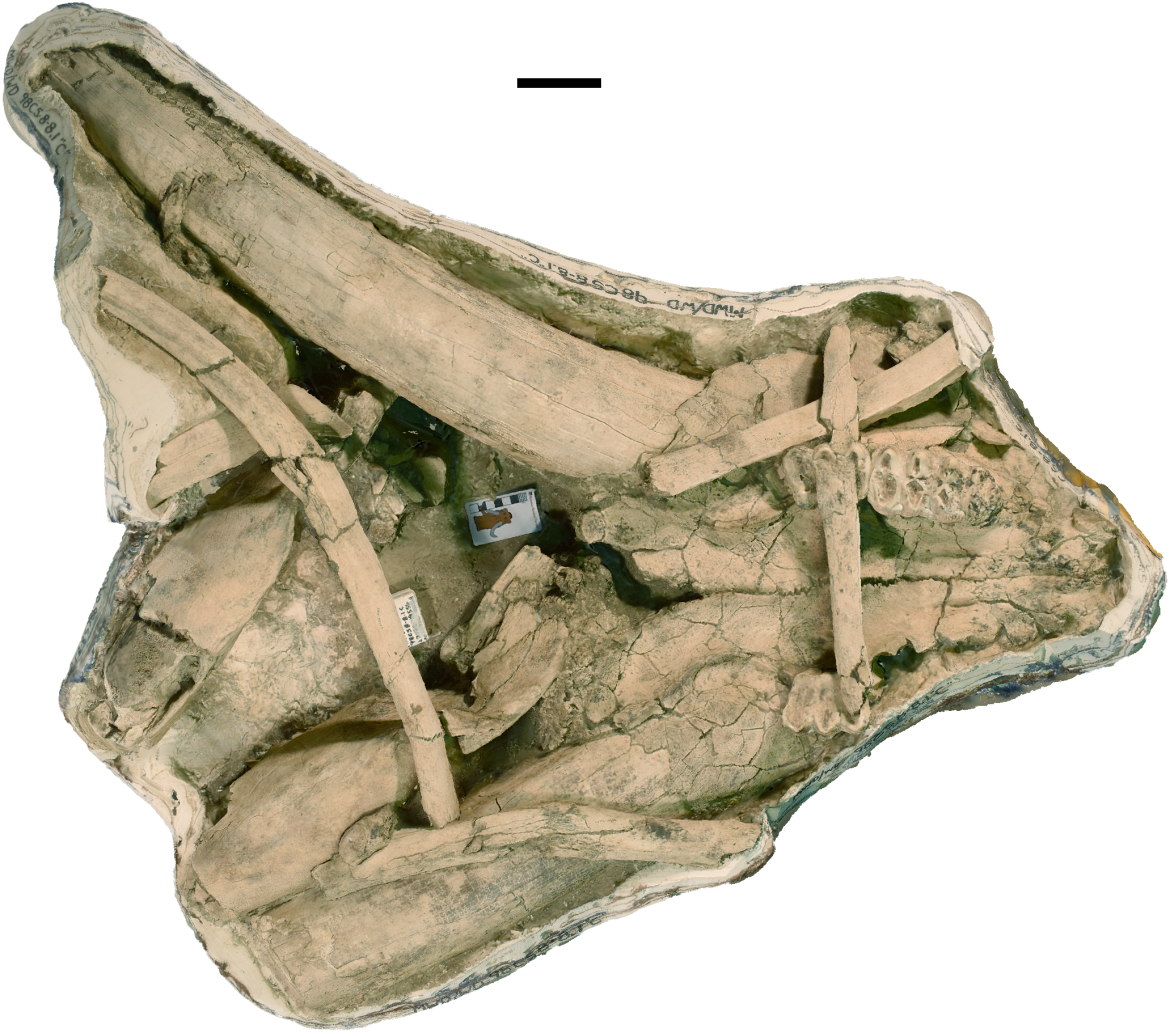
*Mammut pacificus* sp. nov., WSC 10829, referred skull. Partial cranium with left and right M2, and right M3, and proximal ends of both dentaries in ventral view with associated ribs, in field jacket. Orthographic view of photogrammetric model. Scale = 10 cm.

**Figure 12:**
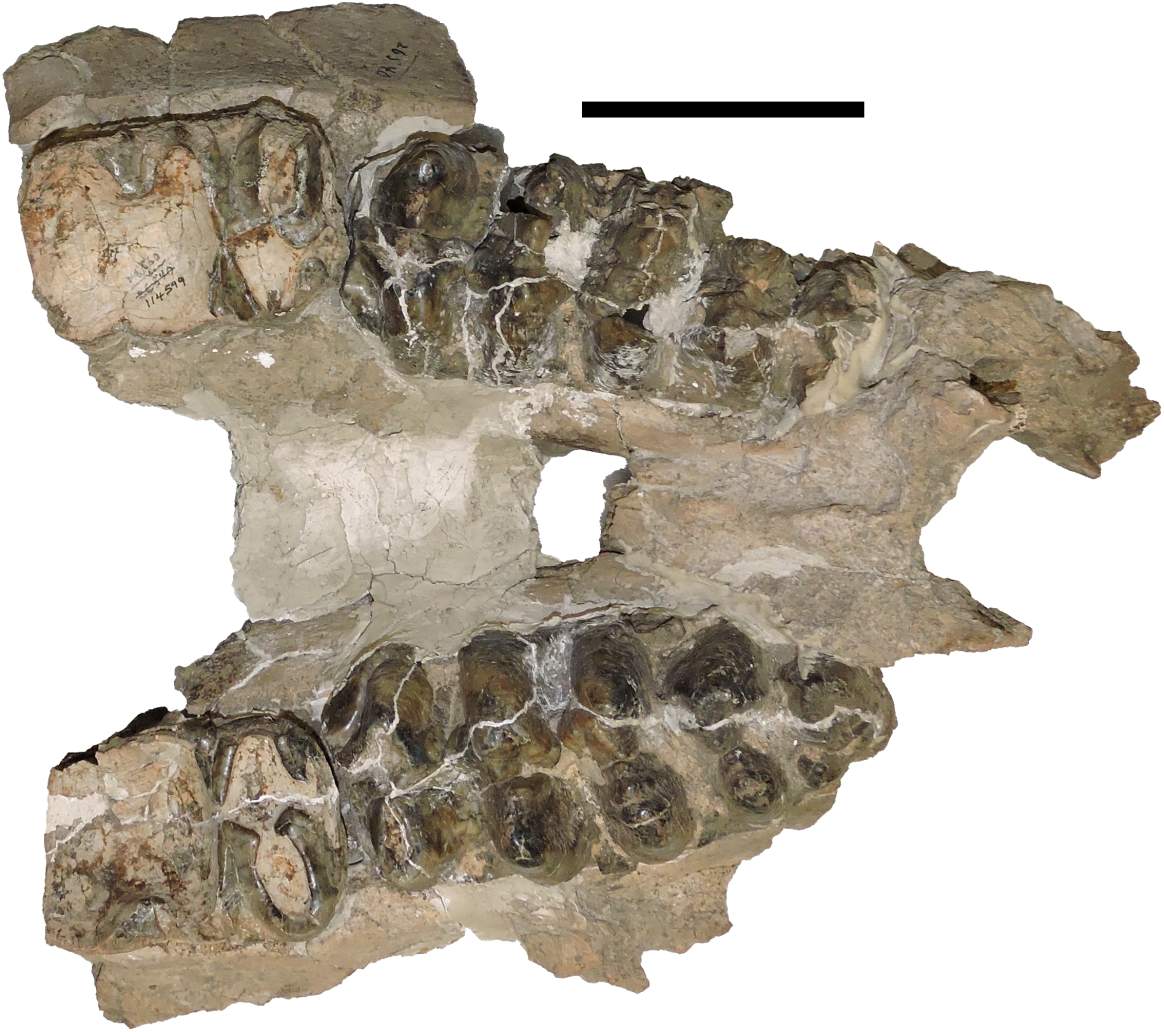
*Mammut pacificus* sp. nov., UCMP 114599, referred cranium. Partial cranium in ventral view, with left and right M2–M3. Scale = 10 cm.

**Figure 13:**
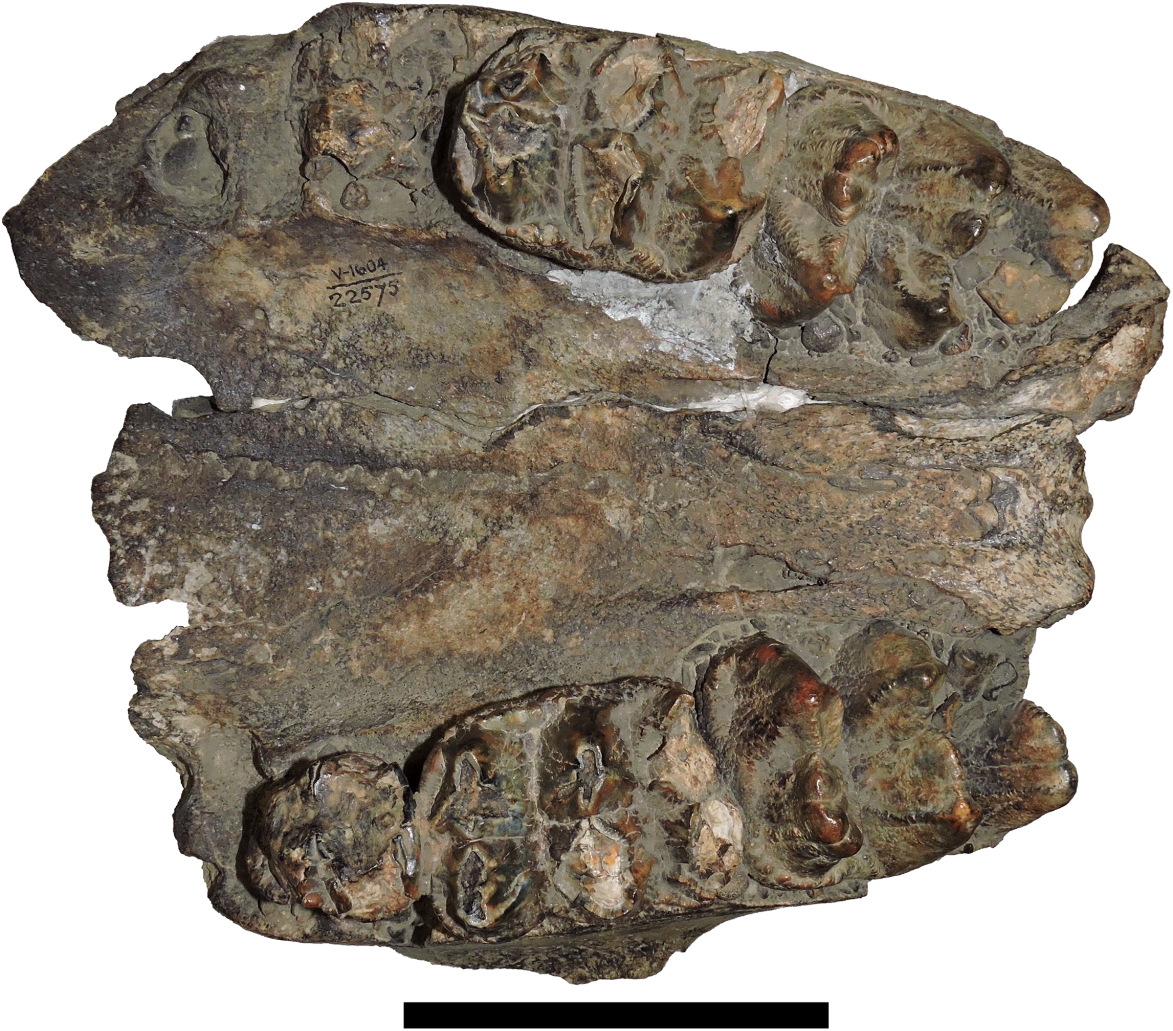
*Mammut pacificus* sp. nov., UCMP 22575, referred cranium. Partial cranium in ventral view, with left and right dP3–dP4, and M1. Scale = 10 cm.

**Figure 14:**
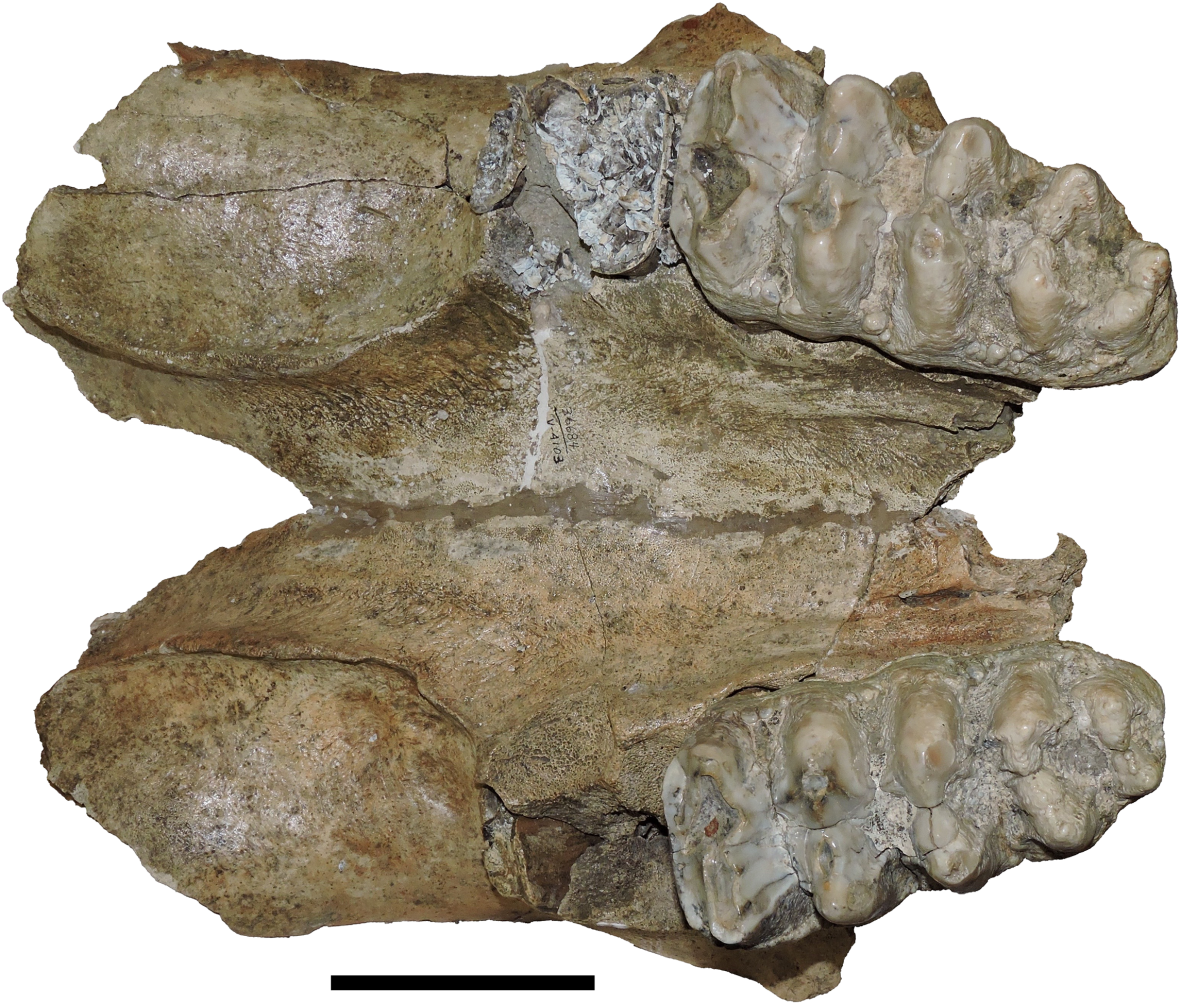
*Mammut pacificus* sp. nov., UCMP 36684, referred cranium. Partial cranium in ventral view, with left and right M3. Scale = 10 cm.

**Figure 15:**
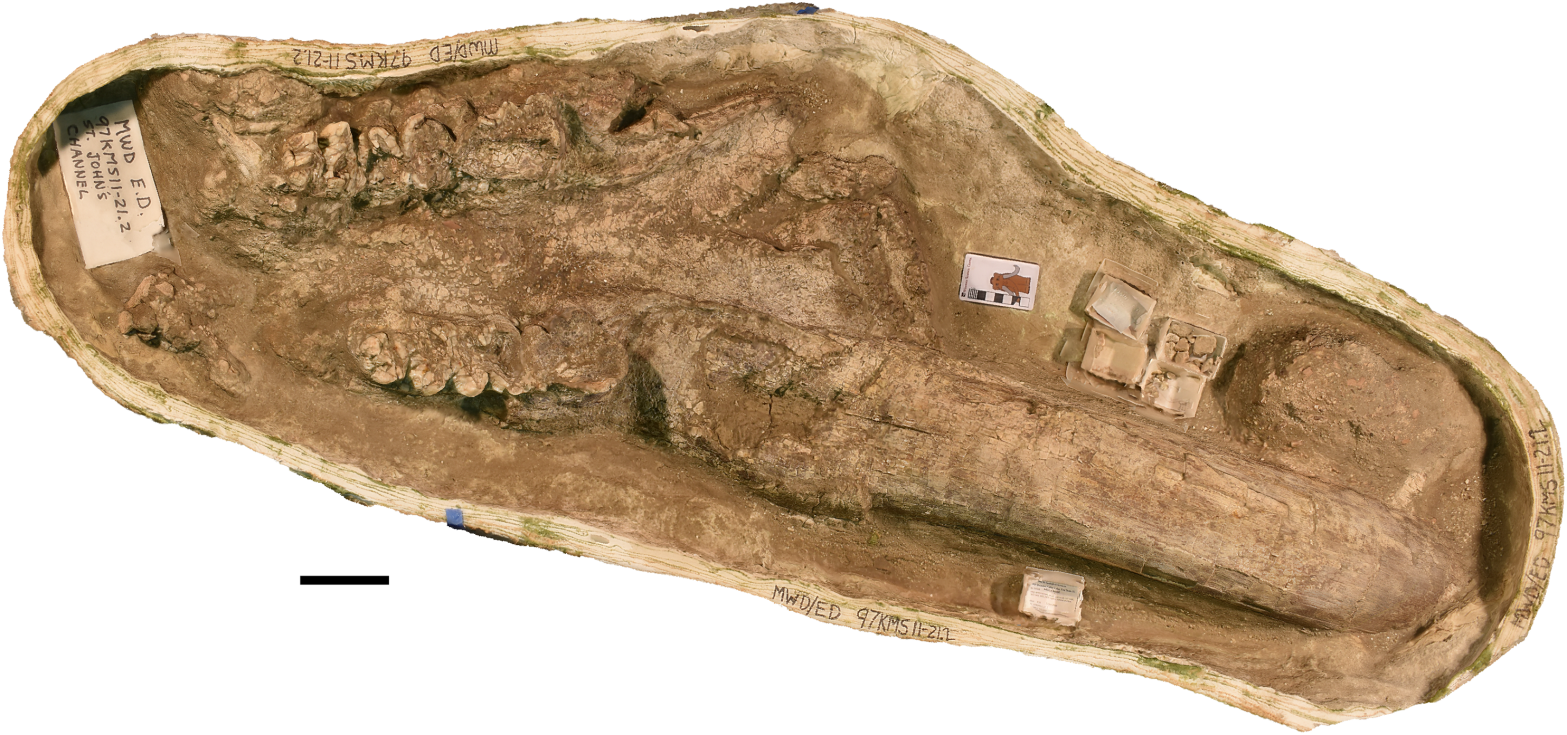
*Mammut pacificus* sp. nov., WSC 10819, referred cranium. Partial cranium in ventral view, with left I1 and left and right M2–M3, in field jacket. Orthographic view of photogrammetric model. Scale = 10 cm.

**Figure 16:**
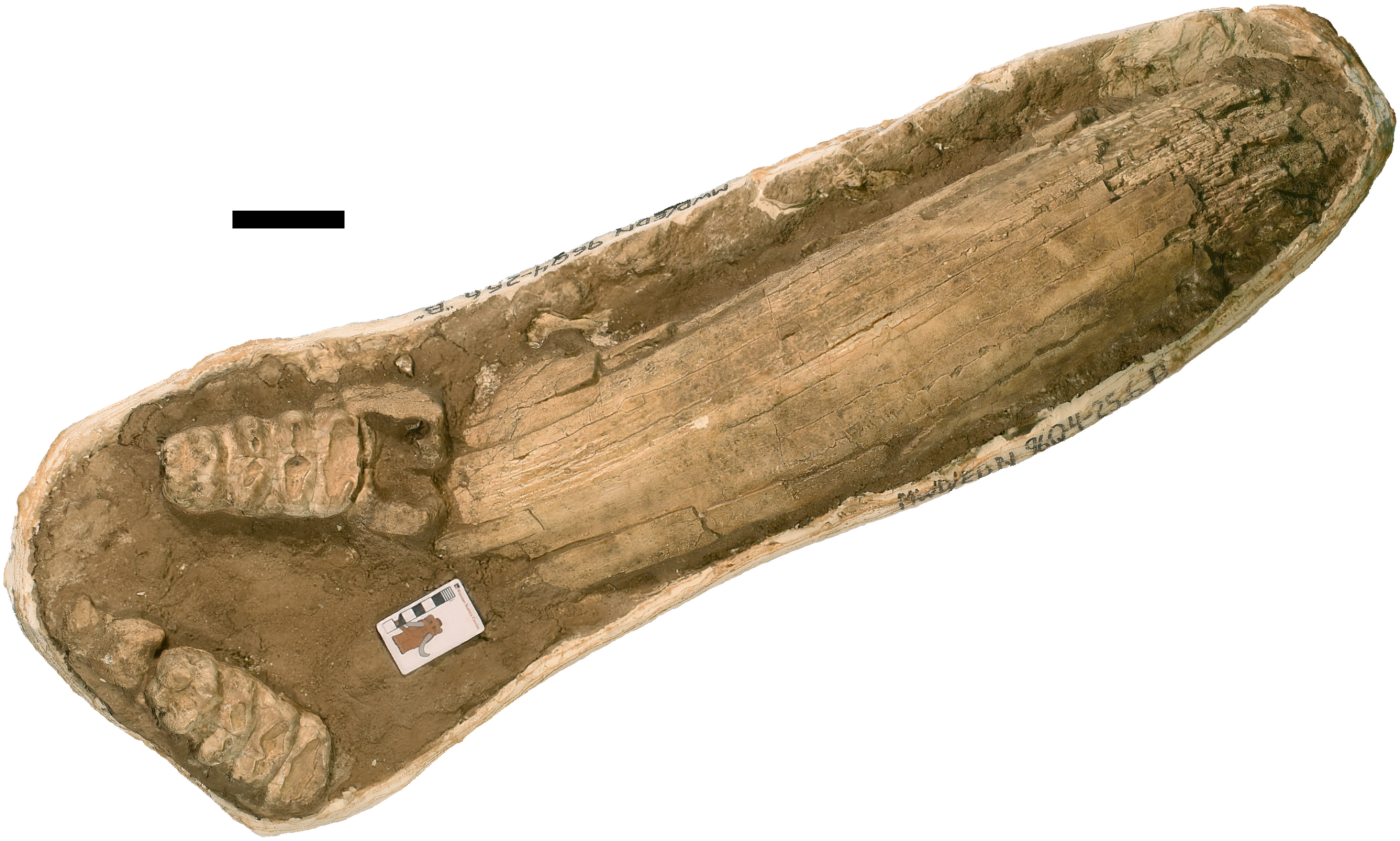
*Mammut pacificus* sp. nov., WSC 8904, referred cranium. Partial cranium in ventral view, with right I1 and left and right M3, in field jacket. Orthographic view of photogrammetric model. Scale = 10 cm.

**Figure 17:**
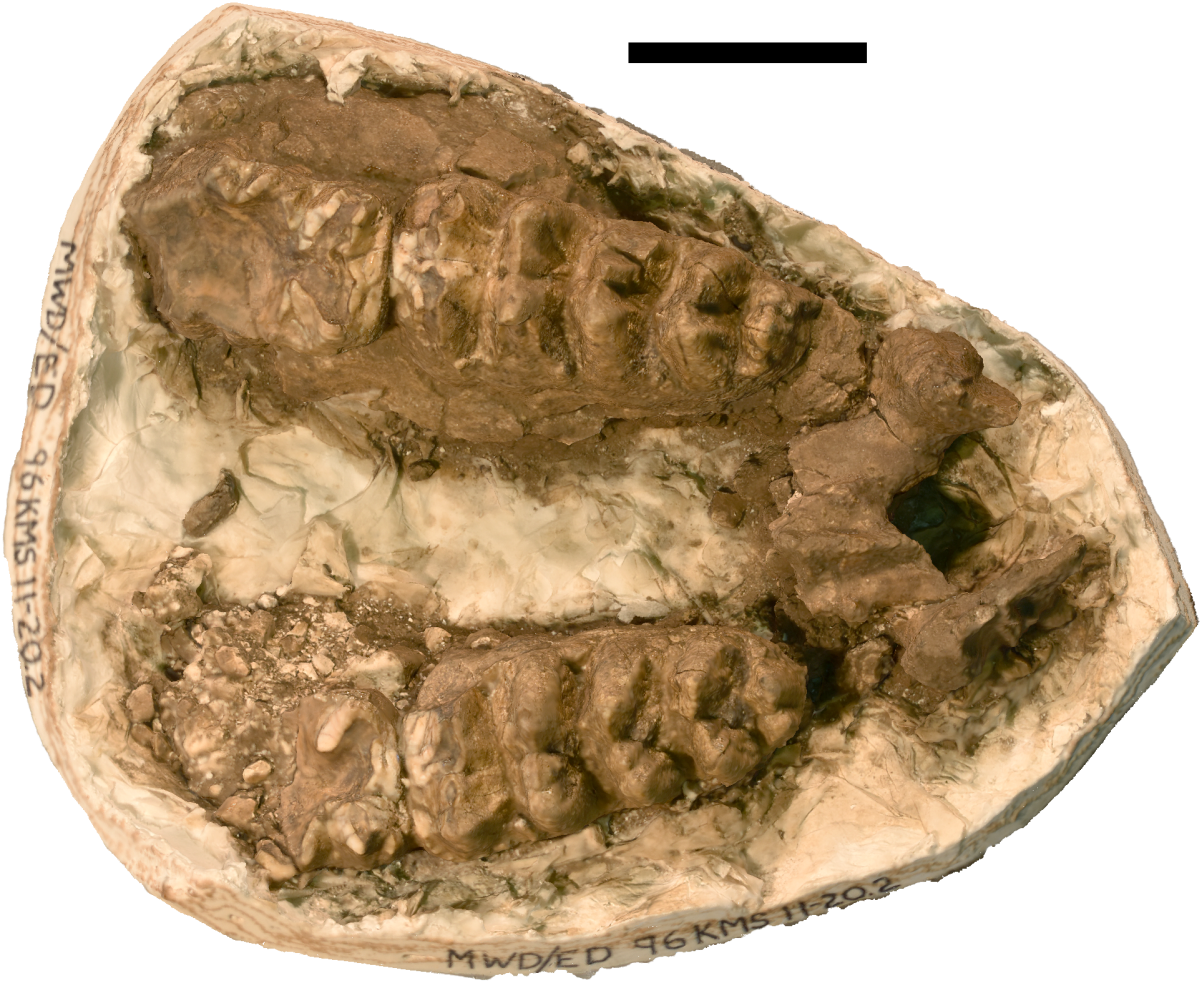
*Mammut pacificus* sp. nov., WSC 10646, referred cranium. Partial cranium in ventral view, with left and right partial M2 and complete M3, in field jacket. Orthographic view of photogrammetric model. Scale = 10 cm.

**Figure 18:**
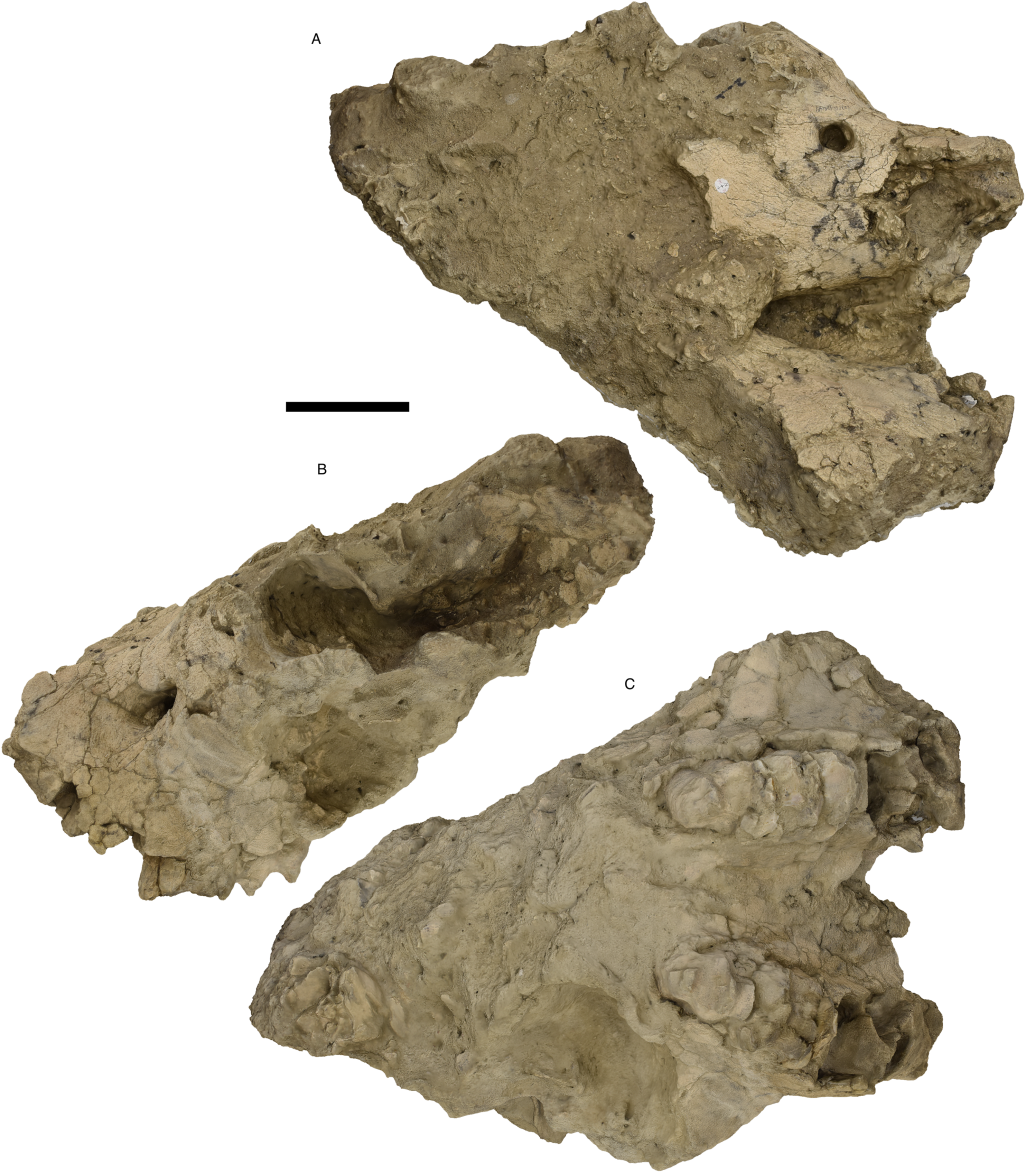
*Mammut pacificus* sp. nov., WSC 8917, referred cranium. Partial cranium with left and right M3 in (A) dorsal, (B) left lateral, and (C) ventral views. Orthographic views of photogrammetric model. Scale = 10 cm.

**Figure 19:**
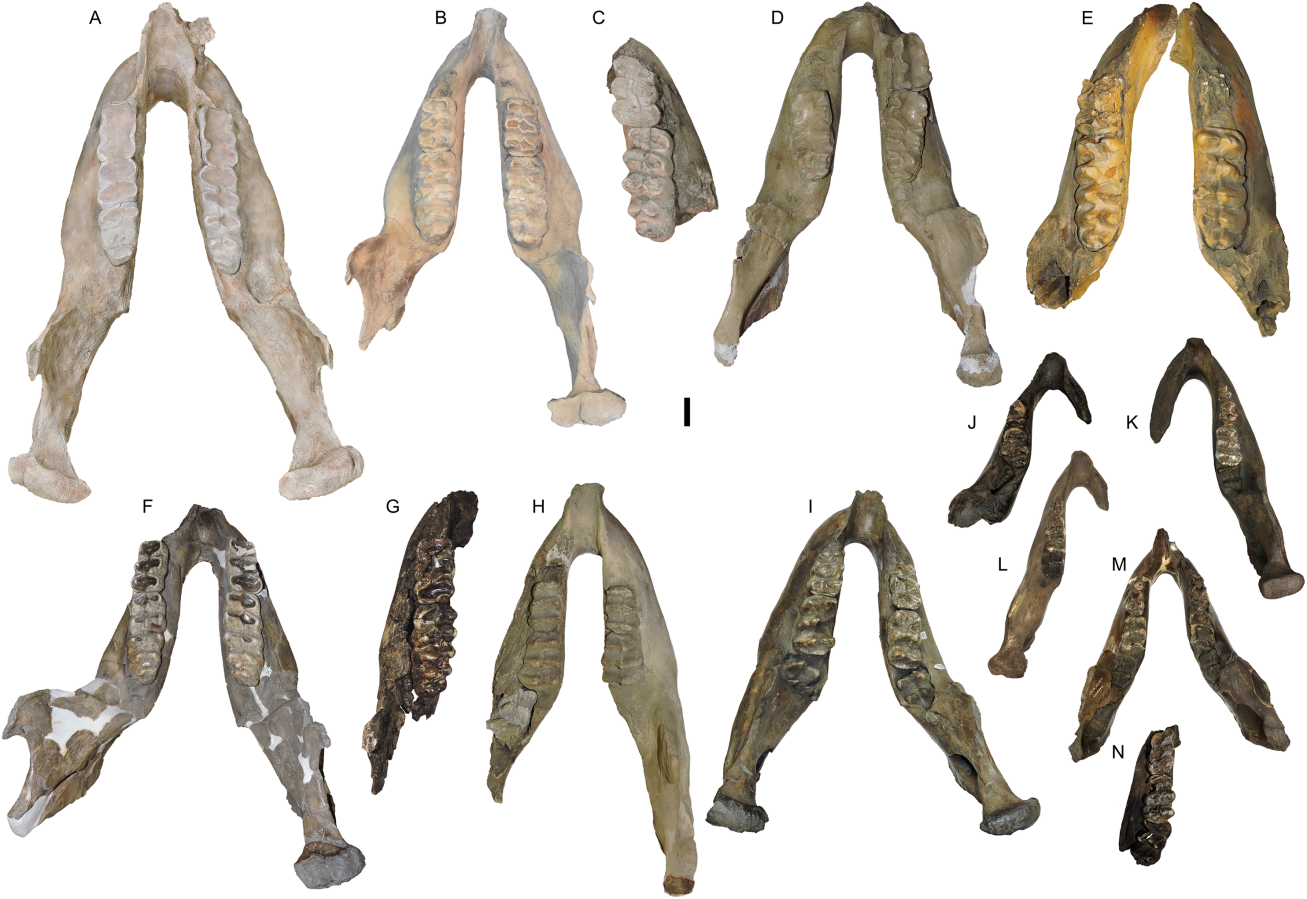
*Mammut pacificus* sp. nov., comparative dorsal views of mandibles. (A) WSC 18743, holotype, with left and right m2–m3, (B) WSC 19730, with left and right m2–m3, (C) LC 260, with right m2–m3, (D) LACM 152669, with right m2 and left and right m3, (E) UCMP 86140, with partial left m2 and left and right m3, (F) LACM-HC 77, with left and right m2–m3, (G) LACM-HC 87037, with left m2–m3, (H) LACM 128927, with left and right m1–m2 and partial unerupted left m3, (I) LACM-HC 38, with left and right m1–m2 and unerupted m3, (J) UCMP RSF0201, with left dp2–dp3, (K) LACM-HC 475, with right dp2–dp4 and unerupted m1, (L) LACM P23 23638, with left dp2–dp4 and unerupted m1, (M) LACM-HC 1631, with left and right dp2–dp4 and unerupted m1, and (N) LACM-HC 87093, with dp4–m1 and unerupted m2. Scale = five cm.

**Figure 20:**
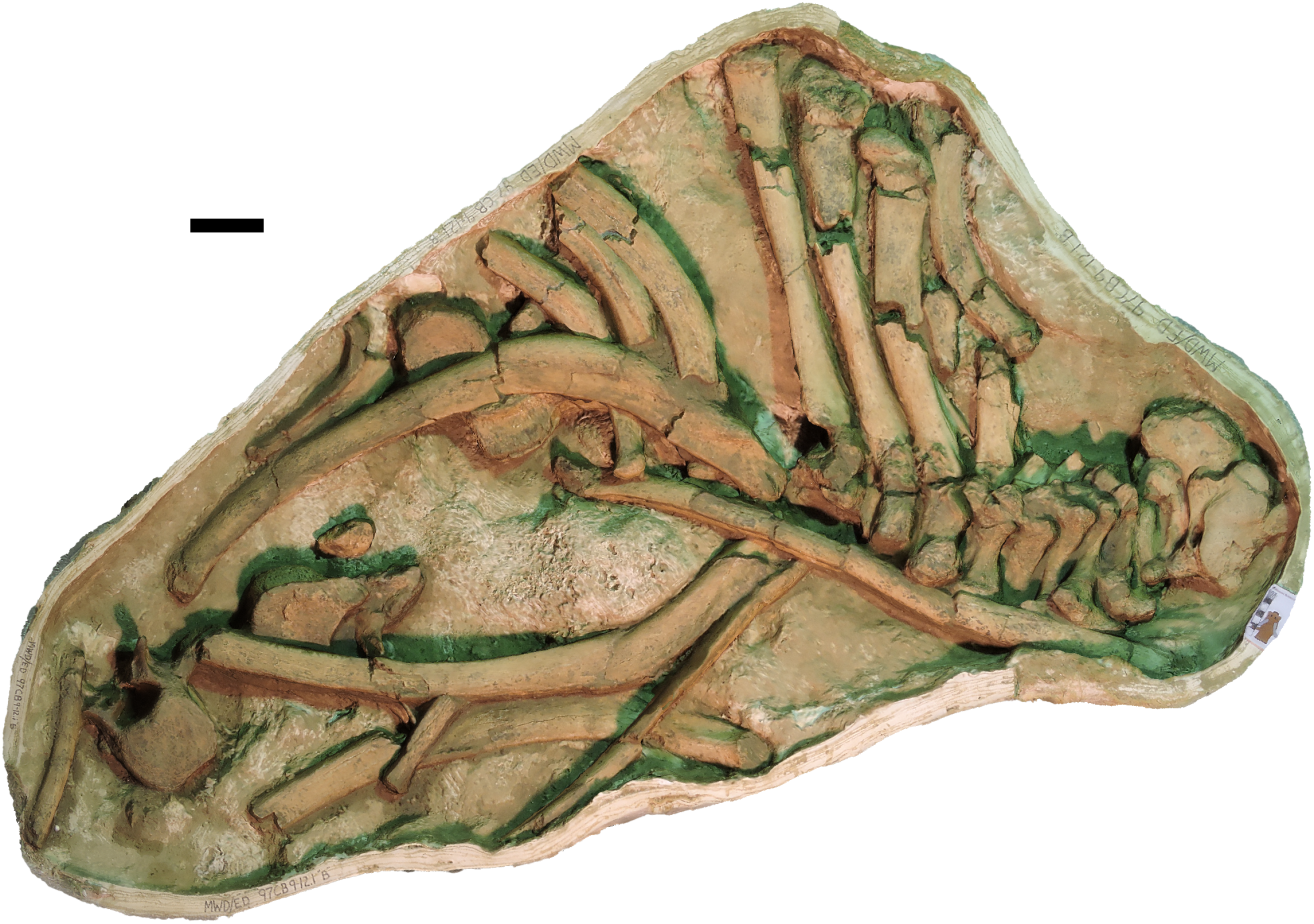
*Mammut pacificus* sp. nov., WSC 7561, referred partial articulated vertebral column. Partial skeleton in right lateral view, with the first 11 vertebrae in articulation, and at least four additional vertebrae, plus associated ribs, in field jacket. Orthographic view of photogrammetric model. Scale = 10 cm.

**Figure 21:**
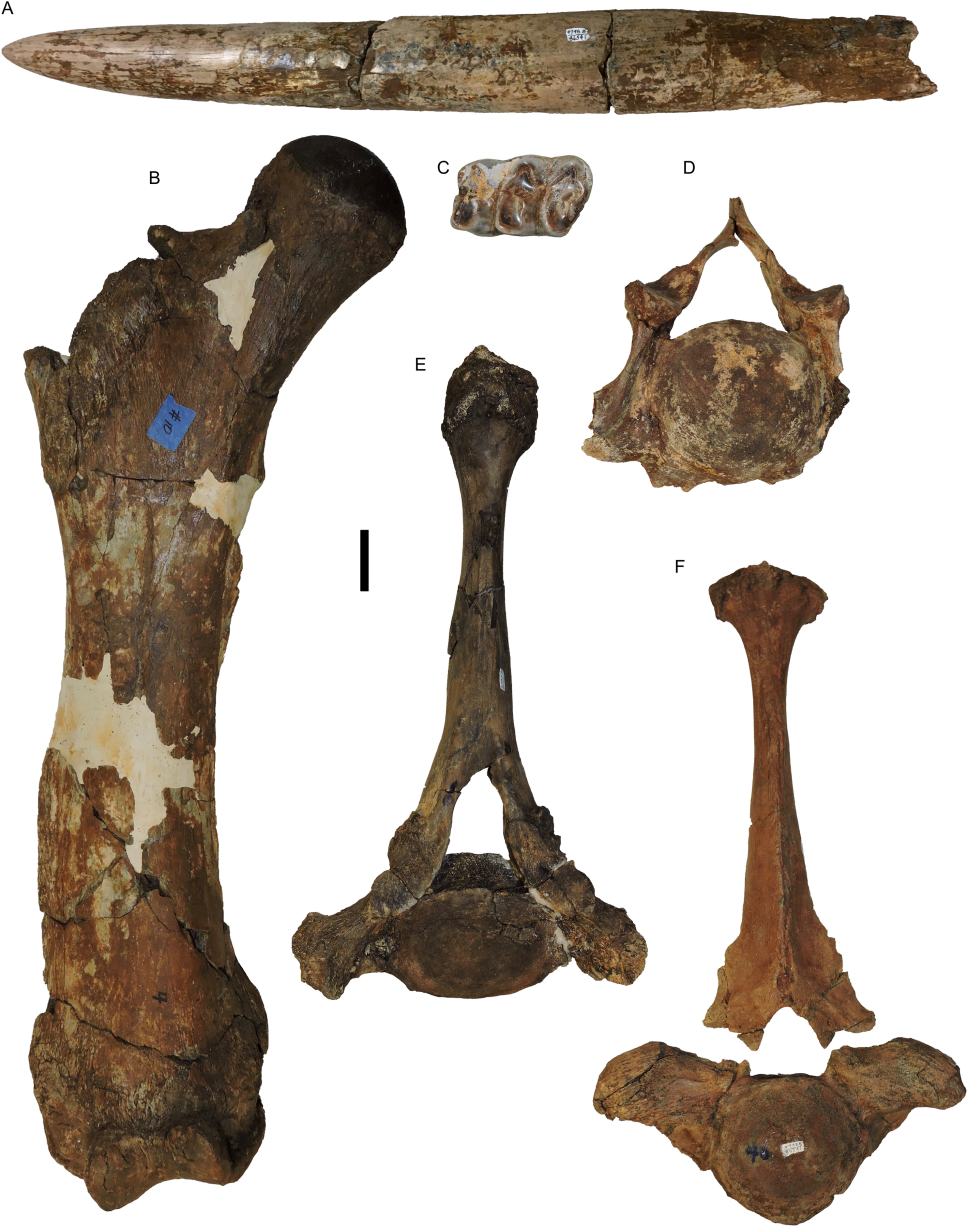
*Mammut pacificus* sp. nov., SDSNH 86541, referred partial skeleton. (A) Left tusk (I1) in lateral view, (B) right femur in anterior view, (C) right m2, occlusal view, (D) cervical vertebra, anterior view, and (E and F) anterior thoracic vertebrae, anterior view. Scale = five cm.

**Figure 22:**
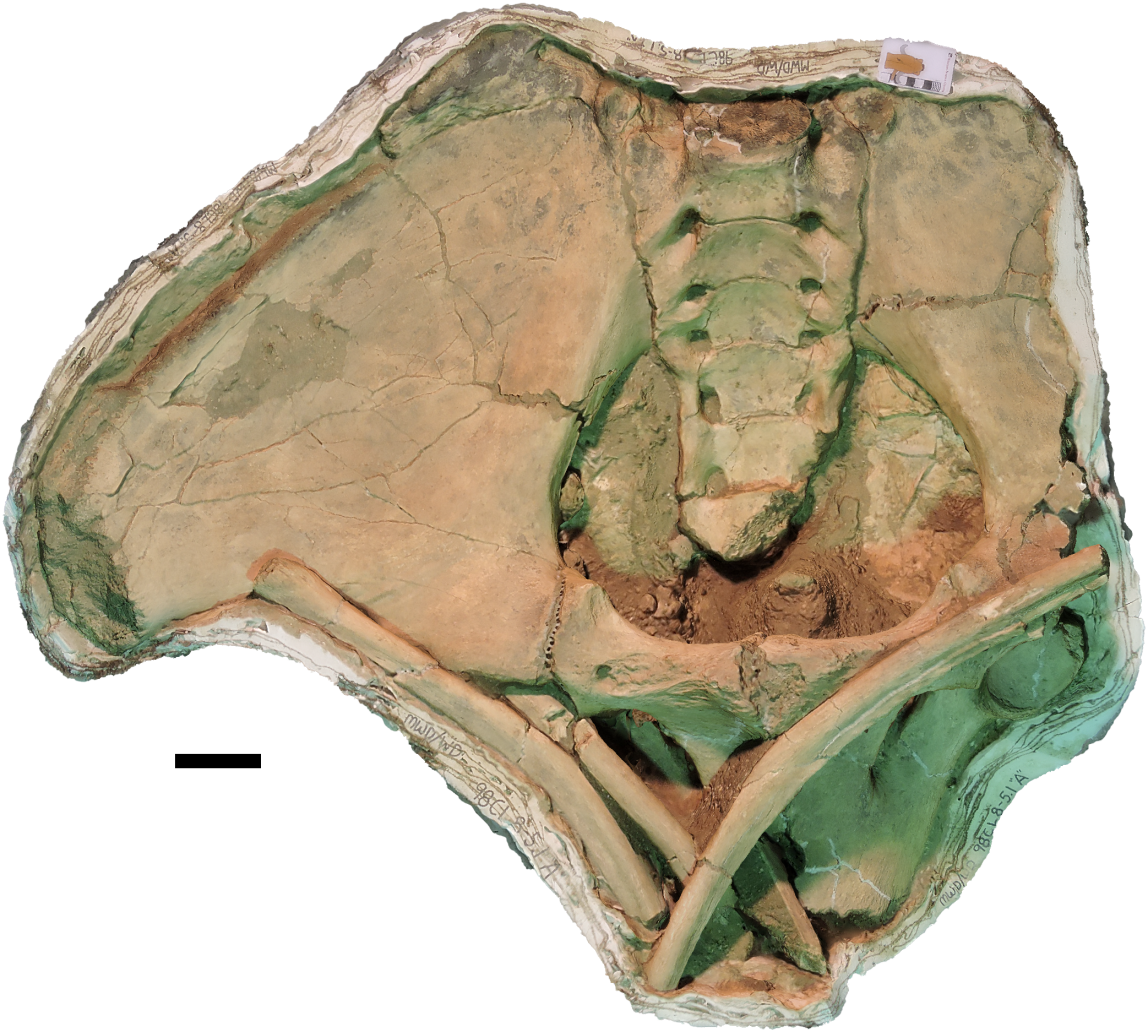
*Mammut pacificus* sp. nov., WSC 9642, referred pelvis. Pelvis in ventral view, with associated ribs. Orthographic view of photogrammetric model. Scale = 10 cm.

**Figure 23:**
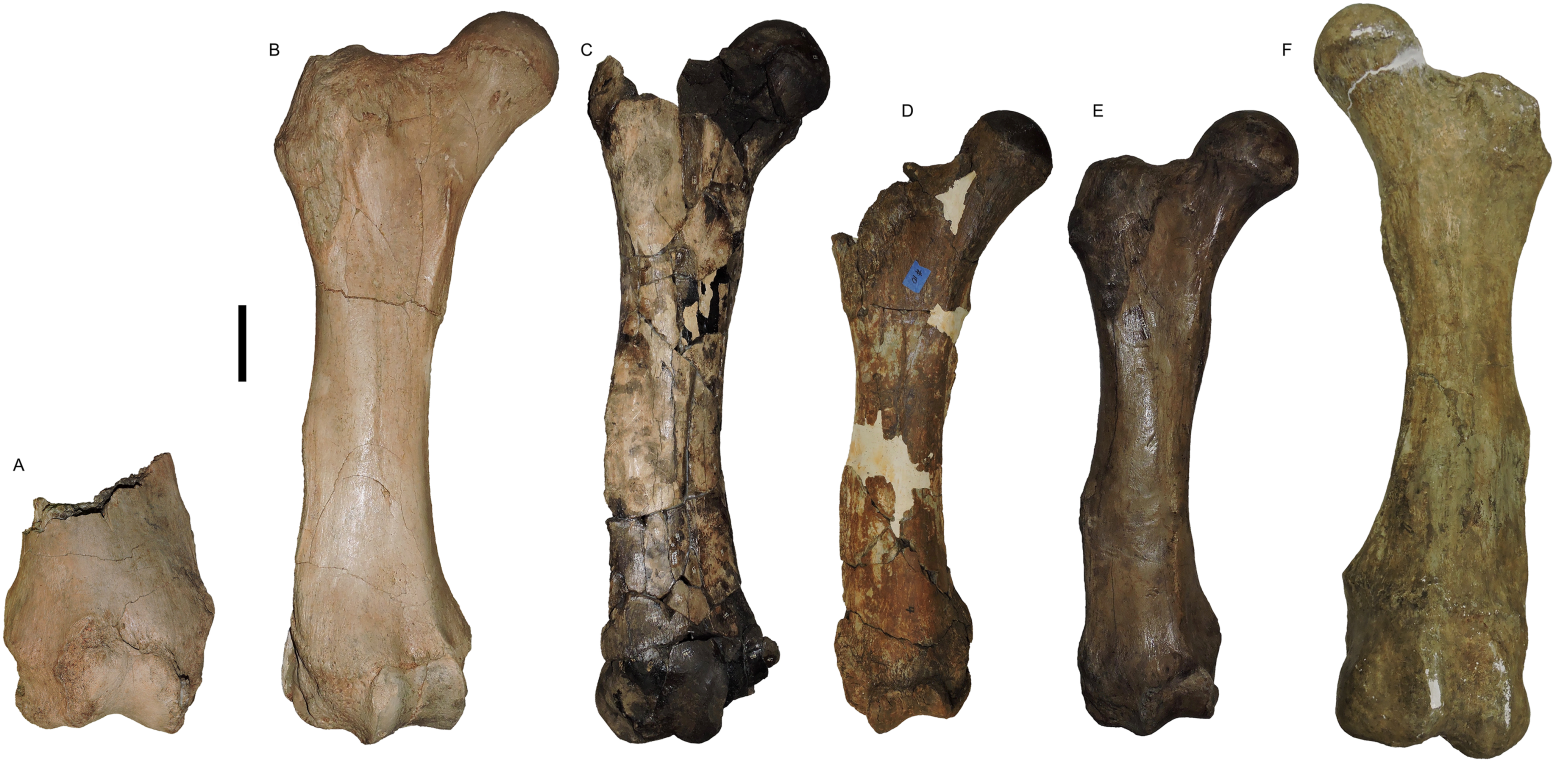
*Mammut pacificus* sp. nov., comparative anterior views of femora. (A) WSC 18743, holotype, distal left femur, (B) WSC 9622, right femur, (C) LACM-HC 86048, right femur, (D) SDSNH 86541, right femur, (E) LACM-HC 1266, right femur, and (F) SBMNH-VP 3343, left femur. Scale = 10 cm.

**Table 1:** Measurements (in mm) of WSC 18743, holotype of *Mammut pacificus* sp. nov.

Length of cranium on midline	1,000
Width of cranium at posterior edge of orbits	650
Width across occipital condyles	206
Width across temporal constriction	400
Length from foramen magnum to internal nares	221
Length from foramen magnum to anterior edge of internal nares	340
Length from anterior edge of internal nares to anterior edge of cranium	660
Palate width between posterior ends of M2s	112
Palate width between posterior ends of M3s	97
Height from top of foramen magnum to top of supraoccipital	319
Length of right tusk around outer curve	1,996
Maximum diameter of tusk	179
Maximum length of mandible	815
Width of mandible across condyles	542
Width of left dentary at anterior end of m3	124
Depth of left dentary at anterior end of m3	165
Width of right dentary at anterior end of m3	127
Depth of right dentary at anterior end of m3	166
Maximum anterior width of centrum of fifth cervical vertebra	144
Midline anterior height of centrum of fifth cervical vertebra	154
Maximum anterior width of centrum of posterior thoracic vertebra (Figs. 3C and 3D)	142
Midline anterior height of centrum of posterior thoracic vertebra (Figs. 3C and 3D)	99
Maximum anterior width of centrum of posterior thoracic vertebra (Figs. 3E and 3F)	154
Midline anterior height of centrum of posterior thoracic vertebra (Figs. 3E and 3F)	103
Maximum anterior width of centrum of first lumbar vertebra	148
Midline anterior height of centrum of first lumbar vertebra	103
Maximum anterior width of centrum of second lumbar vertebra	137
Midline anterior height of centrum of second lumbar vertebra	112
Maximum width of pelvic aperture	505
Minimum width of left ilial shaft	220
Maximum width of pelvis across ilia as preserved	1,460
Distal width of left femur	288

**Table 2:** Referred specimens of *Mammut pacificus* sp. nov., including ontogenetic age estimates and locality information.

Catalog number	Elements	LG/AEY	Locality	County	State	Collected	Figures
WSC 9622	Partial disarticulated skeleton including a partial cranium with right M2–M3, both tusks, both humeri and femora, partial pelvis lacking the sacrum, numerous vertebrae and ribs	LG XX, 34 ± 2 AEY	Diamond Valley Lake, East Dam (97SK6-27.1)	Riverside	California	1997	Figure 6
WSC 8817	Partial skull including the cranium anterior to the orbit with a nearly complete right tusk and left and right M3, and a mandible with left and right m3	LG XXIX, 57 ± 4 AEY	Diamond Valley Lake, West Dam (99KK6-11.5)	Riverside	California	1999	Figure 7
WSC 10844	Anterior portion of a cranium with the proximal portions of both tusks, left and right M2, and the anteriormost loph of left M3, and the proximal end of the right femur	LG XXII; 39 ± 2 AEY (estimate based on M2)	Diamond Valley Lake, West Dam (98CL11-4.1A)	Riverside	California	1998	Figure 8
WSC 19730	Part of the left side of the cranium including the proximal part of the tusk, M2, and M3, and a mandible lacking the left condyle, with left and right m2 and m3	LG XIX, 32 ± 2 AEY	Diamond Valley Lake, West Dam, San Diego Canal (93Q11-18.2)	Riverside	California	1993	Figures 9, 19B
WSC 22587	Partial cranium with left M2 and M3 and right M3, and proximal portions of both tusks	LG XXII, 39 ± 2 AEY	Diamond Valley Lake, West Dam, San Diego Canal (93Q12-17.1)	Riverside	California	1993	Figure 10
WSC 10829	Partial cranium with left M2–M3 and right M2, the proximal portions of both tusks, the posterior portions of both dentaries, and several ribs	LG XXI, 36 ± 2 AEY	Diamond Valley Lake, West Dam (98CS8-8.1)	Riverside	California	1998	Figure 11
LACM 149514	Partial skeleton including a partial cranium with left and right M2 and M3, a partial mandible with left and right m2 and m3, and fragment of the anterior vertebrae, all four limbs, the pelvis and scapulae	LG XX, 34 ± 2 AEY	Simi Valley (V7455)	Ventura	California	2001	
UCMP 114599	Partial palate with left and right M2 and M3	LG XIX, 32 ± 2 AEY	Homestake Mine Road 1 (V6560)	Lassen	California	1922	Figure 12
UCMP 22575	Partial palate with left P4 and M1, and right dP3, dP4, and M1	LG IX, 10 ± 1 AEY	Antioch (V1604)	Contra Costa	California	1916	Figure 13
UCMP 36684	Palate with left and right M3, fragments of left M2, and alveolae for both incisors	LG XIX, 32 ± 2 AEY	Dolan Canyon (V4103)	Alameda	California	1941	Figure 14
WSC 10819	Partial cranium with left and right M2 and M3 and proximal left tusk	LG XXII, 39 ± 2 AEY	Diamond Valley Lake, East Dam, St. John’s Channel (97KMS11-21.2)	Riverside	California	1997	Figure 15
WSC 8904	Partial cranium with left and right M3 and proximal right tusk	LG XXIII, 43 ± 2 AEY	Diamond Valley Lake, East Dam North (96Q4-25.6B)	Riverside	California	1996	Figure 16
WSC 10646	Partial palate with left and right M3 and the posterior portions of left and right M2	LG XIX, 32 ± 2 AEY	Diamond Valley Lake, East Dam Pond (96KMS11-20.2)	Riverside	California	1996	Figure 17
WSC 8917	Partial cranium with left and right M3 and alveolae for both M2s and tusks	LG XVIII or XIX, 30–32 ± 2 AEY	Diamond Valley Lake, East Dam (97JAS11-25.17)	Riverside	California	1997	Figure 18
LACM-HC 87081/87133	Left maxilla fragment with M1, M2, and M3	LG XV, 24 ± 2 AEY	Rancho La Brea	Los Angeles	California		
SBCM 5.3.298	Partial cranium with left and right tusks, M2, and M3, sacrum, and several additional vertebrae and ribs	LG XXI, 36 ± 2 AEY	Perris	Riverside	California		
WSC 180	Juvenile maxilla fragments including left P2 and P4, partial left P3, and partial right P4	LG I or II; 0–0.5 AEY	Diamond Valley Lake, West Dam (95JB5-10.1)	Riverside	California	1995	Figures 24AA, 24II
UCMP RSF 0201	Partial left and right maxillae with dp2–dp3, and partial mandible with left dp2–dp3	LG I or II; 0–0.5 AEY	Rancho La Brea	Los Angeles	California		Figure 19J
WSC 10055	Fragmentary left and right dentaries with left and right m2 and m3, and left M3		Copper Canyon, Murrieta (Irvingtonian)	Riverside	California		
LACM-HC 77/87094/87095/87096/87097	Mandible lacking the left condyle, with left and right m2 and m3, and left and right M2 and M3	LG XX or XXI, 34–36 ± 2 AEY	Rancho La Brea	Los Angeles	California		Figure 19F
LACM 152669	Partial mandible with right m2 and left and right m3	LG XX; 39 ± 2 AEY	Lakes at Thousand Oaks shopping center	Ventura	California	2004	Figure 19D
UCMP 86140	Partial mandible with partial left m2 and partial left and right m3, and the distal end of the left femur	LG XIX, 32 ± 2 AEY	Bengard Ranch (V6903)	San Benito	California	1898	Figure 19E
LACM 130515	Partial mandible with left m2 and right m3	LG XXI, 36 ± 2 AEY	Chorro Creek (V5903)	San Luis Obispo	California	1970	
LC 0260	Partial right dentary with m2 and m3	LG XXIV, 45 ± 2 AEY	Armitage Heights	Orange	California		Figure 19C
LACM-HC 475	Partial mandible with right dp2–dp4, and unerupted m1	LG II or III, 2–3 ± 0.5 AEY	Rancho La Brea	Los Angeles	California		Figure 19K
LACM-HC 87073	Partial mandible with left m2–m3 and right m3	LG XXII, 39 ± 2 AEY	Rancho La Brea	Los Angeles	California		Figure 19G
LACM-HC 38	Mandible with left and right m1, m2, and unerupted m3	LG XII, 18 ± 1 AEY	Rancho La Brea	Los Angeles	California		Figure 19I
LACM-HC 1631	Partial mandible with left and right dp2–dp4, and unerupted m1	LG IV, 2 ± 0.5 AEY	Rancho La Brea	Los Angeles	California		Figure 19M
LACM-P23 26389	Partial mandible with left dp2–dp4	LG III, 1 AEY	Rancho La Brea	Los Angeles	California		Figure 19L
LACM 128927	Partial mandible with left and right m1 and m2	LG XII; 18 ± 1 AEY	Carrizo Plain	San Luis Obispo	California	1978	Figure 19H
UCMP 35129	Partial right dentary with m1 and m2	LG XI or XII, 15–18 ± 1 AEY	McKittrick Pit tar seep (V7139)	Kern	California	1924	
UCMP 212944	Partial mandible		Mokelumne Pipe Ditch	Contra Costa	California		
SBMNH-VP-3341	Partial left dentary with m2–m3	LG XX; 34 ± 2 AEY		Santa Barbara	California		
SBMNH-VP-3342	Partial mandible with left m2–m3	LG XXII; 39 ± 2 AEY		Santa Barbara	California		
USNM 13701	Partial right dentary with m3		American Falls	Power	Idaho		
LACM-HC 87087/87088/87090/87093	Partial left dentary with dp4–m2, right dp4 and m2	LG IX, 10 ± 1 AEY	Rancho La Brea	Los Angeles	California		Figure 19N
SBCM A3005-159	Left dentary with dp2–4		Jurupa Valley	Riverside	California		
SBCM A2658-7	Right dentary fragment with m3		Murrieta (Irvingtonian)	Riverside	California		
SDSNH 116399	Partial skeleton including maxilla fragments with right and left M2 and M3, right dentary fragment with right m2 and m3, tusk fragments, partial axis, third cervical, and fourth cervical vertebrae, and rib fragments	LG XVIII, 30 ± 2 AEY	Robertson Ranch, East Village	San Diego	California	2007	
WSC 7561	Tusk and partial skeleton including at least 15 vertebrae, with the first 11 in articulation, and numerous ribs		Diamond Valley Lake, East Dam (97CB9-12.7)	Riverside	California	1997	Figure 20
SDSNH 86541	Partial skeleton including the complete left tusk, fragments of the right tusk, left m2, right forelimb including humerus, radius, ulna, carpals, and metacarpals, right femur, numerous vertebrae, ribs, and tarsals	LG XXII, 39 ± 2 AEY	Wanis View Estates #5, Oceanside	San Diego	California	2002	Figures 21 and 23D
WSC 8932	Partial skeleton with both tusks, cranial fragments, fragments of M3, partial left humerus, axis, portions of at least four other vertebrae, and rib fragments		Diamond Valley Lake, East Dam (98NB1-27.7)	Riverside	California	1998	
WSC 9642	Partial pelvic girdle missing part of the left ilium		Diamond Valley Lake, West Dam (98CL8-5.1A)	Riverside	California	1998	Figure 22
WSC 13552	Partial vertebral column including parts of the first eight vertebrae, and rib fragments		Diamond Valley Lake, West Dam (98JAS2-3.1)	Riverside	California	1998	
LACM 3014	Left m3		Sun Valley	Los Angeles	California	1956	
UCMP 212936	Right M3		Calaveras Dam (V3937)	Alameda	California	1939	
UCMP 45265	Left M2 and M3	LG XXI, 36 ± 2 AEY	Union Oil Tank Farm (V3428)	Contra Costa	California	1955	
UCMP 41642	Left M3 and right M2 and M3	LG XXII, 39 ± 2 AEY	5-Oaks Ranch (V5213)	Sonoma	California	1952	
UCMP 70139	Right M3		Ducker Ranch (V6517)	Sonoma	California	1965	
UCMP 1569	Right m3		Mormon Gulch (V6538)	Tuolumne	California	1858	
UCMP 1060	Right m3		Gold Springs Gulch (V65516)	Tuolumne	California		
UCMP 8204	Left p2		Potter Creek Cave (V1055)	Shasta	California	1902–1903	
UCMP 5049	Right P2		Potter Creek Cave (V1055)	Shasta	California	1902–1903	
IMNH 39139	m3		Gay Mine	Bingham	Idaho		
IMNH 39156	m3		Gay Mine	Bingham	Idaho		
LACM-HC 1266	Right femur		Rancho La Brea	Los Angeles	California		Figure 23D
LACM-HC 86048	Right femur		Rancho La Brea	Los Angeles	California		Figure 23E
SBMNH-VP 3343	Left femur, partial M3		Carpinteria	Santa Barbara	California		Figure 23F
WSC 9964	Left M1, M2, M3, right M3	LG XVI, 26 ± 2 AEY	Diamond Valley Lake, West Dam (99CS2-15.2B)	Riverside	California	1999	Figures 24B, 24W
WSC 22300	Left M3	∼LG XXIII or XXIV, 43–45 ± 2 AEY	Diamond Valley Lake, West Dam, San Diego Canal (93JB12-10.1)	Riverside	California	1993	

**Location:** Diamond Valley Lake West Dam (Locality number 95Q10-16.1), near Hemet, Riverside County, California. Found in a fluvial deposit, approximately 4.05–4.31 m below the original ground surface. Associated vertebrate remains from the same locality and stratum include a juvenile partial humerus from *Mammut* and a partial tooth from *Mammuthus columbi*, as well as *Urocyon*, *Sylvilagus*, *Scapanus*, *Dipodomys*, *Thomomys*, *Neotoma*, *Microtus*, *Callipepla*, colubrids, and anurans. This specimen was collected from October 17 to November 7, 1995 during the relocation of the San Diego canal in advance of construction of the West Dam (field notes are on file at WSC).

**Age:** Pleistocene, Rancholabrean. Radiocarbon dating of charred organic material associated with holotype specimen WSC 18743 yielded six calibrated ^14^C ages that range from 15.87 ± 0.19 to 16.79 ± 0.28 ka (Springer et al., 2009, 2010). Referred specimen WSC 9622 from Diamond Valley Lake, East Dam (locality number 97SK6-27.1), yielded three calibrated ^14^C ages from charred organic material, plant fibers, and eggshell that are all greater than 46.5 ka. Referred specimen WSC 8932, also from Diamond Valley Lake, East Dam (locality number 98NB1-27.7), yielded one calibrated age from a Succineidae shell of 37.8 ± 1.8 ka. Additional information on ^14^C ages is provided in Table 3.

**Table 3:** Summary of sample information, carbon-14 ages, and calibrated ages of holotype specimen and referred material from Diamond Valley Lake.

Sample #	WSC #	AMS #	Material dated	Treatment^1^	^14^C age (ka BP)	Age (cal ka BP)^2^	*p*^4^
East Dam							
97SK6-27.1	9622	USGS-1039	Charcoal	ABA	43.6 ± 3.2	>46.5^3^	
97SK6-27.1	9622	USGS-1070	Plant fibers	ABA	>42.6	>45.8^3^	1.00
97SK6-27.1xx	9622	USGS-1081	Eggshell (carbonate)	HCl	>44.8	>48.1^3^	1.00
98NB1-27.7	8932	USGS-1195	Succineidae shell	HCl	33.55 ± 0.76	37.8 ± 1.8	1.00
West Dam							
95Q10-16.1	18743	CAMS-28300	Charcoal	ABA	13.44 ± 0.06	16.17 ± 0.22	1.00
95Q10-16.1	18743	CAMS-31081	Charcoal	ABA	13.52 ± 0.06	16.30 ± 0.23	1.00
95Q10-16.1	18743	CAMS-27967	Charcoal	ABA	13.20 ± 0.05	15.87 ± 0.19	1.00
95Q10-16.1	18743	CAMS-28301	Charcoal	ABA	13.43 ± 0.05	16.15 ± 0.19	1.00
95Q10-16.1 (−2726)	18743	USGS-1193	Charcoal	ABA	13.88 ± 0.07	16.79 ± 0.28	1.00
95Q10-16.1 (−2737)	18743	USGS-1194	Charcoal	ABA	13.28 ± 0.07	15.97 ± 0.23	1.00

**Notes:**

Uncertainties for the calibrated ages are given at the 2σ (95%) confidence level. All other uncertainties are given at 1σ (68%).

1 ABA, acid-base-acid; HCl, acid leach.

2 Calibrated ages were calculated using CALIB v.7.1html, IntCal13.14C dataset; limit 50.0 calendar ka B.P. Calibrated ages are reported as the midpoint of the calibrated range. Uncertainties are calculated as the difference between the midpoint and either the upper or lower limit of the calibrated age range, whichever is greater (reported at the 95% confidence level; 2s). Multiple ages are reported when the probability of a calibrated age range exceeds 0.05.

3 Sample ^14^C age or sample ^14^C age plus uncertainty is beyond the limit of the IntCal13 dataset and therefore, cannot be calibrated (Reimer et al., 2013).

4 *p*, probability of the calibrated age falling within the reported range as calculated by CALIB.

**Etymology:** The specific name, *pacificus*, refers to the fact that all currently recognized specimens of this taxon were collected less than 1,000 km from the coast of the Pacific Ocean.

#### Diagnosis

A species of *Mammut* differing from *Mammut americanum* in the following characteristics: M3/m3 significantly narrower relative to length; six fused sacral vertebrae in later ontogenetic stages (usually five in *M. americanum*, with a range of four to six); femur with a greater midshaft diameter relative to length; absence of mandibular tusks and associated alveoli (variably present in *M. americanum*); smaller basal diameter of tusks in males for a given age.

### Description

This description relies primarily on the holotype specimen, WSC 18743, but includes additional referred specimens to provide information on elements that are absent in the holotype, as well as to provide insight into intraspecific and interspecific variability in characters.

#### Cranium

The cranium of WSC 18743 (Fig. 1; M37481) is largely complete. There is some dorsoventral crushing that slightly flattened the dorsal side of the braincase; this crushing does not appear to have affected the ventral half of the cranium. The alveoli for the tusks appear to be crushed as well, particularly on the left side.

In general morphology, the cranium does not differ significantly from that of *Mammut americanum*, when allowing for individual, sexual, and ontogenetic variability. WSC 18743 falls into Laws group XXII (39 ± 2 AEY), which is ontogenetically somewhat older than AMNH 9951 (the “Warren mastodon”) from Newburgh, New York (based on our images of this specimen and the written description in Warren (1852)). The frontals of WSC 18743 more fully cover the temporal fossae in dorsal view than does AMNH 9951, but this may be affected by the dorsoventral crushing in WSC 18743. In any case, this feature exhibits some ontogenetic variability (compare, e.g., AMNH 9951 and AMNH 14535 in Osborn (1936)). Although WSC 18743 and AMNH 9951 both represent male mastodons and are of roughly similar ontogenetic ages, the cranium of WSC 18743 is considerably shorter, with a length of one m (measured along the midline on the dorsal surface) (Table 1). According to Warren (1852), the cranial length of AMNH 9951 is 48 inches (1.23 m).

Osborn (1936) noted that *M. americanum* specimens differed from each other in the position of the tusk alveoli relative to the maxillary tooth rows, a feature that he considered to be sexually dimorphic and related to the larger size of the tusk in the male. In AMNH 14292 (approximately LG XXI), which Osborn (1936) considered a female, and AMNH 17727 (LG V or VI), considered a juvenile male by Osborn (1936), there is a vertical step between the maxillary tooth row and the ventral margin of the premaxilla that forms the tusk alveolus when seen in lateral view (e.g., see Osborn, 1936, Figs. 131 and 132). In contrast, in AMNH 9951 (LG XX or XXI), YPM 12600 (LG XIX or XX), and AMNH 14535 (LG XVI or XVII), all regarded by Osborn (1936) as males, the ventral margin of the premaxilla is only slightly raised above the tooth row, with no distinct step. Two specimens, ISM 71.3.261 (the “Buesching mastodon,” LG XIX) and a UMMP unnumbered specimen from Michigan (the “Fowler Center mastodon,” LG XVI or XVII) are interesting intermediate stages, with a small but distinct step. In IPFW 1 (the “Routsong mastodon” LG XV), an apparent female, the premaxilla is elevated well above the tooth row, but instead of a sharp vertical step there is a more gradual transition from the tooth row to the premaxilla, showing that there is some variability in this feature.

In WSC 18743 there is a distinct vertical step between the premaxilla and the maxillary tooth row (Fig. 1C). Even though this is a mature specimen (LG XXII), and interpreted as a male based on pelvic measurements, this condition is more similar to *M. americ*anum specimens considered by Osborn (1936) to be females or juvenile males. As tusks continue to grow late in mastodon ontogeny, it might be expected that in males there would be some ontogenetic variability in the expression of this character. Indeed, referred specimen WSC 8817, which has an estimated age of 55 ± 4 AEY (LG XXVIII), has tusk alveoli that extend ventrally to just above the tooth row (Fig. 7). This suggests that *M. pacificus* may have reached its maximum tusk size later in ontogeny than *M. americanum*, although a full analysis of this possibility will require a detailed examination of tusk diameters, premaxilla morphology, and ontogenetic ages of both *M. pacificus* and *M. americanum*.

The left and right maxillary tooth rows in WSC 18743 and in referred specimens WSC 10829 (a possible male, based on tusk size) and WSC 8917 (a possible female, based on tusk size) are parallel or nearly so (Figs. 1B, 11 and 18C). This is in contrast to some other specimens referred to this new species, including WSC 22587 (Fig. 10), UCMP 114599 (Fig. 12), and UCMP 36684 (Fig. 14), in which the tooth rows are posteriorly convergent. Osborn (1936) noted that similar variability in *M. americanum* does not appear to correlate with sex or ontogeny. *M. pacificus* seems to exhibit similar variability, with both morphologies found across several ontogenetic ages, and at least the parallel tooth row form found in both males and females.

#### Mandible

The mandible of WSC 18743 (Fig. 2) is relatively elongate, with a smoothly downturned anterodorsal surface of the mandibular symphysis as is typical of *Mammut*. The ascending ramus is angled slightly posteriorly, with the anterior margin forming an angle of about 110° with the tooth row. The anterior margin of the ascending ramus extends vertically to from a somewhat recurved coronoid process. Posteriorly the ascending ramus expands transversely into the mandibular condyle, the dorsal margin of which is slightly higher than the apex of the coronoid process. There is a partially resorbed alveolus for the m1 on the right side only. There are no lower tusks, and no trace of alveoli for them. There are two pathologies on the right dentary, a bony growth near the anterior tip of uncertain origin and a deep groove with swollen margins on the lateral surface adjacent to the anterior end of m2. The more posterior pathology appears to have resulted from an impact injury. Fisher (2009) proposed that male specimens of *M. americanum* show frequent injuries that resulted from intraspecies combat, which is one possible source of the pathologies observed in WSC 18743. The entire mandible is asymmetrical, with the right tooth row shifted posteriorly relative to the left. Given that the degree of asymmetry is variable across the length of the dentary, it is interpreted as a response to the trauma suffered by the right dentary, rather than due to taphonomic processes.

There is some variability in the shape of the ascending ramus among different specimens that has no direct correlation with age or body size. WSC 19730 (LG XIX), WSC 8817 (LG XXIX), LACM-P23 26389 (LG III), LACM-HC 475 (LG II or II), and UCMP RSF 0201 (LG I or II) have posteriorly inclined ascending rami similar to WSC 18743, while in LACM 152669 (LG XXII), LACM 128927 (LG XII), LACM-HC 87073 (LG XXII), SBMNH-VP-3341 (LG XX), and SBMNH-VP-3342 (LG XXII), the anterior margins of the ascending rami are nearly vertical. There is also discernible variability in the angle between the dentaries in dorsal view, from nearly parallel to posteriorly divergent (Fig. 19). This divergence is most noticeable in the portion of the dentaries posterior to the tooth row and may become less pronounced with age as the mandible increases in length (compare, e.g., Figs. 19A and 19M). However, this character may be subject to subtle taphonomic alteration, especially if the mandible is lying on its side when buried.

Thirteen specimens of *M. pacificus* preserve the mandibular symphysis, and range from LG I to LG XXIX (WSC 18743, WSC 19730, WSC 8817, LACM 128927, LACM-HC 38, LACM-HC 87073, LACM-HC 475, LACM-HC 1631, LACM-P23 26389, UCMP 86140, UCMP 212944, UCMP RSF 0201, and SBMNH-VP-3342) (Fig. 19); all of these specimens lack mandibular tusks or any trace of tusk alveoli. Moreover, at the two California localities that have produced numerous mastodon remains (Rancho La Brea and Diamond Valley Lake), no isolated elements consistent with mandibular tusks have been recovered. This is a remarkably stable pattern when compared to *M. americanum*. While the geographic, temporal, sexual, and ontogenetic distribution of mandibular tusks in *M. americanum* is not fully understood (Laub, 1999, 2009), there does appear to be a trend toward reduction of the mandibular tusks over time. Every specimen of *M. americanum* from the early Rancholabrean deposits at Ziegler Reservoir in Colorado has mandibular tusks, which are often quite large (Cherney, Fisher & Rountrey, 2017; Fisher et al., 2014). Mandibular tusks are also present in the late Rancholabrean AMNH 9951. Alveoli are present in PRI 49820, but are absent in YPM12600. Green (2006) noted that mandibular tusks were always present in Irvingtonian *M. americanum* (*n* = 11) from Florida, but only present 27% of the time in Rancholabrean individuals (*n* = 22). An exact binomial test of goodness-of-fit comparing the *M. pacificus* lower tusk occurrence rate (0/13) to the Florida Rancholabrean *M. americanum* occurrence rate (6/22) yielded a *p*-value of 0.026, indicating that *M. pacificus* does exhibit mandibular tusks with lower frequency than *M. americanum* based on these data. Unfortunately, no Irvingtonian mastodon specimen from California has a preserved mandibular symphysis.

#### Tusks

The complete right tusk and distal third of the left tusk of WSC 18743 are preserved (Figs. 1F and 1G). Where the tusks emerge from the premaxillae they are angled ventrally about 5° relative to the maxillary tooth row, and laterally about 20° from the midline. The right tusk spirals gently both medially and dorsally, so that the tips would have pointed straight anteriorly with the neck at maximum ventral deflection, and vertically with the neck at maximum dorsal deflection. The tusks exhibit differential wear at the tip, with heavier wear on the right tusk.

Numerous other Diamond Valley Lake specimens were recovered with portions of one or both tusks, including WSC 9622, WSC 8817 (Fig. 7), WSC 10844 (Fig. 8), WSC 19730 (Fig. 9), WSC 22578 (Fig. 10), WSC 10829 (Fig. 11), WSC 10819 (Fig. 15), WSC 8904 (Fig. 16), WSC 7561, and WSC 8932. Because tusks grow continuously and can be influenced by environmental variables, evaluation of sexual dimorphism, and gender identification based on tusks must be approached with caution. That said, at least for *M. americanum*, at any given ontogenetic age females tend to have smaller tusks than males all (Fisher, 2008, 2009; Smith, 2010), so it is possible to make a tentative gender identification provided tusk (or alveolus) size and Laws Group can be determined. Available data suggest that in *M. americanum* adult females with tusk circumferences >36 cm and males with circumferences <39 cm occurred rarely if at all (Fisher, 2008, 2009; Smith, 2010). Remarkably, all of the tusks associated with maxillary teeth from Diamond Valley Lake appear to represent adult males, based on their sizes and ontogenetic ages. There are several smaller tusks, not associated with maxillary teeth that may represent either females or juvenile males, although we cannot rule out juvenile mammoths as a possibility.

Referred specimen SDSNH 86541 (Fig. 21A) appears to represent an adult female (LG XX), with very small (78 mm maximum diameter), straight tusks. While SDSNH 86541 is a particularly small individual, the tusk morphology is consistent with the larger WSC 3795 and WSC 3817. Another referred specimen, WSC 8917 (Fig. 18), is also an apparent adult female (LG XVIII or XIX). While lacking tusks, the maximum diameter of the alveolus for the left tusk is approximately 71 mm.

#### Dentition

Like other crown proboscideans, mammutids exhibit horizontal tooth replacement, progressing through a total of six teeth in each quadrant. Although there is a considerable amount of variation in dental morphology, in both *M. pacificus* and *M. americanum* dP2/dp2 and dP3/dp3 are bilophodont (although in both taxa dP3/dp3 often develop a posterior shelf/partial third loph, as described in Green & Hulbert (2005)), dP4/dp4, M1/m1, and M2/m2 are trilophodont. Of the 39 *M. pacificus* M3s examined in this study, 15 are tetralophodont and 24 are pentalophodont. All known *M. pacificus* m3s are pentalophodont. Summary measurements of teeth for both *M. pacificus* and *M. americanum* are listed in Tables 4–15.

**Table 4:** Aggregate measurements of M3 of *M. americanum* and *M. pacificus* by location.

State/Province/Country	*n*	Mean maximum length	Median maximum length	SD	Max	Min	Mean maximum width	Median maximum width	SD	Max	Min	Mean L/W	Median L/W	SD	Max	Min
California	39	166.94	167.41	12.74	197.00	142.90	85.35	84.83	6.08	104.26	73.08	1.98	1.96	0.14	2.33	1.69
Alaska	2	157	157.00	10.32	164.30	149.7	95.58	95.58	2.02	97.0	94.2	1.64	1.64	0.07	1.69	1.59
Arizona	1	188.2	188.20		188.20	188.2	98.2	98.20		98.2	98.2	1.92	1.92		1.92	1.92
Colorado	4	163.50	163.50	0.58	164.00	163.00	99.30	99.25	1.70	101.20	97.50	1.65	1.65	0.03	1.67	1.61
Florida	15	177.35	177.30	15.20	197.40	143.20	99.07	100.60	7.51	110.40	82.20	1.79	1.79	0.11	1.95	1.59
Georgia	1	184.00	184.00		184.00	184.00	104.00	104.00		104.00	104.00	1.77	1.77		1.77	1.77
Illinois	2	184.75	184.75	13.79	194.50	175.00	106.69	106.69	6.63	111.37	102.00	1.73	1.73	0.02	1.75	1.72
Indiana	8	178.93	185.00	18.70	203.00	154.00	99.86	99.75	7.16	108.00	87.85	1.75	1.71	0.14	1.95	1.60
Louisiana	2	179.80	179.80	23.76	196.60	163.00	108.20	108.20	13.86	118.00	98.40	1.66	1.66	0.01	1.67	1.66
Missouri	22	181.92	181.20	18.57	214.50	144.30	100.71	101.40	7.94	114.50	86.60	1.80	1.80	0.08	1.94	1.63
New York	1	172.20	172.20		172.20	172.20	100.20	100.20		100.20	100.20	1.72	1.72		1.72	1.72
North Carolina	5	169.06	174.70	14.36	185.00	152.80	95.18	96.00	6.85	102.60	86.00	1.78	1.74	0.09	1.93	1.71
Ohio	4	180.03	181.75	25.67	205.40	151.20	102.30	104.85	8.97	110.00	89.50	1.76	1.74	0.14	1.93	1.61
Texas	3	171.68	167.00	11.74	185.04	163.00	99.30	97.00	9.66	109.90	91.00	1.73	1.72	0.05	1.79	1.68
Utah	2	151.50	151.50	0.71	152.00	151.00	85.50	85.50	2.12	87.00	84.00	1.77	1.77	0.05	1.81	1.74
Washington	1	161.63	161.63		161.63	161.63	88.82	88.82		88.82	88.82	1.82	1.82		1.82	1.82
Yukon	5	153.04	154.98	5.27	159.56	87.29	88.44	88.56	0.80	89.52	87.29	1.73	1.73	0.05	1.81	1.68
Mexico	1	158.00	158.00		158.00	158.00	83.00	83.00		83.00	83.00	1.90	1.90		1.90	1.90
*M. americanum*	79	174.68	174.85	17.70	214.50	143.20	98.60	99.20	8.12	118.00	82.20	1.77	1.77	0.10	1.95	1.59
*M. pacificus*	39	166.94	167.41	12.74	197.00	142.90	85.35	84.83	6.08	104.26	73.08	1.98	1.96	0.14	2.33	1.69

**Table 5:** Aggregate measurements of m3 of *M. americanum* and *M. pacificus* by location.

State/Province/Country	*n*	Mean maximum length	Median maximum length	SD	Max	Min	Mean maximum width	Median maximum width	SD	Max	Min	Mean L/W	Median L/W	SD	Max	Min
California	23	184.08	186.36	12.74	202.58	159.74	82.55	82.66	6.24	94.03	68.00	2.25	2.25	0.14	2.44	1.95
Idaho	1	163.70	163.70		163.70	163.70	76.70	163.70		76.70	76.70	2.13	2.13		2.13	2.13
Alaska	2	166.45	166.45	29.06	187.00	145.9	90.11	90.11	15.40	101.0	79.2	1.85	1.85	0.01	1.85	1.84
Arizona	1	171.1	171.10		171.10	171.1	82.8	82.80		82.8	82.8	2.07	2.07		2.07	2.07
Colorado	9	182.44	174.80	11.39	202.40	171.00	95.97	95.70	6.09	106.80	87.80	1.91	1.93	0.17	2.23	1.64
Florida	23	181.59	183.00	14.35	216.50	155.00	96.12	95.90	5.45	111.60	89.10	1.89	1.93	0.13	2.04	1.63
Illinois	8	188.03	187.65	11.14	199.90	167.00	100.56	101.50	7.50	108.50	85.00	1.87	1.84	0.05	1.96	1.82
Indiana	9	185.02	188.50	14.00	202.30	164.00	100.13	99.00	5.02	108.00	91.70	1.85	1.81	0.12	2.04	1.66
Kansas	2	195.00	195.00	11.31	203.00	187.00	100.74	100.74	6.41	105.27	96.20	1.94	1.94	0.01	1.94	1.93
Kentucky	6	188.60	190.80	14.98	202.70	165.00	100.45	99.95	8.83	116.50	90.90	1.88	1.88	0.10	2.00	1.73
Louisiana-Mississippi	9	194.29	188.00	15.21	226.50	183.30	101.40	101.20	5.56	110.00	93.00	1.92	1.89	0.10	2.06	1.77
Missouri	20	189.10	187.45	14.41	213.00	162.00	98.32	98.20	6.31	110.60	86.00	1.92	1.91	0.05	2.07	1.87
Nebraska	4	181.18	181.05	3.63	184.70	177.90	100.10	100.20	1.27	101.50	98.50	1.81	1.81	0.05	1.87	1.75
New Mexico	3	166.33	168.00	12.58	178.00	153.00	89.00	93.00	8.26	94.50	79.50	1.87	1.91	0.08	1.92	1.78
New York	1	196.70	196.70		196.70	196.70	97.60	97.60		97.60	97.60	2.02	2.02		2.02	2.02
North Carolina	4	180.45	188.90	18.10	190.60	153.40	91.63	92.15	3.00	94.40	87.80	1.97	2.01	0.15	2.10	1.75
Ohio	4	191.30	191.20	20.59	222.20	160.60	99.40	101.85	8.72	106.90	87.00	1.92	1.89	0.12	2.08	1.82
Quebec	1	136.00	136.00		136.00	136.00	79.00	79.00		79.00	79.00	1.72	1.72		1.72	1.72
Texas	5	188.80	195.00	13.37	200.00	168.00	99.40	100.00	5.08	106.00	93.00	1.90	1.91	0.06	1.95	1.81
Utah	2	169.50	169.50	0.71	170.00	169.00	82.50	82.50	2.12	84.00	81.00	2.06	2.06	0.04	2.09	2.02
Virginia	1	165.60	165.60		165.60	165.60	89.50	89.50		89.50	89.50	1.85	1.85		1.85	1.85
Washington	2	165.14	165.14	0.76	165.68	164.60	81.80	81.80	5.35	85.58	78.01	2.02	2.02	0.12	2.11	1.94
West Virginia	1	177.00	177.00		177.00	177.00	97.00	97.00		97.00	97.00	1.82	1.82		1.82	1.82
Yukon	2	160.40	160.40	3.75	163.05	157.75	81.88	81.88	0.66	82.34	81.41	1.96	1.96	0.03	1.98	1.94
Mexico	2	172.05	172.05	4.36	175.13	168.97	80.88	80.88	1.02	81.60	80.16	2.13	2.13	0.08	2.18	2.07
*M. americanum*	121	183.54	184.00	15.68	226.50	136.00	96.46	96.80	7.73	116.50	78.01	1.91	1.91	0.11	2.23	1.63
*M. pacificus*	24	183.15	185.81	13.17	202.58	159.74	82.29	82.41	6.22	94.03	68.00	2.24	2.25	0.13	2.44	1.95

**Table 6:** Aggregate measurements of M2 of *M. americanum* and *M. pacificus* by location.

State/Province/Country	*n*	Mean maximum length	Median maximum length	SD	Max	Min	Mean maximum width	Median maximum width	SD	Max	Min	Mean L/W	Median L/W	SD	Max	Min
California	27	103.40	103.00	6.20	115.00	92.10	79.71	80.00	4.79	86.30	65.40	1.32	1.32	0.09	1.55	1.09
Arizona	2	118.35	118.35	6.58	123.00	113.7	86.8	86.80	1.70	88.0	85.6	1.36	1.36	0.05	1.40	1.33
Colorado	11	123.35	123.50	6.08	135.90	113.00	94.42	94.10	2.95	98.40	89.30	1.31	1.31	0.05	1.39	1.21
Florida	12	115.35	113.05	6.98	129.80	107.10	90.63	90.40	4.90	97.70	81.50	1.27	1.27	0.08	1.44	1.14
Indiana	5	116.10	119.50	9.04	121.00	100.00	90.80	90.00	4.70	95.50	84.00	1.28	1.27	0.06	1.35	1.19
Kentucky	2	124.70	124.70	1.27	125.60	123.80	92.20	92.20	0.14	92.30	92.10	1.35	1.35	0.02	1.36	1.34
Louisiana	3	122.67	122.60	1.70	124.40	121.00	103.13	99.80	13.22	117.70	91.90	1.20	1.21	0.14	1.33	1.06
Missouri	25	121.66	121.90	7.19	136.50	110.10	92.61	91.80	6.40	103.70	79.70	1.32	1.31	0.05	1.42	1.24
New York	1	111.50	111.50		111.50	111.50	88.40	88.40		88.40	88.40	1.26	1.26		1.26	1.26
North Carolina	2	105.45	105.45	0.21	136.50	105.30	91.25	91.25	1.63	92.40	90.10	1.16	1.16	0.02	1.17	1.14
Ohio	2	115.20	115.20	13.29	124.60	105.80	90.95	90.95	2.05	92.40	89.50	1.27	1.27	0.17	1.39	1.15
Oregon	1	111.6	111.60		111.6	111.6	79.7	79.70		79.7	79.7	1.40	1.40		1.40	1.40
Utah	1	100.00	100.00		100.00	100.00	80.00	80.00		80.00	80.00	1.25	1.25		1.25	1.25
*M. americanum*	67	119.13	120.00	8.04	136.50	100.00	92.17	91.90	6.30	117.70	79.70	1.29	1.30	0.08	1.44	1.06
*M. pacificus*	27	103.40	103.00	6.20	115.00	92.10	79.71	80.00	4.79	86.30	65.40	1.32	1.32	0.09	1.55	1.26

**Table 7:** Aggregate measurements of m2 of *M. americanum* and *M. pacificus* by location.

State/Province/Country	*n*	Mean maximum length	Median maximum length	SD	Max	Min	Mean maximum width	Median maximum width	SD	Max	Min	Mean L/W	Median L/W	SD	Max	Min
California	25	107.82	107.00	8.21	125.00	88.62	75.44	75.47	5.29	85.90	65.00	1.44	1.44	0.10	1.60	1.21
Arizona	1	105.70	105.70		105.70	105.70	74.90	74.90		74.90	74.90	1.41	1.41		1.41	1.41
Colorado	10	118.07	115.95	8.50	134.90	108.60	85.40	85.50	5.20	95.30	78.90	1.38	1.38	0.09	1.54	1.23
Florida	11	110.72	110.00	7.57	124.40	99.60	86.36	84.80	7.21	100.60	78.60	1.28	1.28	0.04	1.34	1.23
Indiana	5	112.36	114.50	9.29	122.00	102.00	89.38	90.00	8.85	98.00	78.90	1.26	1.24	0.06	1.33	1.17
Kentucky	1	129.20	129.20		129.20	129.20	94.50	94.50		94.50	94.50	1.37	1.37		1.33	1.37
Mississippi	1	115.00	115.00		115.00	115.00	87.00	87.00		87.00	87.00	1.32	1.32		1.32	1.32
Missouri	30	118.17	118.15	7.70	133.20	101.30	87.94	89.00	4.26	95.20	80.80	1.34	1.33	0.06	1.51	1.25
New Mexico	1	98.00	98.00		98.00	98.00	68.00	68.00		68.00	68.00	1.44	1.44		1.44	1.44
North Carolina	3	117.43	117.00	4.37	122.00	113.30	87.80	88.00	1.41	89.10	86.30	1.34	1.36	0.06	1.39	1.27
Ohio	2	113.80	113.80	1.70	115.00	112.60	91.70	91.70	5.52	95.60	87.80	1.24	1.24	0.06	1.28	1.20
Texas	2	105.50	105.50	10.61	113.00	98.00	89.00	89.00	2.83	91.00	87.00	1.18	1.18	0.08	1.24	1.13
Utah	1	101.00	101.00		101.00	101.00	76.00	76.00		76.00	76.00	1.33	1.33		1.33	1.33
Virginia	2	95.75	95.75	17.32	108.00	83.50	71.80	71.80	3.11	74.00	69.60	1.34	1.34	0.30	1.55	1.13
Washington	2	106.82	106.82	3.34	109.18	104.45	74.07	74.07	0.28	74.27	73.87	1.44	1.44	0.05	1.48	1.41
Yukon	1	92.99	92.99		92.99	92.99	74.33	74.33		74.33	74.33	1.25	1.25		1.25	1.25
Mexico	1	105.25	105.25		105.25	105.25	72.91	72.91		72.91	72.91	1.44	1.44		1.44	1.44
*M. americanum*	74	114.17	115.00	9.63	134.90	83.50	85.85	86.85	6.96	100.60	68.00	1.33	1.33	0.08	1.55	1.13
*M. pacificus*	25	107.82	107.00	8.21	125.00	88.62	75.44	75.47	5.29	85.90	65.00	1.44	1.44	0.10	1.60	1.21

**Table 8:** Aggregate measurements of M1 of *M. americanum* and *M. pacificus* by location.

State/Province/Country	*n*	Mean maximum length	Median maximum length	SD	Max	Min	Mean maximum width	Median maximum width	SD	Max	Min	Mean L/W	Median L/W	SD	Max	Min
California	5	86.16	84.00	6.41	97.10	80.70	67.84	68.00	1.69	69.90	66.00	1.27	1.25	0.07	1.39	1.22
Alaska	2	82.30	82.30	1.56	83.40	81.20	68.10	68.10	9.62	74.90	61.30	1.22	1.22	0.15	1.32	1.11
Colorado	9	100.77	96.40	7.68	112.80	94.00	82.12	80.40	5.39	90.20	76.50	1.23	1.23	0.04	1.29	1.17
Florida	9	88.24	86.90	7.27	101.00	77.90	72.13	70.40	4.96	81.30	65.50	1.22	1.20	0.08	1.35	1.13
Ohio	1	89.00	89.00		89.00	89.00	75.80	75.80		75.80	75.80	1.17	1.17		1.17	1.17
Oregon	1	104.53	104.53		104.53	104.53	76.67	76.67		76.67	76.67	1.36	1.36		1.36	1.36
Virginia	2	92.25	92.25	0.35	92.50	92.00	73.50	73.50	6.36	78.00	69.00	1.26	1.26	0.11	1.34	1.18
*M. americanum*	24	93.49		9.37	112.80	77.90	76.00		7.14	90.20	61.30	1.23		0.07	1.36	1.11
*M. pacificus*	5	86.16	84.00	6.41	97.10	80.70	67.84	68.00	1.69	69.90	66.00	1.27	1.25	0.07	1.39	1.22

**Table 9:** Aggregate measurements of m1 of *M. americanum* and *M. pacificus* by location.

State/Province/Country	*n*	Mean maximum length	Median maximum length	SD	Max	Min	Mean maximum width	Median maximum width	SD	Max	Min	Mean L/W	Median L/W	SD	Max	Min
California	6	94.45	94.17	9.03	108.50	84.20	62.86	62.27	4.38	69.30	57.83	1.50	1.50	0.11	1.63	1.35
Colorado	6	90.78	91.30	2.87	94.20	86.00	68.80	68.40	2.07	71.10	65.60	1.32	1.31	0.04	1.38	1.27
Florida	6	86.80	86.15	2.09	90.60	84.70	64.78	63.95	3.42	68.90	61.30	1.34	1.35	0.06	1.41	1.25
Indiana	1	98.50	98.50		98.50	98.50	74.50	74.50		74.50	74.50	1.32	1.32		1.32	1.32
Ohio	1	87.80	87.80		87.80	87.80	67.60	67.60		67.60	67.60	1.30	1.30		1.30	1.30
Virginia	4	90.80	90.50	4.65	96.20	86.00	70.48	72.45	4.35	73.00	64.00	1.29	1.31	0.09	1.38	1.18
Washington	1	76.78	76.78		76.78	76.78	60.60	60.60		60.60	60.60	1.27	1.27		1.27	1.27
*M. americanum*	19	89.04	88.00	4.89	98.50	76.78	67.69	68.30	4.23	74.50	60.60	1.32	1.31	0.06	1.41	1.18
*M. pacificus*	6	94.45	94.17	9.03	108.50	84.20	62.86	62.27	4.38	69.30	57.83	1.50	1.50	0.11	1.63	1.35

**Table 10:** Aggregate measurements of dP4 of *M. americanum* and *M. pacificus* by location.

State/Province/Country	*n*	Mean maximum length	Median maximum length	SD	Max	Min	Mean maximum width	Median maximum width	SD	Max	Min	Mean L/W	Median L/W	SD	Max	Min
California	2	71.51	71.51	0.86	72.12	70.90	52.82	52.82	2.67	54.70	50.93	1.36	1.36	0.08	1.42	1.30
Colorado	3	75.43	73.50	5.27	81.40	71.40	62.57	63.10	2.94	65.20	59.40	1.21	1.20	0.04	1.25	1.16
Florida	7	72.76	72.90	2.23	76.50	69.60	60.80	59.80	2.47	64.50	58.10	1.20	1.19	0.04	1.24	1.15
Ohio	1	73.60	73.60		73.60	73.60	62.50	62.50		62.50	62.50	1.18	1.18		1.18	1.18
*M. americanum*	11	73.56	73.30	3.17	81.40	69.60	61.44	62.00	2.48	65.20	58.10	1.20	1.19	0.03	1.25	1.15
*M. pacificus*	2	71.51	71.51	0.86	72.12	70.90	52.82	52.82	2.67	54.70	50.93	1.36	1.36	0.08	1.42	1.30

**Table 11:** Aggregate measurements of dp4 of *M. americanum* and *M. pacificus* by location.

State/Province/Country	*n*	Mean maximum length	Median maximum length	SD	Max	Min	Mean maximum width	Median maximum width	SD	Max	Min	Mean L/W	Median L/W	SD	Max	Min
California	6	71.10	67.90	14.87	87.31	58.10	46.93	47.00	7.71	56.79	36.30	1.52	1.54	0.08	1.60	1.40
Arizona	1	75.00	75.00		75.00	75.00	56.00	56.00		56.00	56.00	1.34	1.34		1.34	1.34
Colorado	5	75.68	77.40	7.72	82.00	63.10	56.92	57.40	3.00	60.20	53.40	1.33	1.39	0.16	1.43	1.05
Florida	5	73.10	74.60	6.33	79.00	62.40	53.06	53.20	2.80	56.80	49.10	1.38	1.36	0.10	1.51	1.27
Kentucky	1	89.60	89.60		89.60	89.60	69.70	69.70		69.70	69.70	1.29	1.29		1.29	1.29
Ohio	1	74.17	74.17		74.17	74.17	65.27	65.27		65.27	65.27	1.14	1.14		1.14	1.14
Virginia	1	71.00			71.00	71.00	52.00			52.00	52.00	1.37	1.37		1.37	1.37
*M. americanum*	14	75.26	74.80	7.06	89.60	62.40	56.63	55.15	5.54	69.70	49.10	1.33	1.37	0.12	1.51	1.05
*M. pacificus*	6	71.10	67.90	14.87	87.31	58.10	46.93	47.00	7.71	56.79	36.30	1.52	1.54	0.08	1.60	1.40

**Table 12:** Aggregate measurements of dP3 of *M. americanum* and *M. pacificus* by location.

State/Province/Country	*n*	Mean maximum length	Median maximum length	SD	Max	Min	Mean maximum width	Median maximum width	SD	Max	Min	Mean L/W	Median L/W	SD	Max	Min
California	2	42.75	42.75	0.25	42.93	42.57	38.41	38.41	0.30	38.62	38.20	1.11	1.11	0.02	1.12	1.10
Arizona	1	47.00	47.00		47.00	47.00	45.00	45.00		45.00	45.00	1.04	1.04		1.04	1.04
Colorado	3	46.03	48.10	3.84	48.40	41.60	45.57	46.30	1.72	46.80	43.60	1.01	1.03	0.05	1.04	0.95
Florida	4	44.50	45.20	2.35	46.50	41.10	43.60	44.00	3.61	47.00	39.40	1.02	1.01	0.07	1.11	0.96
Louisiana	1	48.00	48.00		48.00	48.00	47.00	47.00		47.00	47.00	1.02	1.02		1.02	1.02
Ohio	1	45.70	45.70		45.70	45.70	47.50	47.50		47.50	47.50	0.96	0.96		0.96	0.96
Oregon	1	40.56	40.56		40.56	40.56	35.35	35.35		35.35	35.35	1.15	1.15		1.15	1.15
Virginia	5	44.88	45.70	3.12	48.10	41.40	44.96	43.70	3.27	50.00	42.30	1.00	0.98	0.08	1.12	0.90
*M. americanum*	16	45.11	45.70	2.86	48.40	40.56	44.42	45.60	3.60	50.00	35.35	1.02	1.03	0.07	1.15	0.90
*M. pacificus*	2	42.75	42.75	0.25	42.93	42.57	38.41	38.41	0.30	38.62	38.20	1.11	1.11	0.02	1.12	1.10

**Table 13:** Aggregate measurements of dp3 of *M. americanum* and *M. pacificus* by location.

State/Province/Country	*n*	Mean maximum length	Median maximum length	SD	Max	Min	Mean maximum width	Median maximum width	SD	Max	Min	Mean L/W	Median L/W	SD	Max	Min
California	5	45.15	45.15	1.76	46.39	43.90	34.06	34.57	2.85	37.33	29.70	1.36	1.34	0.08	1.48	1.27
Arizona	1	46.00	46.00		46.00	46.00	42.00	42.00		42.00	42.00	1.10	1.10		1.10	1.10
Colorado	2	48.75	48.75	2.05	50.20	47.30	44.70	44.70	1.13	45.50	43.90	1.09	1.09	0.02	1.10	1.08
Florida	3	43.43	43.60	2.15	45.50	41.20	38.57	38.60	0.55	39.10	38.00	1.13	1.15	0.07	1.18	1.05
Ohio	1	48.55	48.55		48.55	48.55	45.03	45.03		45.03	45.03	1.08	1.08		1.08	1.08
Virginia	1	47.50	47.50		47.50	47.50	52.00	52.00		52.00	52.00	0.91	0.91		0.91	0.91
*M. americanum*	8	46.23	46.65	2.85	50.20	41.20	43.02	42.95	4.67	52.00	38.00	1.08	1.09	0.08	1.18	0.91
*M. pacificus*	5	45.15	43.90	1.76	46.39	43.90	34.06	34.57	2.85	37.33	29.70	1.36	1.34	0.08	1.48	1.27

**Table 14:** Aggregate measurements of dP2 of *M. americanum* and *M. pacificus* by location.

State/Province/Country	*n*	Mean maximum length	Median maximum length	SD	Max	Min	Mean maximum width	Median maximum width	SD	Max	Min	Mean L/W	Median L/W	SD	Max	Min
California	3	30.83	30.84	0.28	31.10	30.55	28.18	27.62	1.92	30.31	26.60	1.10	1.12	0.08	1.17	1.01
Florida	4	34.83	34.90	1.36	36.40	33.10	34.83	35.00	2.29	35.40	30.60	1.03	1.02	0.04	1.08	0.99
Ohio	1	33.60	33.60		33.60	33.60	37.20	37.20		37.20	37.20	0.90	0.90		0.90	0.90
Virginia	1	35.00	35.00		35.00	35.00	34.50	34.50		34.50	34.50	1.01	1.01		1.01	1.01
*M. americanum*	6	34.65	34.85	1.17	36.40	33.10	34.62	35.00	2.19	37.20	30.60	1.00	2.93	0.06	1.08	0.90
*M. pacificus*	3	30.83	30.84	0.28	31.10	30.55	28.18	27.62	1.92	30.31	26.60	1.10	1.12	0.08	1.17	1.01

**Table 15:** Aggregate measurements of dp2 of *M. americanum* and *M. pacificus* by location.

State/Province/Country	*n*	Mean maximum length	Median maximum length	SD	Max	Min	Mean maximum width	Median maximum width	SD	Max	Min	Mean L/W	Median L/W	SD	Max	Min
California	5	28.16	27.86	1.56	30.32	26.60	23.85	24.29	3.17	27.61	19.10	1.24	1.22	0.12	1.39	1.10
Arizona	1	35.00	35.00		35.00	35.00	31.00	31.00		31.00	31.00	1.13	1.13		1.13	1.13
Colorado	3	34.87	35.20	1.04	35.70	33.70	29.47	29.50	1.85	31.30	27.60	1.19	1.14	0.09	1.29	1.12
Florida	2	31.40	31.40	0.14	31.50	31.30	26.25	26.25	1.77	27.50	25.00	1.20	1.20	0.08	1.25	1.15
Ohio	1	32.20	32.20		32.20	32.20	33.13	33.13		33.13	33.13	0.97	0.97		0.97	0.97
*M. americanum*	7	33.51	33.70	1.85	35.70	31.30	29.29	29.50	2.78	33.13	25.00	1.15	1.14	0.10	1.29	0.97
*M. pacificus*	5	28.16	27.86	1.56	30.32	26.60	23.85	24.29	3.17	27.61	19.10	1.24	1.22	0.12	1.39	1.10

**Figure 24:**
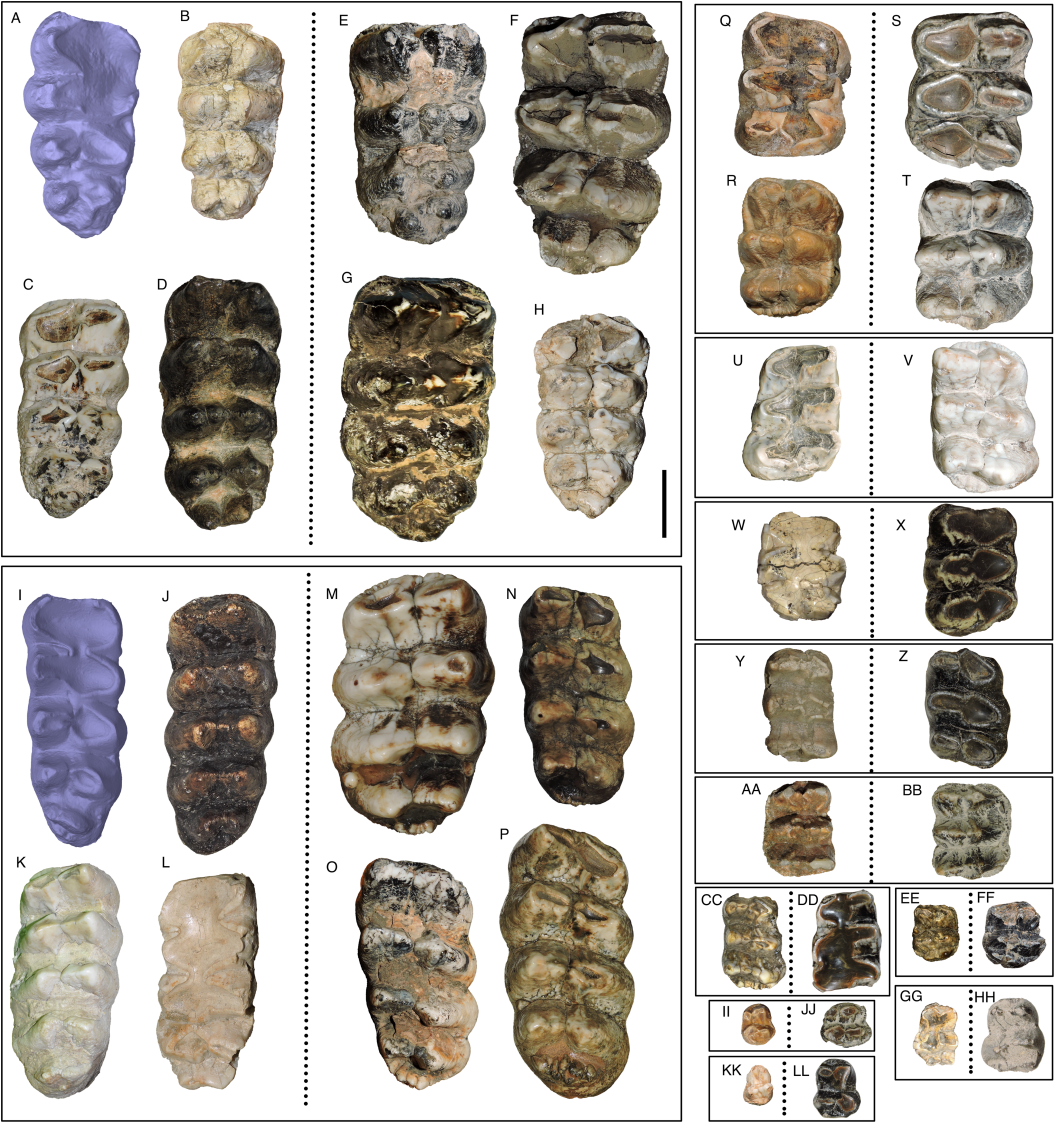
Comparison of sample teeth from *Mammut pacificus* and *Mammut americanum*. (A–D) M3 of *M. pacificus*, (E–H) M3 of *M. americanum*. Note the presence of a fifth loph in (C and D), and the weak to absent cingulum in all four *M. pacificus* specimens (contrast with the strong cingulum development in F). (I–L) m3 of *M. pacificus*, (M–P) m3 of *M. americanum*. Again, note the absence or weak development of a cingulum in (I–L), and the variable morphology of the posterior to the fourth lophid, particularly in *M. americanum*. (Q and R) M2 of *M. pacificus*, (S and T) M2 of *M. americanum*. Note the weak cingulum development in (Q) and strong development in (R). (U) m2 of *M. pacificus*, (V) m2 of *M. americanum*, (W) M1 of *M. pacificus*, (X) M1 of *M. americanum*, (Y) m1 of *M. pacificus*, and (Z) m1 of *M. americanum*. (AA) dP4 of *M. pacificus*, (BB) dP4 of *M. americanum*, (CC) dp4 of *M. pacificus*, (DD) dp4 of *M. americanum*, (EE) dP3 of *M. pacificus*, (FF) dP3 of *M. americanum*. Note the partial third loph in both taxa, (GG) dp3 of *M. pacificus*, (HH) dp3 of *M. americanum*. Note the partial third lophid in both taxa. (II) dP2 of *M. pacificus*, (JJ) dP2 of *M. americanum*, (KK) dp2 of *M. pacificus*, and (LL) dp2 of *M. americanum*. (A) WSC 18743, *M. pacificus* holotype right M3, orthographic view of digital model, Riverside County, California, (B) WSC 9964, *M. pacificus* left M3, Riverside County, California, (C) WSC 10829, *M. pacificus* left M3, Riverside County, California, (D) UCMP 212936, *M. pacificus* right M3, Alameda County, California, (E) DMNH 69327, *M. americanum* left M3, Pitkin County, Colorado, (F) LACM 154685, *M. americanum* left M3, Allen County, Indiana, (G) LSUMG-V 17071, *M. americanum* right M3, West Feliciana Parish, Louisiana, (H) CMC VP 145, *M. americanum* right M3, Clermont County, Ohio, (I) WSC 18743, *M. pacificus* holotype right m3, orthographic view of digital model, Riverside County, California, (J) LACM HC 87072, *M. pacificus* left m3, Los Angeles County, California, (K) WSC 19730, *M. pacificus* left m3, Riverside County, California, (L) LACM 152669, *M. pacificus* left m3, Ventura County, California, (M) CMC VP 1120, *M. americanum* left m3, Hamilton County, Ohio, (N) G47, *M. americanum* right m3, Pinellas County, Florida, (O) JMM VP 20, *M. americanum* right m3, Wayne County, Indiana, (P) LACM 154598, *M. americanum* right m3, Cowley County, Kansas, (Q) SBCM 5.3.298, *M. pacificus* right M2, Riverside County, California, (R) UCMP 1564, *M. pacificus* right M2, Tuolumne County, California, (S) DMNH 69331, *M. americanum* left M2, Pitkin County, Colorado, (T) G25650, *M. americanum* right M2, Darke County, Ohio, (U) LACM 130515, *M. pacificus* left m2, San Luis Obispo County, California, (V) G25650, *M. americanum* right m2, Darke County, Ohio, (W) WSC 9964, *M. pacificus* left M1, Riverside County, California, (X) DMNH 70776, *M. americanum* right M1, Pitkin County, Colorado, (Y) LACM 128927, *M. pacificus* right m1, San Luis Obispo County, California, and (Z) DMNH 70775, *M. americanum* right m1, Pitkin County, Colorado. (AA) WSC 180, *M. pacificus* left dP4, Riverside County, California, (BB) G25693, *M. americanum* right dP4, Darke County, Ohio, (CC) LACM HC 475, *M. pacificus* right dp4, Los Angeles County, California, (DD) DMNH 70785, *M. americanum* left dp4, Pitkin County, Colorado, (EE) LACM P23 4766, *M. pacificus* right dP3, Los Angeles County, California, (FF) VMNH 51132, *M. americanum* left dP3, Smyth County, Virginia, (GG) LACM HC 1631, *M. pacificus* left dp3, Los Angeles County, California, (HH) DMNH 70794, *M. americanum* left dp3, Pitkin County California, (II) WSC 180, *M. pacificus* left dP2, Riverside County, California, (JJ) G25693, *M. americanum* left dP2, Darke County, Ohio, (KK) UCMP 8204, *M. pacificus* left dp2, Shasta County, California, and (LL) DMNH 70795, *M. americanum* right dp2, Pitkin County, Colorado. Scale = 10 cm.

WSC 18743 includes both left and right M2/m2 and M3/m3. Both the upper and lower second molars are heavily worn with only the margins of the lophs remaining. The M3/m3s show lighter but still heavy wear, especially on the anterior lophs and lophids (Figs. 24A and 24I). While the M3s of WSC 18743 are tetralophodont, more than half of known *M. pacificus* M3s are pentalophodont, although the fifth loph varies a great deal in size and shape (Figs. 24C and 24D). In the holotype the transverse valleys between lophs are open and the enamel is smooth with no cingulum except on the anterior and anterolabial edges of the M3s. A cingulum is also absent or only weakly developed on the M3 and m3 in all referred specimens of *M. pacificus* (Figs. 24B–24D and 24J–24L). The cingulum is generally poorly developed on the M3/m3 of *M. americanum* as well, except the anterior margin of the tooth, but there are some individuals with a strongly developed cingulum (e.g., Fig. 24F). In *M. pacificus* teeth anterior to M3/m3 frequently have some development of a cingulum and more rugose enamel, although the expression of these features varies across individuals. All known *M. pacificus* teeth are more similar in texture to the “smooth variety” teeth of *M. americanum* associated with spruce/deciduous forests, rather than the “rugged variety” associated with pine-parkland habitats (King & Saunders, 1984). However, “smooth variety” *M. americanum* teeth tend to be larger than both “rugged variety” *M. americanum* teeth and *M. pacificus* teeth (King & Saunders, 1984).

Only limited data (e.g., femoral measurements) provide body size estimates for *M. pacificus*, but available specimens suggest that tooth size may not correlate closely, if at all, with body size. For example, the m2 of WSC 18743 (an apparent male) has a length of 103.6 mm, while the m2 of SDSNH 86541 (Fig. 21C) (a presumed female) is only slightly longer at 104.6 mm. Yet the distal femur width of WSC 18743 is 288 mm, compared to only 203 mm in SDSNH 86541. Given the inability to determine gender from isolated teeth, comparative body size estimates between *Mammut* specimens should be approached with caution if based on isolated teeth.

The most remarkable feature of the dentition of *M. pacificus* is the narrowness of the crowns, particularly in the third molars (Figs. 24I–24P and 26; Tables 4 and 5; Table S1). The length:width (L:W) ratio of the m3 in WSC 18743 is 2.44 (left) and 2.46 (right). This pattern of narrow m3s is seen in multiple specimens of *M. pacificus*, with an average L:W ratio of 2.24 (*N* = 24; SD = 0.13; max = 2.44, min = 1.95). In contrast, the L:W ratio in *M. americanum* is only 1.91 (*N* = 121; SD = 0.11; max = 2.23, min = 1.63) (Figs. 26C and 26D; Table S2). While there is overlap in the L/W ranges between the taxa, the differences are significant (*T*-test value *p* < 0.001).

**Figure 25:**
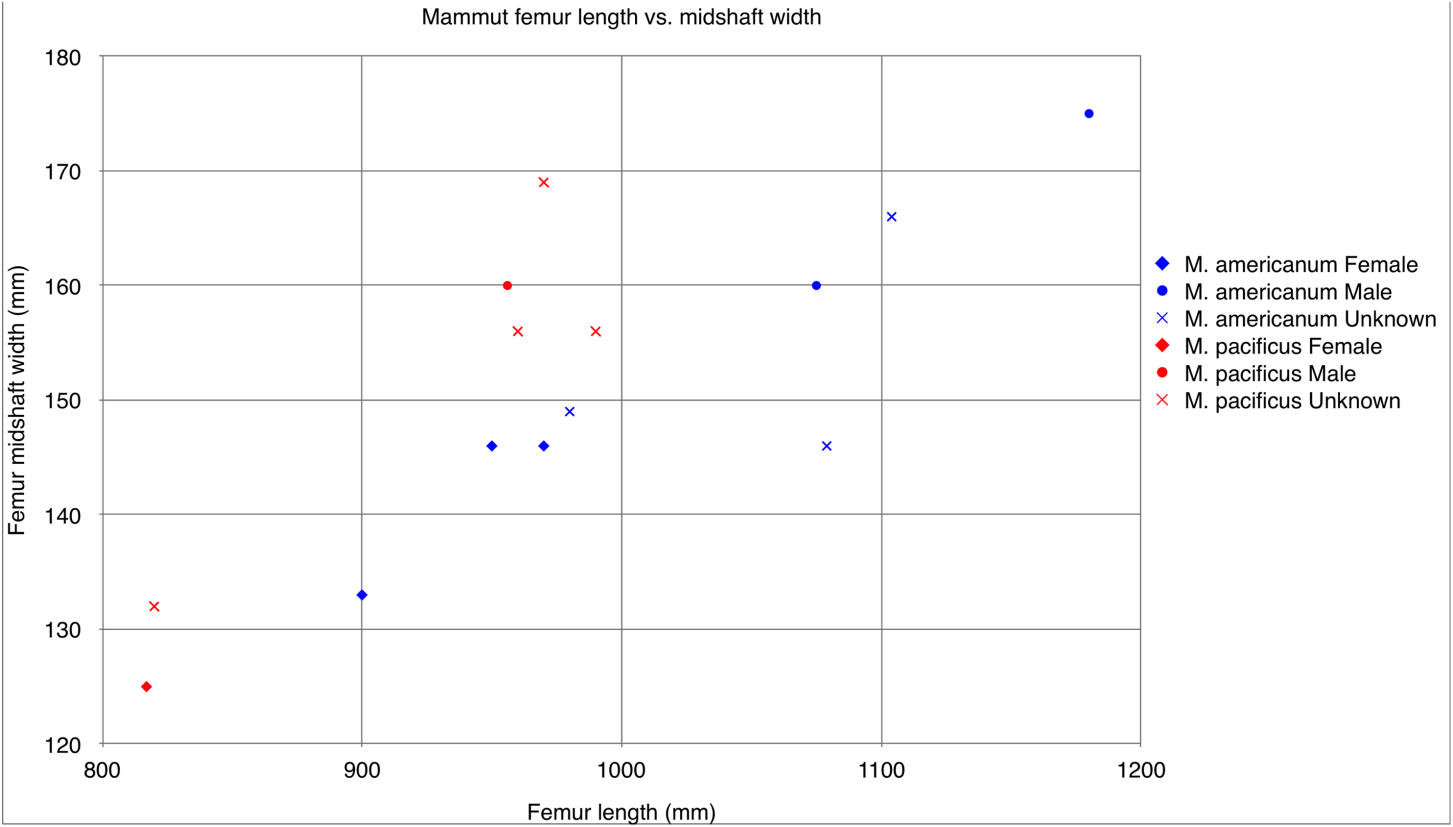
*Mammut* femur length vs. mid shaft width. Comparison of length vs. width measurements in *Mammut pacificus* (red) and *Mammut americanum* (blue). Measurements follow Hodgson et al. (2008a).

**Figure 26:**
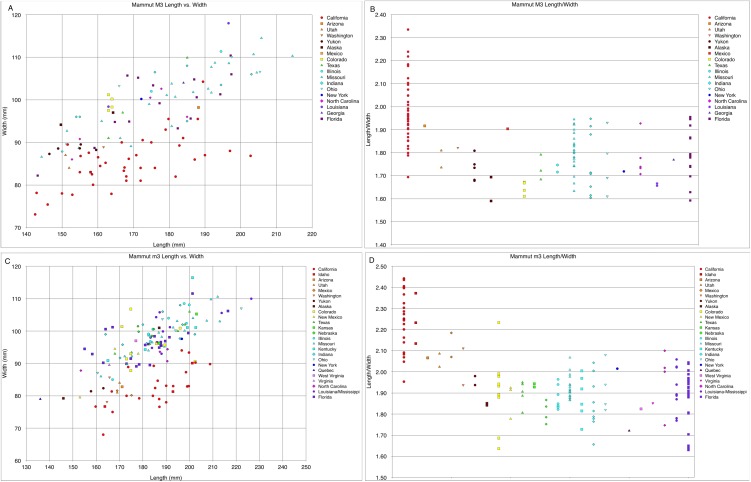
Length and width comparisons of *Mammut* M3 and m3, by state/province/country. (A) Length vs. width of *Mammut* M3. *M. pacificus* is indicated in red, (B) length/width of *Mammut* M3, organized by state. *M. pacificus* is indicated in red, (C) length vs. width of *Mammut* m3. *M. pacificus* is indicated in red, and (D) length/width of *Mammut* m3, organized by state. *M. pacificus* is indicated in red. In (B and D) higher values indicate relatively narrower teeth. In (B) note that the California sample has much higher values than any other location, and that the all the teeth with M3 L:W values ≥2.00 are from California. In (D) note that the California and Idaho samples are similar to each other and have much higher values than any other location.

The same pattern is apparent in the M3s (Figs. 24A–24H), in which the *M. pacificus* average L:W ratio is 1.98 (*N* = 39; SD = 0.14; max = 2.33, min = 1.69) while the *M. americanum* average L:W ratio is 1.77 (*N* = 79; SD = 0.10; max = 1.95, min = 1.59). As with the m3s, these differences are significant (*T*-test value *p* < 0.001). The variation in loph count in M3 does affect L:W ratio in *M. pacificus*. In the 24 pentalophodont M3s L/W = 2.02, while in the 15 tetralophodont M3s L/W = 1.91 (*T*-test value *p* = 0.007). However, the frequent presence of a fifth loph alone is insufficient to explain the difference in L:W ratios between *M. pacificus* and *M. americanum*. Even when considering only tetralophodont *M. pacificus* M3s, *M. pacificus* still has significantly higher L:W ratios than *M. americanum* (1.91–1.77, *T*-test value *p* < 0.001).

While the L:W ratios differ between *M. pacificus* and *M. americanum*, there is little difference in the length of the teeth. The average length of m3 is nearly identical in the two taxa (183.15 mm in *M. pacificus*, 183.54 mm in *M. americanum*, Mann–Whitney test *p* = 0.978; Table 5). The average length of M3 is significantly greater in *M. americanum* (174.68–166.94 mm, Mann–Whitney test *p* = 0.038; Table 4), apparently due to several very large *M. americanum* M3s (the longest *M. americanum* M3 is 17.5 mm longer than the longest *M. pacificus* specimen, while the shortest *M. americanum* specimen is only 0.3 mm longer than the shortest *M. pacificus* specimen) (Table 4).

The pattern of narrow crowns in *M. pacificus* is also apparent in Irvingtonian specimens. Four Irvingtonian m3s referred to *M. pacificus*, including two from Idaho and two from California, have L:W ratios ranging from 2.23 to 2.38 (average = 2.32), even more narrow than the Rancholabrean average (2.24) (Table S1). Only a single Irvingtonian *M. pacificus* M3 has been identified, for which L/W = 2.00. Green (2006) provided measurements of a number of Irvingtonian *M. americanum* specimens from Florida. Four m3s had an average L/W of 1.93, and four m3s had an average L/W of 1.77, very similar to the Rancholabrean *M. americanum* averages of 1.91 and 1.77 (Table S2). While the data are limited, it appears that the disparity in M3/m3 proportions between *M. pacificus* and *M. americanum* was present through the entire Pleistocene.

**Figure 27:**
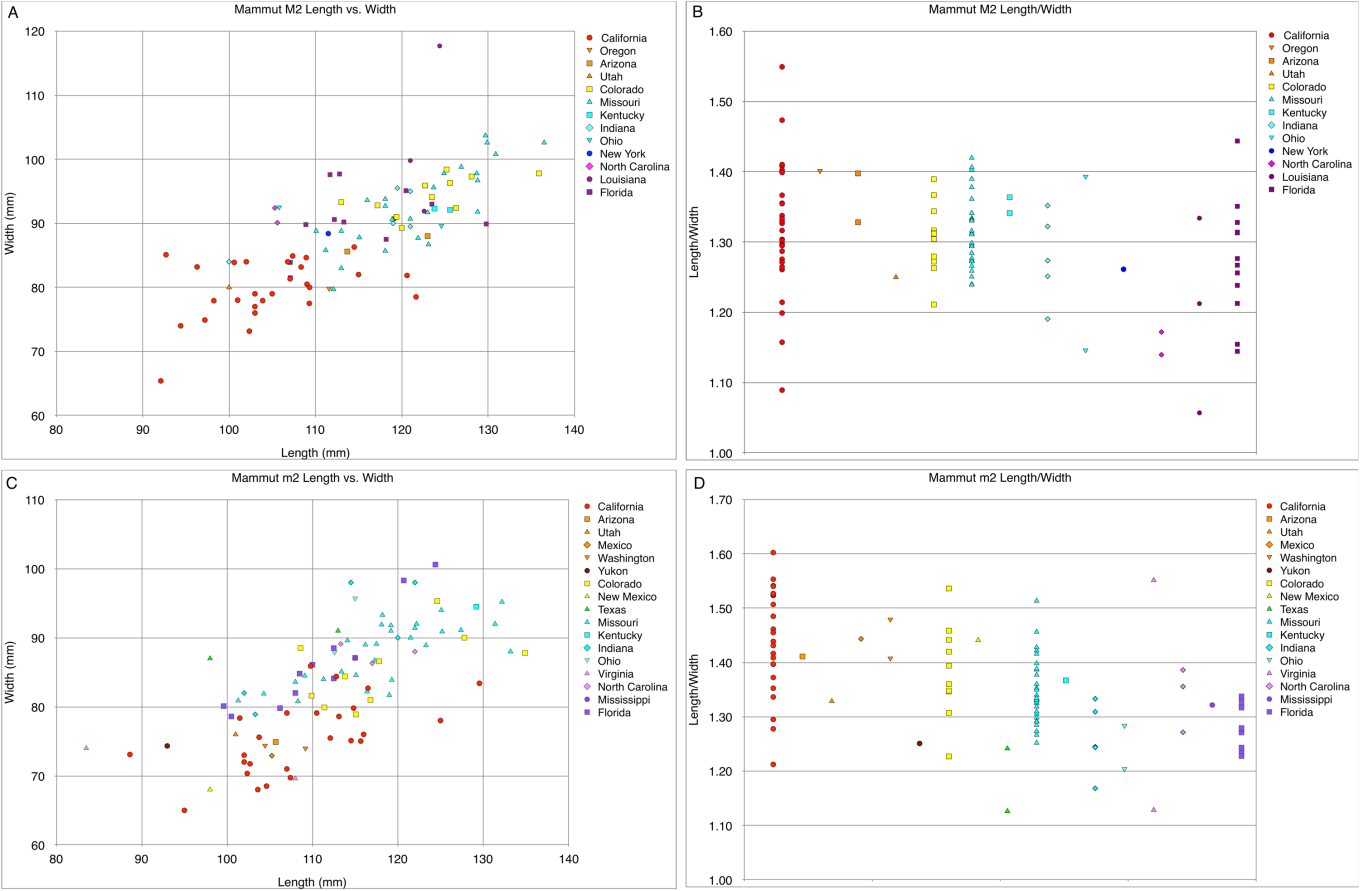
Length and width comparisons of *Mammut* M2 and m2, by state/province/country. (A) Length vs. width of *Mammut* M2. *M. pacificus* is indicated in red, (B) length/width of *Mammut* M2, organized by state. *M. pacificus* is indicated in red, (C) length vs. width of *Mammut* m2. *M. pacificus* is indicated in red, and (D) length/width of *Mammut* m2, organized by state. *M. pacificus* is indicated in red. Note that in (B) and (D), there is little difference in the L:W values between any of the locations, including California. This indicates that M2/m2 proportions do not differ between *M. pacificus* and *M. americanum*.

**Figure 28:**
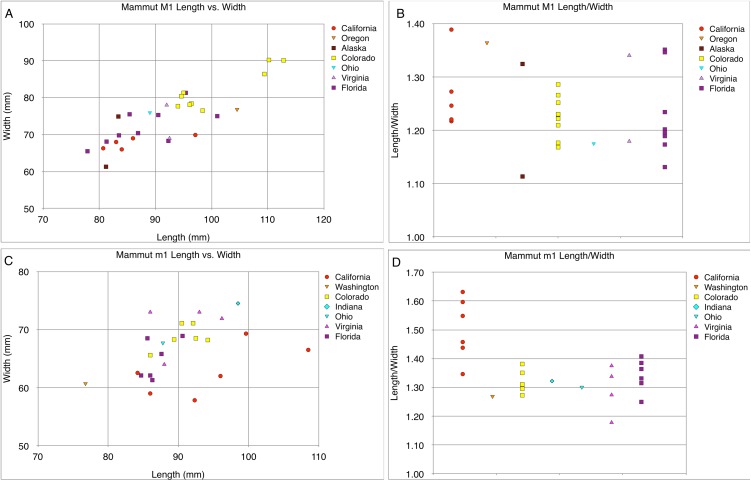
Length and width comparisons of *Mammut* M1 and m1, by state/province/country. (A) Length vs. width of *Mammut* M1. *M. pacificus* is indicated in red, (B) length/width of *Mammut* M1, organized by location. *M. pacificus* is indicated in red, (C) length vs. width of *Mammut* m1. *M. pacificus* is indicated in red, and (D) length/width of *Mammut* m1, organized by location. *M. pacificus* is indicated in red.

The narrow M3/m3 crown of *M. pacificus* relative to *M. americanum* is not as obvious in other tooth positions (Figs. 24Q–24LL and 27–31; Tables 6–15), although in some cases small sample size is an issue. Even with large sample sizes, M2 and M1 of *M. pacificus* do not significantly differ from those of *M. americanum* in L:W ratio (1.32–1.29 for M2, and 1.27 to 1.23 for M1) (Figs. 27B and 28B). Nevertheless, within the data set used in this study, for 11 out of 12 tooth positions, the highest L:W ratio measured was a California tooth attributed to *M. pacificus*. In the lone exception, dP3, there are only two known teeth from California, and one of these had the second-highest L:W ratio.

**Figure 29:**
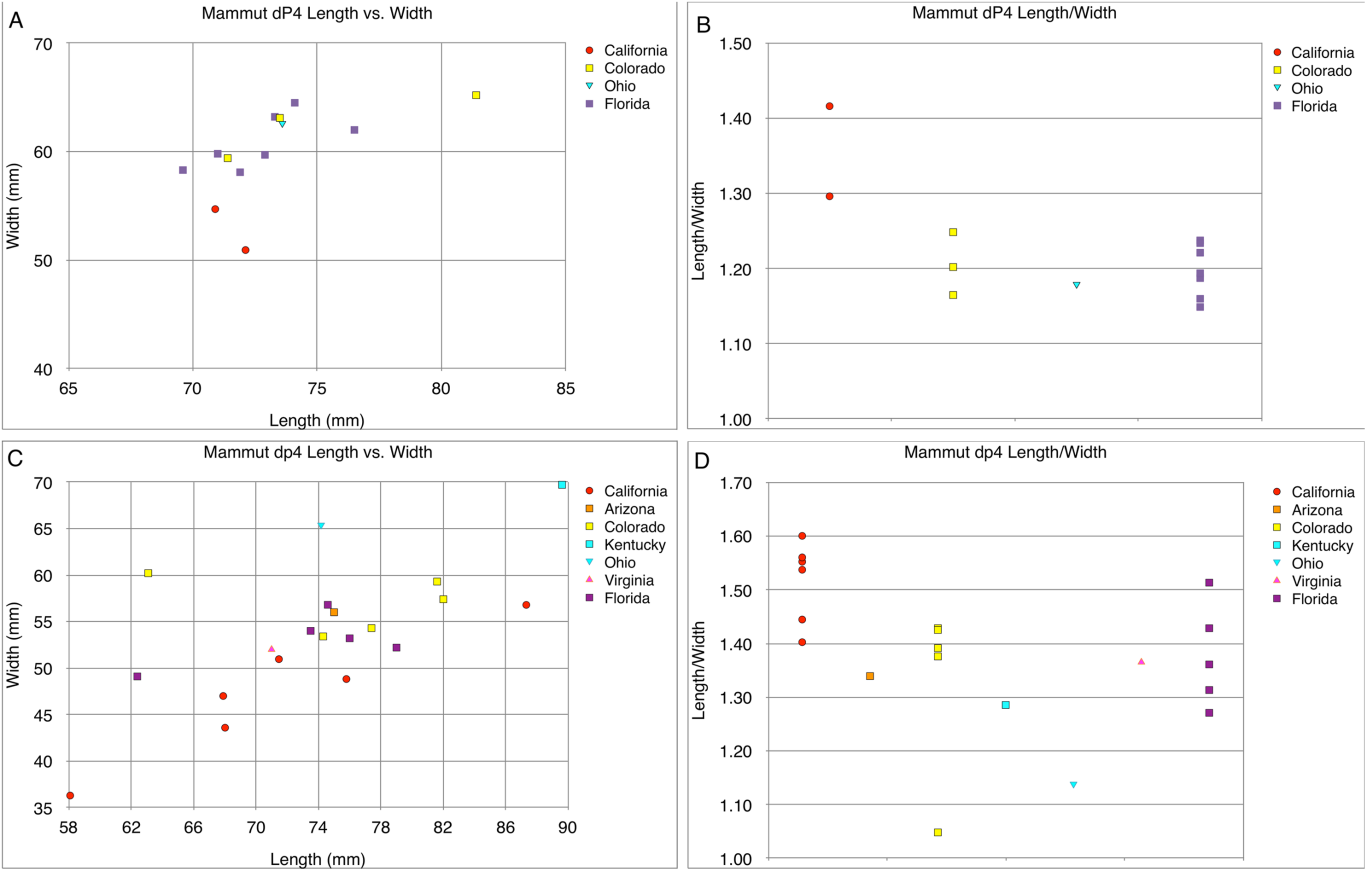
Length and width comparisons of *Mammut* dP4 and dp4, by state/province/country. (A) Length vs. width of *Mammut* dP4. *M. pacificus* is indicated in red, (B) length/width of *Mammut* dP4, organized by location. *M. pacificus* is indicated in red, (C) length vs. width of *Mammut* dp4. *M. pacificus* is indicated in red, and (D) length/width of *Mammut* dp4, organized by location. *M. pacificus* is indicated in red.

**Figure 30:**
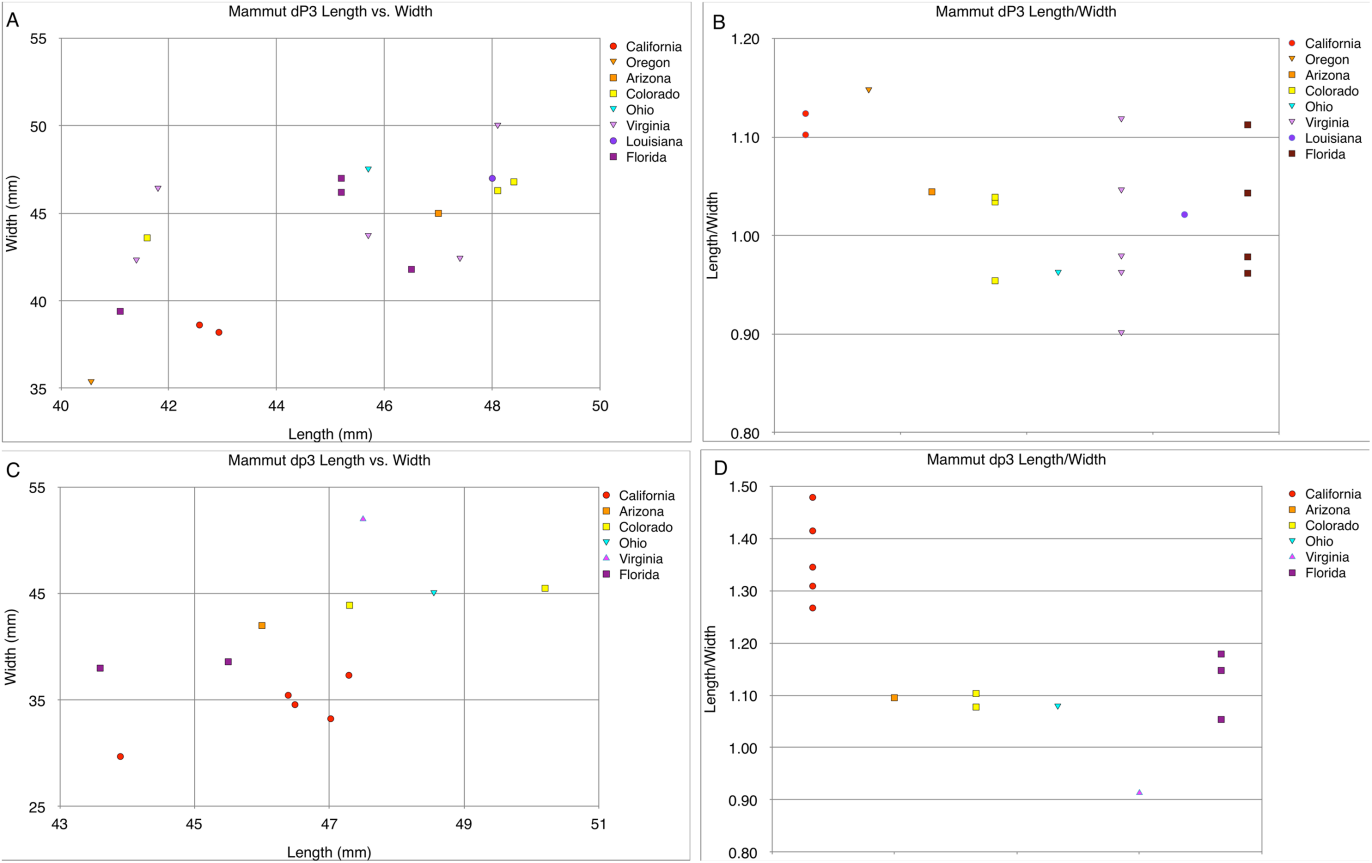
Length and width comparisons of *Mammut* dP3 and dP3, by state/province/country. (A) Length vs. width of *Mammut* dP3. *M. pacificus* is indicated in red, (B) length/width of *Mammut* dP3, organized by location. *M. pacificus* is indicated in red, (C) length vs. width of *Mammut* dP3. *M. pacificus* is indicated in red, and (D) length/width of *Mammut* dP3, organized by location. *M. pacificus* is indicated in red.

**Figure 31:**
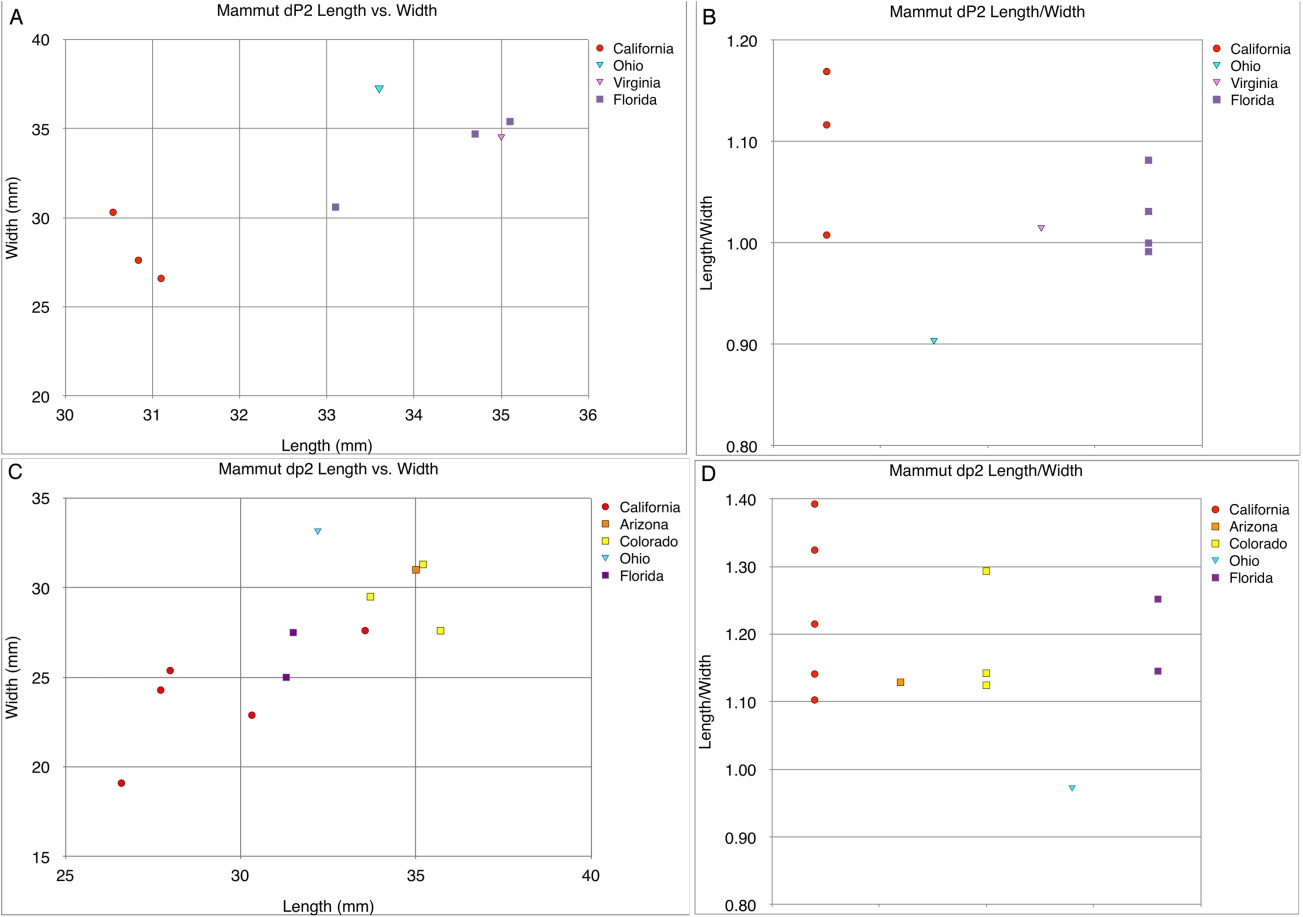
Length and width comparisons of *Mammut* dP2 and dp2, by state/province/country. (A) Length vs. width of *Mammut* dP2. *M. pacificus* is indicated in red, (B) length/width of *Mammut* dP2, organized by location. *M. pacificus* is indicated in red, (C) length vs. width of *Mammut* dp2. *M. pacificus* is indicated in red, and (D) length/width of *Mammut* dp2, organized by location. *M. pacificus* is indicated in red.

#### Vertebrae

WSC 18743 includes portions of seven vertebrae (Fig. 5), including the fifth cervical, two posterior thoracic, two lumbar, and two poorly preserved specimens that are probably additional posterior thoracics. Morphologically, these vertebrae do not differ in any substantial way from corresponding elements from *M. americanum*, and the key features indicated by Olsen (1972) and Hodgson et al. (2008a) as distinguishing *Mammut* from *Mammuthus* are all present. One of the lumbar vertebrae (Figs. 3G and 3H) is a close morphological match for the first lumbar of PRI 8829, a somewhat larger specimen of comparable ontogenetic age (LG XXI) (Hodgson et al., 2008a). Based on comparison to PRI 8829, both thoracic vertebrae (Figs. 3C–3F) appear to be posterior to the 13th thoracic (Hodgson et al., 2008a).

Several other specimens include larger numbers of preserved vertebrae, particularly among the Diamond Valley Lake sample. WSC 7561, tentatively identified as an adult male (based on an associated tusk) includes the first 11 vertebrae in articulation (Fig. 20). The first four thoracic vertebrae have long, slender, and posteriorly directed spinous processes, increasing in height from the first to the fourth vertebra, that are expanded dorsally, a feature also seen in *Mammut americanum*. SDSNH 86541 (Figs. 21D–21F) and WSC 13552 are adult individuals with several associated vertebrae, all of which show complete epiphyseal fusion. These two individuals have vertebrae that are substantially smaller than those in WSC 18743 or WSC 7561; these likely represent adult females (as mentioned above, SDSNH 86541 also includes tusks that suggest this individual was a female).

#### Sacrum and pelvis

WSC 18743 includes a nearly complete pelvic girdle (Fig. 4). The pelvic elements are fully fused, consistent with the maturity of the specimen based on dental eruption and wear (Haynes, 1991). The sacrum consists of six fused vertebrae. A second nearly complete pelvic girdle from Diamond Valley Lake, WSC 9642 (Fig. 22), represents a younger individual than WSC 18743, with only partial fusion of the pelvic elements. Five vertebrae are fully fused in WSC 9642, with the sixth vertebra partially fused and with expanded transverse processes that are consistent with the sixth sacral of WSC 18743. A third specimen from Riverside County, SBCM 5.3.298, is an adult (LG XXI, 36 ± 2 AEY) with six fully fused sacral vertebrae. In contrast, most adult *M. americanum* specimens seem to have only five sacral vertebrae, including AMNH 9951, NYSM VP 58, UF 5200, and USNM 8204. PRI 8829 includes five vertebrae in the sacrum, which is fused anteriorly to the third lumbar vertebra (Hodgson et al., 2008a). There is, however, some variation in the *M. americanum* sacral count. According to Woodman & Branstrator (2008), CMC VP-1 from Indiana has six sacral vertebrae. Haynes (1991) mentions a specimen from the University of Wisconsin (no catalog number provided) with only four sacrals. In *Mammuthus primigenius*, while there are typically five sacral vertebrae, there is some individual variation (Lister & Stuart, 2010).

Green (2006), Hodgson et al. (2008a), and Fisher (2008) found that the ratio of the pelvic aperture width to the minimum width of the ilial shaft is a reliable indicator of sex in *M. americanum*, a relationship that was first recognized in mammoths by Lister (1996). Females have a ratio of greater than 2.6, while in males it is less than 2.6. In WSC 18743, this ratio is 2.29, indicating it is a male specimen, consistent with its large overall size and robust tusks. Likewise, WSC 9642 is also apparently a male, with a ratio of 2.45.

#### Femur

The only preserved limb element from WSC 18743 is the distal end of the left femur (Fig. 5). The distal femoral epiphysis is fully fused to the shaft of the femur, which is consistent with the tooth wear state and vertebral epiphyseal fusion in indicating a fully mature animal. The femur is noteworthy for its size. The maximum width across the distal end is 288 mm, far larger than any other California specimen. There are, however, several specimens of *M. americanum* that are larger in this dimension. For example, DMNH 1496 from Indiana has a distal width of 299 mm with a length of 1,133 mm, while DMNH 61338 from Colorado has a distal width of 323 mm, with a length of 1,180 mm.

Several other femora of *M. pacificus* are known, in some cases with associated skeletal elements. One particularly noteworthy example is SDSNH 86541 (Fig. 21B), which includes most of a forelimb, numerous vertebrae, both tusks, and an m2 in addition to a complete femur. The femur exhibits complete fusion of both epiphyses indicating that it is from an adult, which is consistent with the wear on m2 and indicates LG XXII. Based on the presence of a small diameter tusk combined with the ontogenetic age, this specimen is interpreted as representing a female animal. Yet this femur has a distal width of only 203 mm, with a length of just 817 mm, remarkably small for an adult mastodon. Nor is this unique among specimens of *M. pacificus*. LACM-HC 1266 is not associated with any dental material, but it does exhibit complete epiphyseal fusion, with a distal width of 222 mm and a length of 820 mm. The nearest comparable *M. americanum* femur we have examined is G25656, a specimen from Carters Bog, Ohio, of an apparent young adult female (LG XII; 18 ± 1 AEY), with a distal femoral width of 220 mm and a length of 899 mm. WSC 9622, an apparent adult male *M. pacificus* (LG XX) with complete femoral epiphyseal fusion has a distal femoral width of 261 mm and length of 956 mm. This is only slightly larger than PRI 49618, an apparent adult female (LG XX–XXI; Hodgson et al., 2008b; Fisher, Beld & Rountrey, 2008) with a distal width of 240 mm and a length of 950 mm (Hodgson et al., 2008a). In fact, seven California femora (Fig. 23) from five different Rancholabrean localities spread across at least 35,000 years have an average maximum length of 918.8 mm, compared to the *M. americanum* average of 1,041.2 mm, based on nine Rancholabrean specimens, suggesting that femora of *M. pacificus* may have been shorter on average than those of *M. americanum*. A Mann–Whitney test on the length data showed a significant difference in the medians of the samples (*U*-value = 10.5, *p* = 0.050). In a slightly smaller subsample for which data are available (six *M. pacificus* and eight *M. americanum*), femora of *M. pacificus* femora tend to be more robust, with a length:midshaft diameter ratio of 6.16 compared to 6.75 in *M. americanum* (Mann–Whitney test *U*-value = 1, *p* < 0.001) (Fig. 25).

## Discussion

Trayler & Dundas (2009) first noted the possible difference in tooth proportions of California mastodons, comparing a small number of Rancho La Brea specimens to published records from Boney Spring and Trolinger Spring (Saunders, 1977). The much larger sample examined in this study confirms this trend and demonstrates that the California/Idaho population is unique in this regard. The average L:W ratio of m3s of *M. pacificus* (2.24, SD = 0.13) is nearly three standard deviations above the average of *M. americanum* (1.91, SD = 0.13), and for M3s the *M. pacificus* average (1.98, SD = 0.14) is just over two standard deviations higher (1.77, SD = 0.10) (Tables 4 and 5).

We considered other explanations than species-level separation for the disparity in tooth proportions between California/Idaho and non-California populations of *Mammut* but ultimately rejected each one. For example, one possibility is that the narrow tooth morphology was an outlier in the normal range of *Mammut americanum* morphology that became dominant when certain environmental conditions were met for which this morphology was advantageous. Indeed, even with the most extreme tooth positions (M3 and m3), there are a small number of specimens of *M. americanum* in which the L:W ratio approaches the mean of *M. pacificus* (none actually reach or exceed the mean of *M. pacificus*). However, these occurrences are infrequent and do not exhibit any clustering; each occurrence is found in an area in which the narrow tooth is an isolated outlier in a population more typical of *M. americanum*. Moreover, the narrow tooth morphology observed in *M. pacificus* is found over a large geographic area encompassing an area ranging from the Pacific coast to at least 1,000 km inland, and at least 1,200 km north–south. These localities include low-lying coastal areas, inland valleys, and mountainous areas in northern California and Idaho. Indeed, allowing for the spatially discontinuous nature of known deposits, the only parts of California that appear to have been completely devoid of *M. pacificus* (or, indeed, mammutids of any species) in the Pleistocene are the Sonoran and Mojave Deserts. It is unlikely that a critical environmental condition would occur in all of these areas yet not be found anywhere else in North America. Moreover, the narrow tooth morphology was present in this region over a lengthy temporal span, a period of at least 190–135 ka years and, based on the limited number of Irvingtonian specimens, likely considerably longer. Given this, we consider it unlikely that *Mammut pacificus* is a locally adapted morph of *M. americanum* and instead conclude that they in fact represent different taxa.

Another possibility that we considered and ultimately rejected is that the California/Idaho population arose via the isolation of an *M. americanum* subpopulation at a relatively late date. Such an isolated population of a widespread, morphologically diverse species can diverge through a Founder’s Effect (Mayr, 1942), leading to an anomalously high occurrence of characters that only appear as outliers in the parent population, even with a strong adaptive pressure. While there is some overlap in tooth proportions between *M. pacificus* and *M. americanum*, if the narrow tooth proportions of *M. pacificus* are in fact due to a Founder’s Effect in a recently isolated population, we would expect the size range variation to be less in *M. pacificus* than in *M. americanum*. In fact, we observe the opposite; the SD for length:width in *M. pacificus* is slightly greater than that in *M. americanum* (0.14–0.10 in M3, 0.13–0.11 in m3). Moreover, most California and Idaho teeth are not simply outliers within the range of *M. americanum* proportions, but in fact are completely outside the range of *M. americanum* specimens examined in this study. Finally, the small number of Irvingtonian specimens available suggest that this morphological pattern has been present for at least 190–135 ka, and perhaps much longer. Irvingtonian M3s/m3s from California and Idaho show proportions consistent with Rancholabrean specimens *M. pacificus*, while Irvingtonian specimens from Florida are similar to Rancholabrean specimens of *M. americanum*.

A third potential explanation for the disparity in tooth proportions continentwide is that the narrow-toothed, western populations of *Mammut* lie within the range of morphological variability expected for a genus as cosmopolitan and morphologically variable as *Mammut*. However, statistical comparisons between disparate *Mammut* populations suggest that *M. pacificus* is a consistent and significant statistical outlier. No other geographic subpopulation examined in this study differs so dramatically from *M. americanum* as do the California and Idaho populations (Table 16). *T*-test values for California M3 and m3 L:W are *p* < 0.001 when compared to non-California specimens. These *p*-values hold when comparing California to other individual states with large samples (*N* ≥ 9) and normal sample distributions, including Florida and Missouri (M3) and Colorado, Indiana, and Louisiana/Mississippi (m3). No other pairing of these states has such a low *p*-value, ranging from 0.031 (Indiana-Missouri m3) to 0.909 (Colorado-Louisiana/Mississippi m3). *Mammut americanum* maintains remarkably consistent L:W ratios over an enormous area, further undermining the hypothesis that the *M. pacificus* morphology is a regional variation of a single highly variable species, while bolstering the idea that midwestern and eastern specimens really do belong to a single taxon, *M. americanum*.

**Table 16:** *T*-Test results of tooth comparisons.

*M. pacificus*–*M. americanum* comparisons		State-to-state comparisons (*n* ≥ 9)			
Tooth position	*p*	Tooth position and state pair	*p*	Tooth position and state pair	*p*
M3	***p* < 0.001**	M3 CA–FL	***p* < 0.001**	M3 FL–MO	*p* = 0.685
m3	***p* < 0.001**	M3 CA–MO	***p* < 0.001**	m3 CO–IN	*p* = 0.416
M2	*p* = 0.213	m3 CA–CO	***p* < 0.001**	m3 CO–LA/MS	*p* = 0.909
m2	***p* < 0.001**	m3 CA–IN	***p* < 0.001**	m3 IN–LA/MS	*p* = 0.229
		m3 CA–LA/MS	***p* < 0.001**		

**Note:**

Columns 1 and 2 compare the *M. pacificus* to *M. americanum* L:W samples for each tooth position. Only M3, m3, M2, and m2 have large (*n* > 20) samples from each taxon. Note that there are significant differences at the 95% confidence level for M3, m3, and m2, but not for M2, indicating that the latter is not sufficient for differentiating these taxa. Columns 3 and 4 compare the state-level samples between California and other individual states in which *n* ≥ 9 and the sample was normally distributed (M3 from Florida and Missouri, and m3 from Colorado, Indiana, and Louisiana/Mississippi). Note that in every case the California sample was significantly different at the 95% confidence level. Columns 5 and 6 compare state-level samples of *M. americanum* from different states with normally-distributed samples in which *n* ≥ 9. In every case, the samples did not significantly differ at the 95% confidence level, indicating that *M. americanum* populations do not differ from each other in M3 and m3 L:W ratios. Bold = significant at 99%.

### Comparison of *Mammut pacificus* to other named species of *Mammut*

*Mammut oregonense* Hay, 1926, was based on an isolated left M2 (USNM 4911) of uncertain age from Baker County, Oregon. Measurements provided by Osborn (1936) include a length of 111 mm and a width of 80 mm. The length of this tooth is large compared to California specimens, and rather small compared to non-California specimens, while falling well within the range of each. The L/W ratio based on these measurements, 1.38, is quite high, and in fact higher than the average for either *M. pacificus* or *M. americanum*, but again, within the range of each (Figs. 27A and 27B). Since *M. pacificus* was present in both northern California and southern Idaho, its presence in Oregon would not be surprising. However, the limited numbers of specimens from Washington, Yukon, and Alaska are generally morphologically similar to *M. americanum* and are grouped as such in this study (Figs. 26B, 26D and 27D). Given the lack of diagnostic value of M2 (Table 6), *M. oregonense* is here regarded as a *nomen dubium*.

*Mammut furlongi* Shotwell & Russell 1963 is based upon a partial mandible with m1–m3 and a referred M3 from an assemblage dating to the Clarendonian NALMA in Oregon. While the mandibular symphysis is incomplete, there is no evidence of lower tusks. The teeth are small and quite narrow; the left m3 has an L:W ratio of 2.25, approximately the same as the average of *M. pacificus*. The m3 is fairly choerodont compared to both *M. pacificus* and *M. americanum*, but this feature tends to be quite variable. The referred M3 has only three lophs and a large talon at the posterior end. In this respect it is more similar to *Zygolophodon* cf. *proavus* from the Barstovian NALMA of California (Lofgren & Anand, 2011) than to *Mammut*; a detailed comparative study is needed to determine if *M. furlongi* should be referred to *Zygolophodon*.

*Mammut nevadanus* (Stock, 1936), originally placed in *Pliomastodon*, is based on a partial cranium with M2s, unerupted M3s, and the right tusk from the Thousand Creek beds of Humboldt County, Nevada. These beds are thought to be late Miocene to early Pliocene (Merriam, 1910) and the fauna dates to the Hemphillian NALMA (Prothero & Davis, 2008). This specimen has relatively small M3s that have four complete lophs rather than three as in *M. furlongi*. The M3s are slightly more narrow than the average for *M. pacificus* (L:W = 2.03), but well within the *M. pacificus* range, and more narrow than any *M. americanum* specimen. The single, slender tusk is straight and deflected ventrally in lateral view and curved gently medially in ventral view. This differs from both *M. pacificus* and *M. americanum,* which have large, upturned tusks in males, and upturned or straight but anteriorly directed tusks in females.

*Mammut raki* (Frick, 1933) is based on a partial mandible from Blancan NALMA deposits in Sierra County, New Mexico (Lucas & Morgan, 1999). This specimen has a narrow m3 in which the L:W ratio (2.30) is within the range of but greater than the average for *M. pacificus* (2.25). The holotype specimen of *M. raki* also possesses well-developed mandibular tusks, distinguishing it from *M. pacificus*. Rancholabrean *Mammut* from New Mexico have tooth proportions that generally fall within the range of *M. americanum* rather than *M. pacificus* (average m3 L:W = 1.87, *n* = 3).

*Mammut cosoensis* (Schultz, 1937) is based on a holotype partial cranium and a referred partial mandible, juvenile palate, and isolated teeth from the Coso Formation in Inyo County, California. The Coso Formation is mid to late Pliocene based on K–Ar dating of sanidine, biotite, and plagioclase as well as whole-rock samples of igneous rocks bracketing and intruding the Coso Formation sediments (Bacon et al., 1982), with a fauna that is probably Blancan. Schultz (1937) originally referred this species to *Pliomastodon*? as he considered it the “most advanced species of *Pliomastodon* yet described” (p.105). Shoshani & Tassy (1996) referred this species, and in fact the entire genus *Pliomastodon*, to *Mammut*.

The holotype cranium of *M. cosoensis* (LACM 1719) includes the upper second and third molars, and is approximately LG XXII, 39 ± 2 AEY. The proximal ends of the tusks are preserved, and are upturned and appear to have been rather long, with a preserved length of approximately 740 mm and a basal diameter of 78 mm. In comparison, SDSNH 86541, a presumed female *M. pacificus* also has a basal diameter of 78 mm, with a complete tusk length of only 780 mm, while the holotype *M. pacificus* (WSC 18743, a male) has a basal tusk diameter of 186 mm, with a length of 1,996 mm. Both WSC 18743 and SDSNH 86541 are also LG XXII. But while the tusk morphology of LACM 1719 is consistent with a female, the premaxilla morphology is more similar to a male specimen. The ventral edges of the tusks lie just dorsal to the tooth rows in lateral view, similar to adult male specimens of *M. americanum* and *M. pacificus*. It appears that the vertical separation between the tusks and the upper teeth is lost in adult males as they reach their maximum tusk diameter, but in adult females with their smaller tusks the spacing is maintained (e.g., in *M. americanum* AMNH 14292 as figured in Osborn (1936)). It is also noteworthy that in WSC 18743, a male in the same Laws Group with large tusks, there is still a substantial gap between the tusk and tooth row. Whether LACM 1719 represents a male or a female, it differed in some degree from *M. americanum* and *M. pacificus* in cranial geometry associated with tusk growth. While Schultz (1937) stated that *M. cosoensis* referred mandible LACM 1720 showed no indication of lower tusks, examination of both Schultz’s figures and the specimen itself show that the mandible, including the symphysis, is largely reconstructed with plaster, making the status of lower tusks unclear.

Both the upper and lower third molars of *M. cosoensis* have an enamel structure at the anterolabial corner that appears to be a hyper-enlarged cingulum. This is most strongly developed in LACM 1719 and LACM 1720, which show wear on the occlusal surface in spite of the relatively early wear on these teeth, but is only weakly developed in referred M3 LACM 2015. No specimen of *M. pacificus* or *M. americanum* examined in this study exhibits such strong development of the anterior cingulum.

While the sample size is small, the L:W ratios of *M. cosoensis* are uniformly high, and close to the averages for *M. pacificus*. Two M3s have L:W ratios near the middle of the range of *M. pacificus* (2.10, 1.94), and one of these is outside the range for *M. americanum*. The M3s have fives lophs, which is common (but not universal) in *M. pacificus* and rare in *M. americanum*. The two known m3s have L:W ratios in the lower part of the range for *M. pacificus*, and the upper part of the range for *M. americanum* (2.21, 2.02). In almost every instance, the actual length and width measurements are smaller than the average for *M. pacificus*, and in most cases substantially so. The only known examples of DP2, DP3, and DP4 (all from one individual, LACM2036) are far smaller than any known specimen of *M. pacificus* or *M. americanum*, while the known examples of M2 and M3 are smaller than almost any known specimens of *M. pacificus*. The single outlier is the referred mandible, LACM 1720, in which both the m2 and m3 are quite wide, and the m3 is nearly as long as the average for *M. pacificus*.

The relationships among *M. pacificus*, *M. furlongi*, *M. nevadanus*, *M. cosoensis*, and *M. raki* are unclear. Assuming the M3 referred to *M. furlongi* is correctly attributed to this taxon, *M. furlongi* may have affinities with *Zygolophodon*. The slender, straight, ventrally deflected tusks distinguish *M. nevadanus* from *M. pacificus*. Likewise, the presence of lower tusks in *M. raki* separate it from *M. pacificus*, but in the absence of good mandibular material in *M. cosoensis* it is difficult to compare it directly to *M. raki* in particular. It appears that *M. cosoensis* may have had smaller teeth on average than either *M. pacificus* or *M. raki* (acknowledging that *n* = 1 for *M. raki)*, Moreover, the unusual premaxillary morphology in the holotype of *M. cosoensis* suggests a somewhat different growth trajectory than in *M. pacificus*, perhaps with adult males lacking the hyper-enlarged tusks observed in both *M. pacificus* and *M. americanum*. The Clarendonian occurrence of *M. furlongi*, Hemphillian occurrence of *M. nevadanus*, and Blancan occurrences of *M. cosoensis* and *M. raki*, all with narrow m3s in western states, has interesting implications for the evolutionary timing and geographic distribution of narrow-toothed mastodonts.

*Mammut raki* and *Mammut cosoensis* represent narrow-toothed mammutid taxa from Blancan deposits in New Mexico and California, respectively. *Mammut nevadanus* from the Hemphillian of Nevada, and *Mammut furlongi* from the Clarendonian of Oregon are also narrow-toothed forms. While all four of these taxa are based on limited material that makes comparison with other taxa difficult, if the narrow-toothed taxa are indeed monophyletic they suggest an early divergence of eastern and western mammutids and a wider western distribution of narrow-toothed mammutids in the Miocene/Pliocene.

*Mammut pacificus* is not known from Inyo County, California (the only known locality of *M. cosoensis*), and Pleistocene specimens of *Mammut* from New Mexico have broad teeth similar to *M. americanum* rather than narrow teeth like *M. raki*.

**Figure 32:**
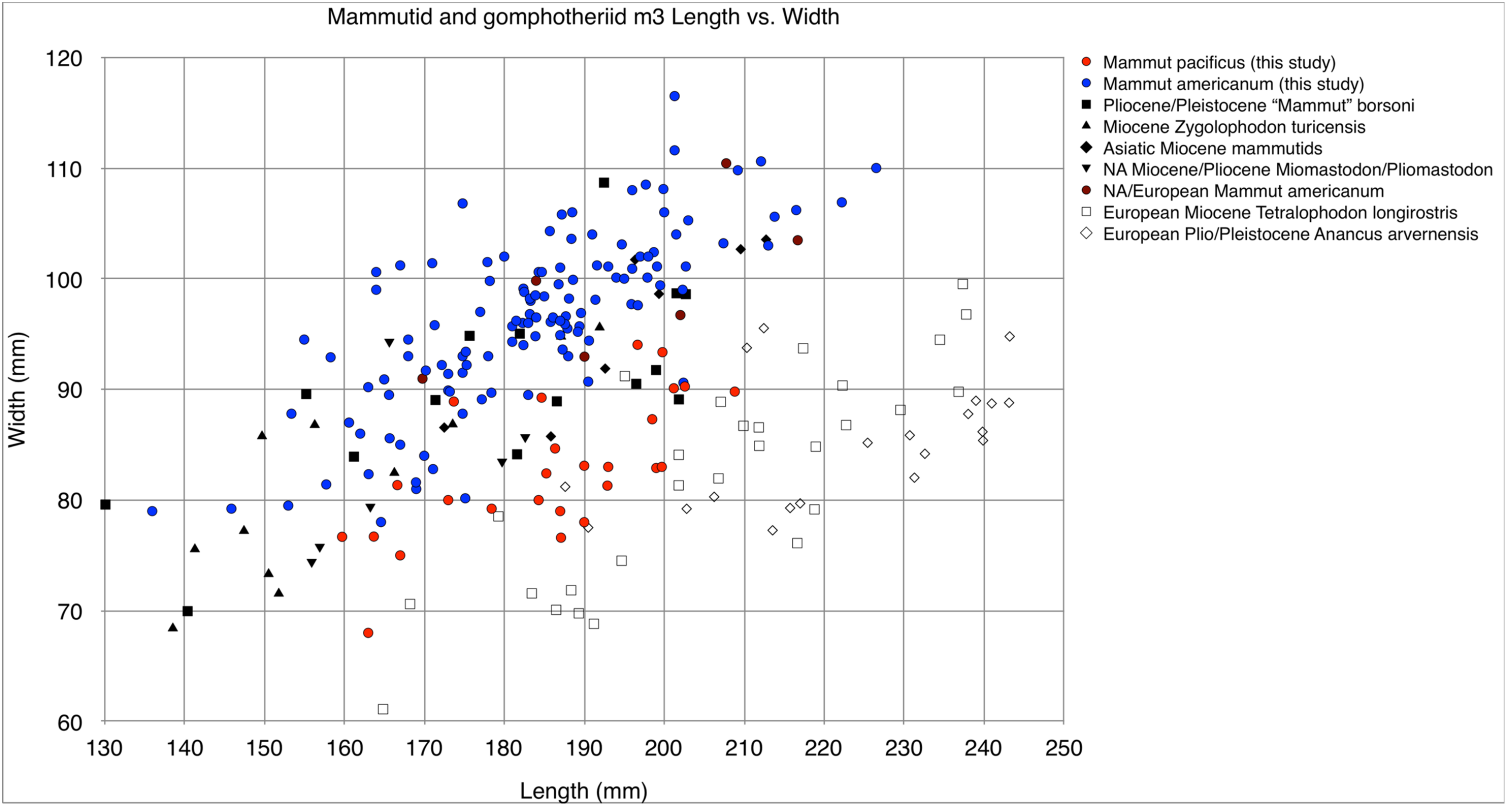
Comparison of m3 proportions of mammutids and gomphotheriids. Graph of length vs. width of mammutids and gomphotheriids, modified from Tobien (1996). Closed symbols represent mammutids, open symbols represent gomphotheriids. Blue symbols are *M. americanum* from this study, while red symbols are *M. pacificus*; other points are from Tobien (1996). Note that *M. pacificus* is intermediate between other mammutids and gomphotheriids in m3 narrowness.

Tobien (1996) showed that over time mammutid m3 length:width ratios remain relatively constant, but mammutid m3s are distinctly wider than the m3s of gomphotheriids. The m3 L:W ratios of *Mammut pacificus* are intermediate between those of gomphotheriids and other mammutids, including Asiatic specimens (Fig. 32). If the apparent scarcity of narrow-toothed mammutids in Eurasia suggested by Tobien’s (1996) data is an accurate reflection of the Eurasian fauna, they suggest that this morphology first evolved in North America. The presence of multiple lineages of North American mammutids should be taken into account during the much-needed reassessment of North American mammutids including *Zygolophodon*, *Miomastodon*, and “*Pliomastodon.*”

### Biogeography

Like most other animals, proboscidean morphotypes tend to cluster in distinct geographic areas. The most extreme examples are various insular dwarf species of elephantids and stegodontids isolated on islands (Van Der Greer et al., 2016), but even widely dispersed mainland taxa tend to have distinct ranges (Roca et al., 2001; Fisher, 2009). In North America, perhaps the best-known example among proboscideans is the northern distribution of *Mammuthus primigenius*, which contrasts with the more southern range of *Mammuthus columbi*. Recent studies have suggested that there was extensive hybridization between these species (Enk et al., 2011, 2016). Indeed, it seems that among the Elephantidae, interspecific breeding is possible and perhaps relatively commonplace (Roca et al., 2001; Palkopoulou et al., 2018). Yet regardless of apparently frequent interbreeding, proboscidean populations seem to have for the most part remained morphologically stable and distinctive. While much work remains to be done on mastodon ecology, research to date suggests that *Mammut* may have had fairly stringent ecological preferences in terms of water availability and vegetation when compared to elephantids (Jackson & Whitehead, 1986; McAndrews & Jackson, 1988; Whitehead et al., 1982), and so it is perhaps not surprising that *Mammut* could diverge morphologically in geographically isolated areas even if mammutids were capable of the relatively high mobility observed in some other proboscideans.

*Mammut* specimens from the Pacific Northwest present an interesting anomaly. There are very few Pleistocene mammutid remains from Oregon, and no easily identified elements such as third molars. In this study, Oregon specimens have been included as *M. americanum*, but it is possible that these remains represent *M. pacificus*, especially given the proximity of *M. pacificus* in northern California.

There are small numbers of *Mammut* known from Washington, Alaska, and the Yukon. While teeth from these specimens are quite small, the L:W ratios fall within the expected range for *M. americanum* and are included as such here. The specimens from Alaska and the Yukon all appear to be no younger than the early Rancholabrean, and it seems that mammutids left this region prior to the glacial maximum (Zazula et al., 2014). All three referred specimens of *M. pacificus* from Idaho also date from prior to the last glacial maximum. The Pacific Northwest may have represented a conspecific region for both taxa, or possibly the ranges fluctuated with each taxon occupying the region at different times. A larger sample and better chronology will be necessary to determine the exact nature of northwestern mammutid interactions.

The unexpected concentration of *Mammut americanum* at Ziegler Reservoir in Colorado is located more than 1,300 km east of the Pacific Coast, and over 600 km southeast of the nearest occurrence of *M. pacificus* in Idaho. In the vast region between Ziegler Reservoir and the easternmost occurrences of *M. pacificus*, there are only a handful of known specimens of *Mammut*. The genus is relatively rare throughout Arizona, New Mexico, Utah, Nevada, Wyoming, and Montana, with only a few if any reported specimens from each state. As in Colorado, the only records from Utah are from high elevations (Miller, 1987). This stands in stark contrast to the common prevalence of other Plio-Pleistocene proboscideans in the American Southwest and Mexico, including *Mammuthus* and gomphotheriids such as *Cuvieronius*, *Rhynchotherium,* and *Stegomastodon*. McDonald et al. (2010) suggested that limited, elevation-controlled distribution of coniferous forests in the Rocky Mountains may have limited the size of mastodon populations in this region; this may have been true of the basin-and-range topography as well.

**Figure 33:**
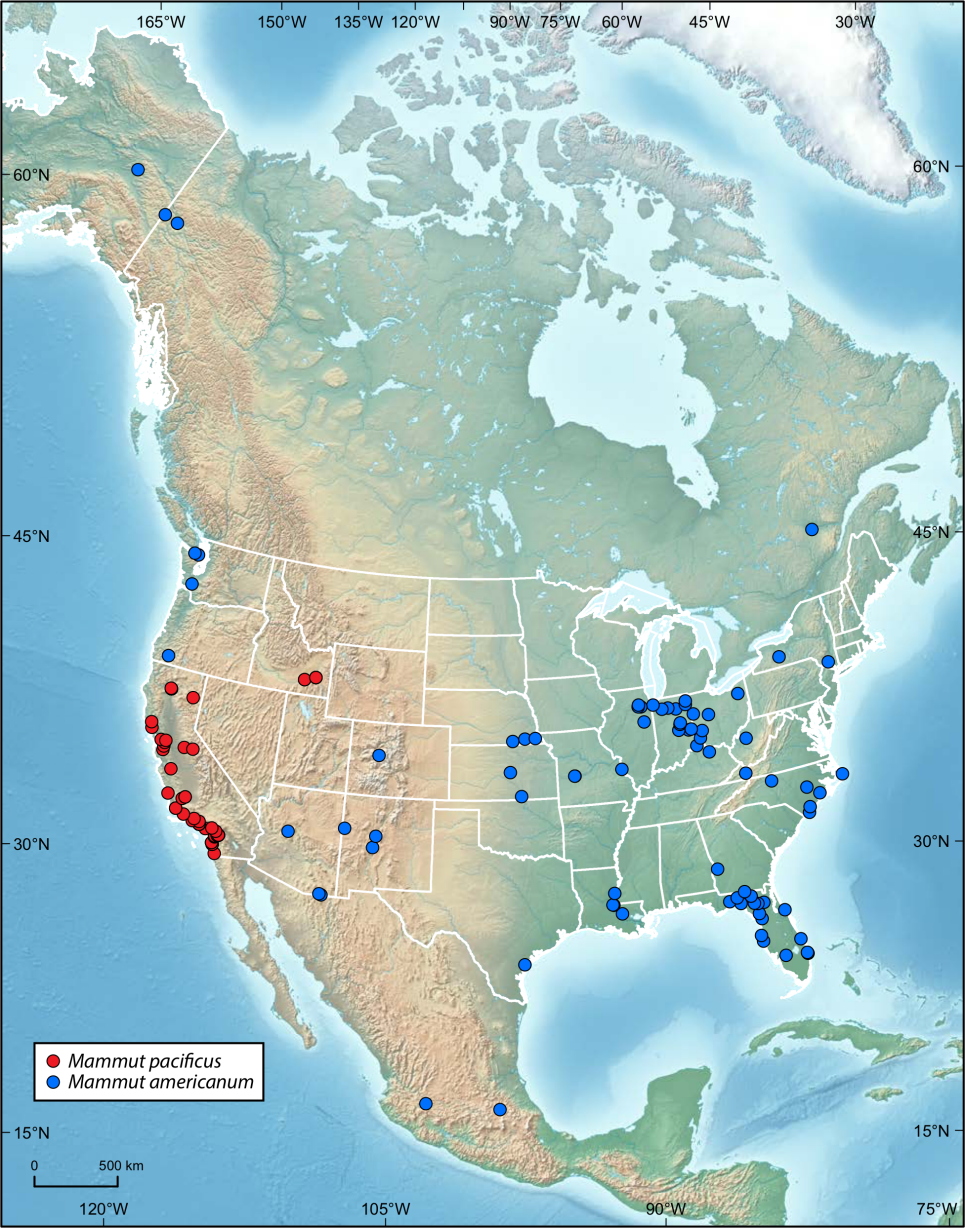
Map of North America showing the distribution of *Mammut pacificus* and *Mammut americanum* from this study. Red circles mark all known *M. pacificus* localities, while blue circles mark the *M. americanum* localities that produced teeth used in this study and represented in Table S2. Note that while there are many additional *M. americanum* localities that were not included in this study and that are not indicated on the map, there are no known *M. americanum* localities in California. The *M. americanum* locality in Oregon is a non-diagnostic specimen that was included as *M. americanum* in this study, but that could represent *M. pacificus*.

*Mammut pacificus* was widespread in California west of the Sierra Nevada, and (at least in the Irvingtonian and early Rancholabrean) was present as far northeast as southern Idaho, but it was apparently absent from the Sonoran and Mojave Deserts (Fig. 33), including from heavily sampled localities such as Tule Springs in Nevada (Scott, Springer & Sagebiel, 2017). These deserts, along with the high, steep, and at times glaciated Sierra Nevada and the possible patchiness of appropriate habitats in the Basin and Range and Rocky Mountains, may have served as effective geographic barriers to mastodon dispersal. It is interesting to note that there are some indications of similar distribution patterns in *Mammuthus*. Widga et al. (2017) found that *Mammuthus exilis* from the California Channel Islands and southern California specimens of *M. columbi* had dental characteristics in common with each other, but that these two populations differed from *M. primigenius*, *M. jeffersonii*, *M. trogontherii*, and all the other regional populations of *M. columbi* in their dataset.

The sparse distribution of high growing, generally woody vegetation in desert environments may have limited the abundance of browsing specialists such as *Mammut* (but see Green, DeSantis & Smith, 2017; Smith & DeSantis, 2018), while xeric shrubs, succulents and occasional grasslands could have supported generalists such as *Mammuthus* and *Cuvieronius*. The limited abundance of high-growing woody vegetation in the high mountains and deserts of Mexico may also explain the limited reports of *Mammut* remains from Central America (Polaco et al., 2001) and the inability for *Mammut* to have traveled into South America via the Panamanian Land Bridge as did at least two species of gomphotheres (Mothé et al., 2017). Thus, while *Mammut* was a prominent member of the Pleistocene fauna in the eastern US, with *Mammut americanum* present from the Rocky Mountains east to the Atlantic Coast, it seems that there also existed a large section of North America where there were few if any mastodons, between *Mammut americanum* in the east and *Mammut pacificus* from southern Idaho and the Sierra Nevada west to the Pacific Coast.

## Conclusions

This study describes and formally names a new species of mastodon from the Pleistocene of western North America, *Mammut pacificus* sp. nov. This new taxon is recognized by specimens throughout California and from two localities in southern Idaho. With this effort, we have documented the presence of *two* species of *Mammut* in Pleistocene North America. The newly described *Mammut pacificus*, characterized by narrow molars, six sacral vertebrae, a femur with a proportionally greater mid-shaft diameter, and a lack of mandibular tusks, is recognized from the Sierra Nevada west to the Pacific Coast and into southern Idaho. The long-recognized *Mammut americanum*, characterized by wide molars, five sacral vertebrae, a femur with a proportionally small mid-shaft diameter, and occasional mandibular tusks, is known from Alaska south through the Rocky Mountains, and east to the Atlantic Coast. There is some degree of morphological overlap between the two species of *Mammut*, including sparse reports of other *Mammut* material from the western US that have led to questionable specific assignments. We suggest the need for a continental-wide reassessment of the evolution, biogeography, and phylogenetics of the Mammutidae family to elucidate these relationships.

## Supplemental Information

10.7717/peerj.6614/supp-1 Supplemental Information 1 *Mammut pacificus* tooth measurements. Measurements (in mm) of *Mammut pacificus* specimens examined in this study.

10.7717/peerj.6614/supp-2 Supplemental Information 2 *Mammut americanum* tooth measurements. Measurements (in mm) of *Mammut americanum* specimens examined in this study.

## Institutional Abbreviations

AMNH: American Museum of Natural History, New York, New York
BYUVP: Brigham Young University Museum of Paleontology, Provo, Utah
CMC VP: Cincinnati Museum Center, Cincinnati, Ohio
CPI: Coastal Plains Institute and Land Conservancy, Tallahassee, Florida
DMAS: Daytona Museum of Arts and Science, Daytona, Florida
DMNH: Denver Museum of Nature and Science, Denver, Colorado
ETMNH: East Tennessee Museum of Natural History, Gray, Tennessee
LACM: Natural History Museum of Los Angeles County, Los Angeles, California
G: Boonshoft Museum of Discovery, Dayton, Ohio
IMNH: Idaho Museum of Natural History, Pocatello, Idaho
IPFW: Indiana University Perdue University Fort Wayne, Fort Wayne, Indiana
JMM: Joseph Moore Museum, Richmond, Indiana
LACM-HC: Hancock Collection, La Brea Tar Pits Museum, Los Angeles, California
LC: Ralph B. Clark Interpretive Center, Buena Park, California
LSUMG: Louisiana State University Museum of Natural Science, Baton Rouge, Louisiana
NCSM: North Carolina Museum of Natural Sciences, Raleigh, North Carolina
NMMNH: New Mexico Museum of Natural History, Albuquerque, New Mexico
TMM: Texas Memorial Museum, Austin, Texas
NYSM VP: Vertebrate Paleontology, New York State Museum, Albany, New York
PRI: Paleontological Research Institute, Ithaca, New York
SBCM: San Bernardino County Museum, Redlands, California
SBMNH-VP: Santa Barbara Museum of Natural History, Santa Barbara, California
SDSNH: San Diego Natural History Museum, San Diego, California
UCMP: Museum of Paleontology, University of California-Berkeley, Berkeley, California
UF DMAS: Florida Museum of Natural History, Gainesville, Florida
UMMP: University of Michigan Museum of Paleontology, Ann Arbor, Michigan
USNM: United States National Museum of Natural History, Washington, DC
UWBM: University of Washington Burke Museum, Seattle, Washington
VMNH: Virginia Museum of Natural History, Martinsville, Virginia
WSC: Western Science Center, Hemet, California
YG: Yukon Government, Yukon Territory, Canada
YPM: Yale Peabody Museum, New Haven, Connecticut.

## Additional Information and Declarations

### New Species Registration

The following information was supplied regarding the registration of a newly described species: Publication LSID: urn:lsid:zoobank.org:pub:21ED43A5-6102-4D44-80C1-7D7A3D4651EA. Mammut pacificus LSID: urn:lsid:zoobank.org:act:BE79F9B4-1D49-415D-A250-6FB951CF81F6.

### Data Availability

The following information was supplied regarding data availability: Maximum length and width measurements of individual teeth are available in Files S1 and S2. Remains of Mammut from California: https://www.morphosource.org/Detail/ProjectDetail/Show/project_id/687.
