# Supplementary material for: Mammut pacificus sp. nov., a newly recognized species of mastodon from the Pleistocene of western North America
Source: PeerJ. 2019 Mar 27;7:e6614. doi: 10.7717/peerj.6614 (PMC6441323; doi:10.7717/peerj.6614)
Supplement: Supplemental Information 1 — Measurements (in mm) of Mammut pacificus specimens examined in this study. [file peerj-07-6614-s001.docx]

| **Rancholabrean specimens referred to *M. pacificus* included in this study** | | | | | | | | |
| --- | --- | --- | --- | --- | --- | --- | --- | --- |
| **Specimen** | **Side** | **Element** | **Length** | **Width** | **Locality** | **County** | **State** | **Reference** |
| **WSC 18743** | Right | M3 | 167.5 | 84.1 | Diamond Valley Lake, West Dam | Riverside | CA |  |
| **WSC 8817** | Left | M3 | 160.1 | 86.5 | Diamond Valley Lake, West Dam | Riverside | CA |  |
| **WSC 9622** | Left | M3 | 152.9 | 77.7 | Diamond Valley Lake, East Dam | Riverside | CA |  |
| **WSC 8904** | Right | M3 | 179.8 | 95.5 | Diamond Valley Lake, East Dam North | Riverside | CA |  |
| **WSC 10819** | Right | M3 | 197 | 88 | Diamond Valley Lake, East Dam St. John’s Channel | Riverside | CA |  |
| **WSC 10646** | Right | M3 | 176 | 84 | Diamond Valley Lake, East Dam Pond | Riverside | CA |  |
| **WSC 9964** | Left | M3 | 146 | 75.4 | Diamond Valley Lake, West Dam | Riverside | CA |  |
| **WSC 22300** | Left | M3 | 168 | 82 | Diamond Valley Lake, West Dam | Riverside | CA |  |
| **WSC 19730** | Right | M3 | 151.6 | 89.5 | Diamond Valley Lake, West Dam, San Diego Canal | Riverside | CA |  |
| **WSC 7387** | Right | M3 | 158.4 | 82.5 | Diamond Valley Lake, East Dam | Riverside | CA |  |
| **WSC 10829** | Left | M3 | 162.2 | 85.2 | Diamond Valley Lake, West Dam | Riverside | CA |  |
| **WSC 22587** | Left | M3 | 155.2 | 86.8 | Diamond Valley Lake, West Dam, San Diego Canal | Riverside | CA |  |
| **LC 0260** | Right | M3 | 190 | 87 | Fullerton | Orange | CA |  |
| **LACM-HC 1258** | Right | M3 | 150 | 78 | Rancho La Brea | Los Angeles | CA |  |
| **LACM-HC 87129** | Left | M3 | 155 | 83 | Rancho La Brea | Los Angeles | CA |  |
| **LACM-HC 87067** | Right | M3 | 172 | 84 | Rancho La Brea | Los Angeles | CA |  |
| **LACM-HC 87133** | Left | M3 | 172 | 81 | Rancho La Brea | Los Angeles | CA |  |
| **LACM-HC 60326** | Right | M3 | 158 | 83 | Rancho La Brea | Los Angeles | CA |  |
| **LACM-HC 87077** | Right | M3 | 179 | 93 | Rancho La Brea | Los Angeles | CA |  |
| **LACM-HC 87078** | Left | M3 | 175 | 90 | Rancho La Brea | Los Angeles | CA |  |
| **LACM-HC 87071** | Left | M3 | 168 | 81 | Rancho La Brea | Los Angeles | CA |  |
| **LACM-HC 87094** | Left | M3 | 175 | 90 | Rancho La Brea | Los Angeles | CA |  |
| **LACM-HC 87092** | Right | M3 | 172.57 | 90.54 | Rancho La Brea | Los Angeles | CA |  |
| **UCMP 212936** | Right | M3 | 188 | 95.5 | Calaveras Dam | Alameda | CA |  |
| **UCMP 36684** | Left | M3 | 163.84 | 77.91 | Doolan Canyon | Alameda | CA |  |
| **UCMP 45265** | Left | M3 | 182.85 | 89.27 | Union Oil Tank Farm | Contra Costa | CA |  |
| **UCMP 114599** | Right | M3 | 181.79 | 81.96 | Homestake Mine Road 1 | Lassen | CA |  |
| **UCMP 41642** | Left | M3 | 166.72 | 90 | 5 Oaks Ranch | Sonoma | CA |  |
| **UCMP 70139** | Right | M3 | 168.69 | 86.14 | Duckers Ranch, Petaluma | Sonoma | CA |  |
| **UCMP 1060** | Right | M3 | 142.9 | 78.13 | Gold Springs Gulch | Tuolumne | CA |  |
| **LACM 3014** | Right | M3 | 167.31 | 83.33 | Sun Valley | Los Angeles | CA |  |
| **LACM 149514** | Right | M3 | 202.77 | 86.84 | Simi Valley 7455 | Ventura Co. | CA |  |
| **WSC 6523** | Right | M3 | 157.51 | 87.56 | Diamond Valley Lake, West Dam | Riverside | CA |  |
| **SBMNH IP A-115** | Right | M3 | 183.89 | 91.02 |  | Santa Barbara | CA |  |
| **SBMNH Top drawer A** | Left | M3 | 189.46 | 104.26 |  | Santa Barbara | CA |  |
| **SBMNH 2nd drawer B** | Left | M3 | 187.14 | 85.97 |  | Santa Barbara | CA |  |
| **SDSNH 116399** | Right | M3 | 160.69 | 84.46 | Robertson Ranch, East Village (6044) | San Diego | CA |  |
| **SBCM 5.3.298** | Left | M3 | 170.8 | 87.0 | Perris | Riverside | CA |  |
| **LACM-HC 87076** | Left | M3 | 142.5 | 73.08 | Rancho La Brea | Los Angeles | CA |  |
| **UCMP 1567** | Right | m3 | 158.7 | 80.04 | Kincaide Flat | Tuolumne | CA |  |
| **WSC 18743** | Right | m3 | 187.1 | 76.6 | Diamond Valley Lake, West Dam | Riverside | CA |  |
| **WSC 8817** | Left | m3 | 184.3 | 80 | Diamond Valley Lake, West Dam | Riverside | CA |  |
| **WSC 19730** | Left | m3 | 173.7 | 88.9 | Diamond Valley Lake, West Dam, San Diego Canal | Riverside | CA |  |
| **LC 0260** | Right | m3 | 199 | 82.9 | Fullerton | Orange | CA |  |
| **UCMP 198852** | Right | m3 | 199.7 | 83 | Rancho La Brea | Los Angeles | CA | Trayler and Dundas 2009 |
| **UCMP 198853** | Left | m3 | 190 | 83.1 | Rancho La Brea | Los Angeles | CA | Trayler and Dundas 2009 |
| **LACM-HC 87326** | Right | m3 | 190 | 78 | Rancho La Brea | Los Angeles | CA |  |
| **LACM-HC 77** | Left | m3 | 187 | 79 | Rancho La Brea | Los Angeles | CA |  |
| **LACM-HC 87068** | Right | m3 | 167 | 75 | Rancho La Brea | Los Angeles | CA |  |
| **LACM-HC 87072** | Left | m3 | 193 | 83 | Rancho La Brea | Los Angeles | CA |  |
| **LACM-HC 87073** | Left | m3 | 163 | 68 | Rancho La Brea | Los Angeles | CA |  |
| **LACM-HC 87069** | Left | m3 | 173 | 80 | Rancho La Brea | Los Angeles | CA |  |
| **UCMP 1569** | Left | m3 | 198.5 | 87.3 | Mormon Ranch | Tuolumne | CA |  |
| **LACM 130515** | Right | m3 | 178.43 | 79.22 | Churro Creek Site | San Luis Obispo | CA |  |
| **LACM 44706** | Right | m3 | 185.26 | 82.41 | Outfill Sewer, Rodeo Kelley | Los Angeles | CA |  |
| **LACM 125982** | Right | m3 | 196.67 | 94.03 | Chandler's Sand Pit | San Pedro | CA |  |
| **LACM 149514** | Right | m3 | 208.82 | 89.8 | Simi Valley 7455 | Ventura Co. | CA |  |
| **LACM 152669** | Left | m3 | 159.74 | 76.68 | Lakes at Thousand Oaks | Ventura | CA |  |
| **SBMNH-IP A-115** | Left | m3 | 199.78 | 93.36 |  | Santa Barbara | CA |  |
| **SBMNH-VP 3342** | Left | m3 | 202.58 | 90.26 |  | Santa Barbara | CA |  |
| **SBMNH-VP 3341** | Left | m3 | 184.67 | 89.25 |  | Santa Barbara | CA |  |
| **SDSNH 116399** | Right | m3 | 186.36 | 84.66 | Robertson Ranch, East Village (6044) | San Diego | CA |  |
| **SBMNH-VP 3343** | Right | m3 | 166.64 | 81.35 | Carpinteria | Santa Barbara | CA |  |
| **USNM 13701** | Right | m3 | 163.7 | 76.7 | American Falls | Power | ID |  |
| **WSC 9622** | Right | M2 | 94.4 | 74 | Diamond Valley Lake, East Dam | Riverside | CA |  |
| **WSC 11634** | Left | M2 | 92.1 | 65.4 | Diamond Valley Lake, West Dam | Riverside | CA |  |
| **WSC 23419** | Left | M2 | 100.6 | 83.9 | Diamond Valley Lake, East Dam | Riverside | CA |  |
| **WSC 19730** | Right | M2 | 106.8 | 84 | Diamond Valley Lake, West Dam, San Diego Canal | Riverside | CA |  |
| **WSC 10844** | Right | M2 | 114.5 | 86.3 | Diamond Valley Lake, West Dam | Riverside | CA |  |
| **WSC 10829** | Right | M2 | 109.3 | 77.5 | Diamond Valley Lake, West Dam | Riverside | CA |  |
| **WSC 22587** | Left | M2 | 92.7 | 85.1 | Diamond Valley Lake, West Dam, San Diego Canal | Riverside | CA |  |
| **WSC 18743** | Right | M2 | 96.3 | 83.2 | Diamond Valley Lake, West Dam | Riverside | CA |  |
| **LC uncataloged** | Right | M2 | 102 | 84 | Orange Co. | Orange | CA |  |
| **UCMP 198860** | Right | M2 | 97.2 | 74.9 | Rancho La Brea | Los Angeles | CA |  |
| **LACM-HC 1258** | Right | M2 | 103 | 76 | Rancho La Brea | Los Angeles | CA |  |
| **LACM-HC 87129** | Left | M2 | 103 | 77 | Rancho La Brea | Los Angeles | CA |  |
| **LACM-HC 08084** | Right | M2 | 103 | 79 | Rancho La Brea | Los Angeles | CA |  |
| **LACM-HC 87133** | Left | M2 | 101 | 78 | Rancho La Brea | Los Angeles | CA |  |
| **LACM-HC 87096** | Left | M2 | 105 | 79 | Rancho La Brea | Los Angeles | CA |  |
| **LACM-HC 87092** | Right | M2 | 115 | 82 | Rancho La Brea | Los Angeles | CA |  |
| **UCMP 45265** | Left | M2 | 107.08 | 81.34 | Union Oil Tank Farm | Contra Costa | CA |  |
| **UCMP 114599** | Right | M2 | 102.35 | 73.16 | Homestake Mine Road 1 | Lassen | CA |  |
| **UCMP 41642** | Right | M2 | 109.03 | 80.49 | 5 Oaks Ranch | Sonoma | CA |  |
| **UCMP 1564** | Right | M2 | 103.9 | 77.93 | Kincaide Flat | Tuolumne | CA |  |
| **LACM 149514** | Right | M2 | 120.62 | 81.86 | Simi Valley 7455 | Ventura Co. | CA |  |
| **WSC 11079** | Left | M2 | 109.33 | 80.00 | Diamond Valley Lake, East Dam | Riverside | CA |  |
| **WSC 6523** | Right | M2 | 98.24 | 77.92 | Diamond Valley Lake, West Dam | Riverside | CA |  |
| **SBMNH IP A-115** | Right | M2 | 108.94 | 84.65 |  | Santa Barbara | CA |  |
| **SDSNH 116399** | Right | M2 | 108.35 | 83.18 | Robertson Ranch, East Village (6044) | San Diego | CA |  |
| **SBCM 5.3.298** | Left | M2 | 107.37 | 84.9 | Perris | Riverside | CA |  |
| **LACM-HC 87088** | Left | M2 | 121.66 | 78.52 | Rancho La Brea | Los Angeles | CA |  |
| **WSC 18743** | Right | m2 | 103.6 | 68 | Diamond Valley Lake, West Dam | Riverside | CA |  |
| **WSC 19730** | Right | m2 | 107 | 79.1 | Diamond Valley Lake, West Dam, San Diego Canal | Riverside | CA |  |
| **LC 0260** | Right | m2 | 113.1 | 78.6 | Fullerton | Orange | CA |  |
| **UCMP 198861** | Right | m2 | 110.5 | 79.1 | Rancho La Brea | Los Angeles | CA | Trayler and Dundas 2009 |
| **LACM-HC 87093** | Left | m2 | 125 | 78 | Rancho La Brea | Los Angeles | CA |  |
| **LACM-HC 77** | Right | m2 | 102 | 73 | Rancho La Brea | Los Angeles | CA |  |
| **LACM-HC 87072** | Left | m2 | 116 | 76 | Rancho La Brea | Los Angeles | CA |  |
| **LACM-HC 87073** | Left | m2 | 95 | 65 | Rancho La Brea | Los Angeles | CA |  |
| **LACM-HC 87089** | Right | m2 | 107 | 71 | Rancho La Brea | Los Angeles | CA |  |
| **UCMP 1566** | Left | m2 | 116.5 | 82.69 | Shingle Springs | El Dorado | CA |  |
| **UCMP 35129** | Right | m2 | 115.65 | 75.03 | McKittrick (Sternberg Pit) | Kern | CA |  |
| **LACM 130515** | Left | m2 | 103.75 | 75.59 | Churro Creek Site | San Luis Obispo | CA |  |
| **LACM 5316** | Left | m2 | 102.36 | 70.34 | Irwindale | Los Angeles | CA |  |
| **LACM 149514** | Right | m2 | 129.56 | 83.4 | Simi Valley 7455 | Ventura Co. | CA |  |
| **LACM 128927** | Right | m2 | 114.82 | 79.8 | Carrizo Plain | San Louis Obispo | CA |  |
| **LACM 152669** | Right | m2 | 88.62 | 73.09 | Lakes at Thousand Oaks | Ventura | CA |  |
| **WSC 19721** | Left | m2 | 114.52 | 75.10 | Diamond Valley Lake, West Dam | Riverside | CA |  |
| **SBMNH IP A-115** | Right | m2 | 109.78 | 85.90 |  | Santa Barbara | CA |  |
| **SBMNH Pes-320** | Left | m2 | 112.80 | 84.39 | Corallitos Canyon | Santa Barbara | CA |  |
| **SBMNH-VP 3342** | Left | m2 | 107.42 | 69.73 |  | Santa Barbara | CA |  |
| **SBMNH-VP 3341** | Left | m2 | 101.49 | 78.36 |  | Santa Barbara | CA |  |
| **SDSNH 116399** | Right | m2 | 102.69 | 71.74 | Robertson Ranch, East Village (6044) | San Diego | CA |  |
| **SDSNH 49926** | Left | m2 | 112.09 | 75.47 | Route 54 West | San Diego | CA |  |
| **SNSNH 86541** | Right | m2 | 104.62 | 68.51 | Wanis View Estates #5 (4748B) | San Diego | CA |  |
| **LACM-HC 38** | Left | m2 | 102 | 72 | Rancho La Brea | Los Angeles | CA |  |
| **WSC 9964** | Left | M1 | 80.7 | 66.3 | Diamond Valley Lake, West Dam | Riverside | CA |  |
| **UCMP 198860** | Right | M1 | 97.1 | 69.9 | Rancho La Brea | Los Angeles | CA | Trayler and Dundas 2009 |
| **LACM-HC 87129** | Left | M1 | 83 | 68 | Rancho La Brea | Los Angeles | CA |  |
| **LACM-HC 87083** | Right | M1 | 84 | 66 | Rancho La Brea | Los Angeles | CA |  |
| **LACM-HC 87081** | Left | M1 | 86 | 69 | Rancho La Brea | Los Angeles | CA |  |
| **UCMP 198861** | Right | m1 | 108.5 | 66.5 | Rancho La Brea | Los Angeles | CA | Trayler and Dundas 2009 |
| **LACM-HC 87093** | Left | m1 | 96 | 62 | Rancho La Brea | Los Angeles | CA |  |
| **LACM-HC 38** | Right | m1 | 86 | 59 | Rancho La Brea | Los Angeles | CA |  |
| **UCMP 134904** | Left | m1 | 92.33 | 57.83 | Fossil Cave | Siskiyou | CA |  |
| **LACM 128927** | Right | m1 | 84.2 | 62.54 | Carrizo Plain | San Louis Obispo | CA |  |
| **WSC 817** | Right | m1 | 99.64 | 69.30 | Diamond Valley Lake, West Dam | Riverside | CA |  |
| **WSC 180** | Left | dP4 | 70.9 | 54.7 | Diamond Valley Lake, West Dam | Riverside | CA |  |
| **UCMP 22575** | Left | dP4 | 72.12 | 50.93 | Antioch | Contra Costa | CA |  |
| **WSC 262** | Right | dp4 | 67.9 | 47 | Diamond Valley Lake, West Dam | Riverside | CA |  |
| **LACM 3025** | Left | dp4 | 87.31 | 56.79 | Imperial Highway, La Habra | Los Angeles | CA |  |
| **SBCM A3005-159** | Left | dp4 | 58.1 | 36.3 | Jurupa Valley | Riverside | CA |  |
| **LACM-HC 87093** | Left | dp4 | 75.79 | 48.82 | Rancho La Brea | Los Angeles | CA |  |
| **LACM-HC 475** | Right | dp4 | 71.47 | 50.96 | Rancho La Brea | Los Angeles | CA |  |
| **LACM-HC 1631** | Left | dp4 | 68.02 | 43.59 | Rancho La Brea | Los Angeles | CA |  |
| **RSF 0201** | Right | dP3 | 42.57 | 38.62 | Rancho La Brea | Los Angeles | CA |  |
| **LACM-P23 4766** | Right | dP3 | 42.93 | 38.20 | Rancho La Brea | Los Angeles | CA |  |
| **RSF 0201** | Left | dp3 | 46.39 | 35.45 | Rancho La Brea | Los Angeles | CA |  |
| **SBCM A3005-159** | Left | dp3 | 43.9 | 29.7 | Jurupa Valley | Riverside | CA |  |
| **LACM-P23 26389** | Left | dp3 | 47.29 | 37.33 | Rancho La Brea | Los Angeles | CA |  |
| **LACM-HC 475** | Right | dp3 | 46.49 | 34.57 | Rancho La Brea | Los Angeles | CA |  |
| **LACM-HC 1631** | Left | dp3 | 47.02 | 33.25 | Rancho La Brea | Los Angeles | CA |  |
| **WSC 180** | Left | dP2 | 31.1 | 26.6 | Diamond Valley Lake, West Dam | Riverside | CA |  |
| **RSF 0201** | Right | dP2 | 30.55 | 30.31 | Rancho La Brea | Los Angeles | CA |  |
| **UCMP 5049** | Right | dP2 | 30.84 | 27.62 | Potter Creek Cave | Shasta | CA |  |
| **UCMP 8204** | Left | p2 | 30.32 | 22.89 | Potter Creek Cave | Shasta | CA |  |
| **SBCM A3005-159** | Left | p2 | 26.6 | 19.1 | Jurupa Valley | Riverside | CA |  |
| **LACM-HC 87080?** | Left | p2 | 33.55 | 27.61 | Rancho La Brea | Los Angeles | CA |  |
| **LACM-P23 26389** | Left | p2 | 27.72 | 24.29 | Rancho La Brea | Los Angeles | CA |  |
| **LACM-HC 475** | Right | p2 | 27.99 | 25.38 | Rancho La Brea | Los Angeles | CA |  |
| **Irvingtonian specimens referred to *M. pacificus* included in this study** | | | | | | | | |
| **WSC 10055** | Left | M3 | 183.0 | 91.3 | Copper Canyon | Riverside | CA |  |
| **SBCM A2658-7** | Right | m3 | 180.45 | 79.5 | Murrieta | Riverside | CA |  |
| **WSC 10055** | Left | m3 | 186.0 | 78.0 | Copper Canyon | Riverside | CA |  |
| **IMNH 39139** |  | m3 | 201.2 | 90.1 | Gay Mine | Bingham | ID |  |
| **IMNH 39156** |  | m3 | 192.9 | 81.3 | Gay Mine | Bingham | ID |  |
