# Supplementary material for: Mammut pacificus sp. nov., a newly recognized species of mastodon from the Pleistocene of western North America
Source: PeerJ. 2019 Mar 27;7:e6614. doi: 10.7717/peerj.6614 (PMC6441323; doi:10.7717/peerj.6614)
Supplement: Supplemental Information 2 — Measurements (in mm) of Mammut americanum specimens examined in this study. [file peerj-07-6614-s002.docx]

| **Rancholabrean teeth of *M. americanum* included in this study** | | | | | | | | |
| --- | --- | --- | --- | --- | --- | --- | --- | --- |
| **Specimen** | **Side** | **Element** | **Length** | **Width** | **Locality** | **County** | **State/Prov/Country** | **Reference** |
| **NMC 8060** |  | M3 | 164.3 | 97 | Dawson Loc 20 |  | AK | Harington, 1977 |
| **NMC 333** |  | M3 | 149.7 | 94.15 | McQuesten River |  | AK | Harington, 1977 |
| **M&M mastodon** | Right | M3 | 188.2 | 98.2 | M&M site | Yavapai | AZ | Pasenko 2011 |
| **DMNH 69331** | Left | M3 | 163 | 97.5 | Zeigler Reservoir | Pitkin | CO |  |
| **DMNH 69327** | Left | M3 | 164 | 100.2 | Zeigler Reservoir | Pitkin | CO |  |
| **DMNH 60675** | Right | M3 | 164 | 98.3 | Zeigler Reservoir | Pitkin | CO |  |
| **DMNH 69943** | Left | M3 | 163 | 101.2 | Zeigler Reservoir | Pitkin | CO |  |
| **UF 135709** |  | M3 | 164.8 | 94.8 | Aucilla River | Jefferson/Taylor | FL | Green 2006 |
| **UF 192226** |  | M3 | 175.5 | 103.4 | Aucilla River | Jefferson/Taylor | FL | Green 2006 |
| **UF 135724** |  | M3 | 159.1 | 88.7 | Aucilla River | Jefferson/Taylor | FL | Green 2006 |
| **UF 135722** |  | M3 | 168.3 | 105.7 | Aucilla River | Jefferson/Taylor | FL | Green 2006 |
| **UF 135725** |  | M3 | 171.3 | 105.2 | Aucilla River | Jefferson/Taylor | FL | Green 2006 |
| **UF 135713** |  | M3 | 177.3 | 99.2 | Aucilla River | Jefferson/Taylor | FL | Green 2006 |
| **UF 135726** |  | M3 | 187.7 | 100.6 | Aucilla River | Jefferson/Taylor | FL | Green 2006 |
| **UF 135723** |  | M3 | 197.2 | 110.4 | Aucilla River | Jefferson/Taylor | FL | Green 2006 |
| **CP1-RM 4** |  | M3 | 197.4 | 106 | Aucilla River | Jefferson/Taylor | FL | Green 2006 |
| **CP1-BM 3** |  | M3 | 194.3 | 101.3 | Aucilla River | Jefferson/Taylor | FL | Green 2006 |
| **DMAS 705** |  | M3 | 143.2 | 82.2 | Daytona Beach | Volusia | FL | Green 2006 |
| **UF 40001** |  | M3 | 187 | 104.8 | Ichetucknee River | Columbia | FL | Green 2006 |
| **UF 206884** | Right | M3 | 182.4 | 93.3 | Munroe Sloth Site | Madison | FL | Green 2006 |
| **JS 2** |  | M3 | 163.5 | 105.8 | Santa Fe River | Alachua | FL | Green 2006 |
| **JS 1** |  | M3 | 173.2 | 97.4 | Santa Fe River | Alachua | FL | Green 2006 |
| **UF 210253** |  | M3 | 168.8 | 94.9 | Waccasassa River 3 | Levy | FL | Green 2006 |
| **UF 13505** |  | M3 | 186 | 95.6 | West Palm Beach | Palm Beach | FL | Green 2006 |
| **CCQ-90-X-Xc** | Left | M3 | 184 | 104 | Hannahatchee Creek | Stewart | GA | Schwimmer 1991 |
| **unknown** | Left | M3 | 175 | 102 | East St. Louis | St. Clair | IL | Hay 1923 |
| **LACM 130386** | Left | M3 | 194.5 | 111.37 | Fairfield | Bureau | IL |  |
| **71.3.39.11** | Right | M3 | 194.5 | 103.5 | Dollens | Madison | IN | Richards et al. 1987 |
| **71.967.41** | Right | M3 | 185 | 108 | Haysville | Dubois | IN | Richards 1984 |
| **LM 90** | Right | M3 | 176 | 106.5 | Lewis | Wabash | IN | Hunt and Richards 1992 |
| **71.976.36.1** | Left | M3 | 203 | 106 | Madison Co. | Madison | IN | Richards 1984 |
| **USNM 8204** |  | M3 | 185 | 95 | Pulaski Co. | Pulaski | IN | Gidley 1926 |
| **71.981.91** | Left | M3 | 154 | 96 | Richmond | Wayne | IN | Richards 1984 |
| **CMC VP-1** | Left | M3 | 155 | 96 | Rochester | Fulton | IN | Woodman and Branstrator 2008 |
| **LACM 154685** | Left | M3 | 150.04 | 87.85 | Pine Valley | Allen | IN |  |
| **LSUMG V-5001** | Right | M3 | 163 | 98.4 | Angola | West Feliciana | LA |  |
| **LSUMG V-17071** | Right | M3 | 196.6 | 118 | Tunica Hills | West Feliciana | LA |  |
| **UAHMP 311** |  | M3 | 158 | 83 | Hidalgo |  | Mexico | Bravo-Cuevas et al. 2015 |
| **Unknown** |  | M3 | 161.1 | 95 | Boney Spring | Benton | MO | Saunders 1977 (via GraphClick) |
| **Unknown** |  | M3 | 169.3 | 89.1 | Boney Spring | Benton | MO | Saunders 1977 (via GraphClick) |
| **Unknown** |  | M3 | 166.3 | 91 | Boney Spring | Benton | MO | Saunders 1977 (via GraphClick) |
| **Unknown** |  | M3 | 174.4 | 99 | Boney Spring | Benton | MO | Saunders 1977 (via GraphClick) |
| **Unknown** |  | M3 | 180.7 | 93.8 | Boney Spring | Benton | MO | Saunders 1977 (via GraphClick) |
| **Unknown** |  | M3 | 180.7 | 103.8 | Boney Spring | Benton | MO | Saunders 1977 (via GraphClick) |
| **Unknown** |  | M3 | 181.7 | 100.7 | Boney Spring | Benton | MO | Saunders 1977 (via GraphClick) |
| **Unknown** |  | M3 | 191.8 | 107.7 | Boney Spring | Benton | MO | Saunders 1977 (via GraphClick) |
| **Unknown** |  | M3 | 186.7 | 101.8 | Boney Spring | Benton | MO | Saunders 1977 (via GraphClick) |
| **Unknown** |  | M3 | 190.9 | 102.6 | Boney Spring | Benton | MO | Saunders 1977 (via GraphClick) |
| **Unknown** |  | M3 | 191.8 | 101.7 | Boney Spring | Benton | MO | Saunders 1977 (via GraphClick) |
| **Unknown** |  | M3 | 192.8 | 108.7 | Boney Spring | Benton | MO | Saunders 1977 (via GraphClick) |
| **Unknown** |  | M3 | 196.7 | 108.6 | Boney Spring | Benton | MO | Saunders 1977 (via GraphClick) |
| **Unknown** |  | M3 | 203.6 | 110.7 | Boney Spring | Benton | MO | Saunders 1977 (via GraphClick) |
| **Unknown** |  | M3 | 204.5 | 106.4 | Boney Spring | Benton | MO | Saunders 1977 (via GraphClick) |
| **Unknown** |  | M3 | 205.8 | 114.5 | Boney Spring | Benton | MO | Saunders 1977 (via GraphClick) |
| **Unknown** |  | M3 | 214.5 | 110.3 | Boney Spring | Benton | MO | Saunders 1977 (via GraphClick) |
| **Unknown** |  | M3 | 144.3 | 86.6 | Trolinger Spring | Hickory | MO | Saunders 1977 (via GraphClick) |
| **Unknown** |  | M3 | 151.4 | 92.7 | Trolinger Spring | Hickory | MO | Saunders 1977 (via GraphClick) |
| **Unknown** |  | M3 | 154.7 | 88.7 | Trolinger Spring | Hickory | MO | Saunders 1977 (via GraphClick) |
| **Unknown** |  | M3 | 179.3 | 101.1 | Trolinger Spring | Hickory | MO | Saunders 1977 (via GraphClick) |
| **Unknown** |  | M3 | 179.3 | 101.1 | Trolinger Spring | Hickory | MO | Saunders 1977 (via GraphClick) |
| **CMC VP 145** | Right | M3 | 151.2 | 89.5 | Clermont County | Clermont | OH |  |
| **CMC VP 7468** | Left | M3 | 197.5 | 110 | Hamilton County | Hamilton | OH |  |
| **CMC VP 47** | Right | M3 | 166.0 | 103.2 | Shaw Ave, Hyde Park, Cincinnati | Hamilton | OH |  |
| **G27831** | Right | M3 | 205.4 | 106.5 | Springfield | Clark | OH |  |
| **UCMP 49758** | Left | M3 | 185.04 | 109.9 | Connor Creek | Young | TX |  |
| **TMM 30967-773** | Right | M3 | 163 | 91 | Ingleside | San Patricio | TX | Lundelius 1972 |
| **TMM 30967-985** | Left | M3 | 167 | 97 | Ingleside | San Patricio | TX | Lundelius 1972 |
| **BYUVP 4378** | Right | M3 | 151 | 87 | Sanpete Co. | Sanpete | UT | Miller 1987 |
| **BYUVP 4379** | Right | M3 | 152 | 84 | Sanpete Co. | Sanpete | UT | Miller 1987 |
| **UWBM 83312** | Right | M3 | 161.63 | 88.82 | Shine C0700 | Jefferson | WA |  |
| **YG 26.1** |  | M3 | 148.93 | 88.56 |  |  | Yukon |  |
| **YG 43.2** |  | M3 | 159.56 | 88.23 |  |  | Yukon |  |
| **YG 139.5** |  | M3 | 154.98 | 88.6 |  |  | Yukon |  |
| **YG 361.32** |  | M3 | 155.25 | 89.52 |  |  | Yukon |  |
| **NMC 8707** | Left | M3 | 146.48 | 87.29 | "Junction, Independence + Hunker Creeks, Dawson area, Y.T." - "under 60' frozen tundra, above gravels" |  | Yukon |  |
| **NCSM 087** | Right | M3 | 155 | 90.8 |  | Pender | NC |  |
| **NCSM 086** | Right | M3 | 174.7 | 100.5 |  | Pender | NC |  |
| **NCSM 079** | Right | M3 | 177.8 | 102.6 | T.E. Cooper Farm | Brunswick | NC |  |
| **NCSM-013** | Right | M3 | 152.8 | 86 | Walton Farm |  | NC |  |
| **USNM 437571** | Right | M3 | 185 | 96 | Salvo, Hatteras Island | Dare | NC |  |
| **NYSM-VP 103** | Right | M3 | 172.2 | 100.2 | Arborio | Orange | NY |  |
| **F:AM 27009** |  | m3 | 187 | 101 | Fairbanks |  | AK | Osborn, 1936 |
| **NMC 14252** |  | m3 | 145.9 | 79.22 | Old Crow Loc 13 |  | AK |  |
| **No Number (Bowie)** | Right | m3 | 171.1 | 82.8 | San Simon Valley | Cochise | AZ | Pasenko, 2012 |
| **DMNH 58820** | Left | m3 | 174.8 | 93 | Jefferson Co. | Jefferson | CO |  |
| **DMNH 69928** | Right | m3 | 185.8 | 96.1 | Zeigler Reservoir | Pitkin | CO |  |
| **DMNH 69927** | Right | m3 | 196 | 100.9 | Zeigler Reservoir | Pitkin | CO |  |
| **DMNH 69922** | Left | m3 | 174.8 | 87.8 | Zeigler Reservoir | Pitkin | CO |  |
| **DMNH 69923** | Right | m3 | 171 | 101.4 | Zeigler Reservoir | Pitkin | CO |  |
| **DMNH 70763** | Left | m3 | 174.8 | 106.8 | Zeigler Reservoir | Pitkin | CO |  |
| **DMNH 69926** | Left | m3 | 202.4 | 90.6 | Zeigler Reservoir | Pitkin | CO |  |
| **DMNH 69929** | Right | m3 | 173 | 91.4 | Zeigler Reservoir | Pitkin | CO |  |
| **DMNH 69925** | Right | m3 | 189.4 | 95.7 | Zeigler Reservoir | Pitkin | CO |  |
| **UF 135705** |  | m3 | 183 | 89.5 | Aucilla River | Jefferson/Taylor | FL | Green 2006 |
| **UF 135712** |  | m3 | 158.3 | 92.9 | Aucilla River | Jefferson/Taylor | FL | Green 2006 |
| **UF 135721** |  | m3 | 164 | 100.6 | Aucilla River | Jefferson/Taylor | FL | Green 2006 |
| **UF 211300** |  | m3 | 167 | 101.2 | Aucilla River | Jefferson/Taylor | FL | Green 2006 |
| **UF 135720** |  | m3 | 173 | 89.9 | Aucilla River | Jefferson/Taylor | FL | Green 2006 |
| **UF 135714** |  | m3 | 177.2 | 89.1 | Aucilla River | Jefferson/Taylor | FL | Green 2006 |
| **UF 135715** |  | m3 | 186.1 | 96.5 | Aucilla River | Jefferson/Taylor | FL | Green 2006 |
| **UF 135703** |  | m3 | 187 | 94.9 | Aucilla River | Jefferson/Taylor | FL | Green 2006 |
| **UF 135704** |  | m3 | 187.7 | 96.6 | Aucilla River | Jefferson/Taylor | FL | Green 2006 |
| **UF 135717** |  | m3 | 187.9 | 95.5 | Aucilla River | Jefferson/Taylor | FL | Green 2006 |
| **UF 135701** |  | m3 | 199.5 | 99.4 | Aucilla River | Jefferson/Taylor | FL | Green 2006 |
| **UF 135718** |  | m3 | 216.5 | 106.2 | Aucilla River | Jefferson/Taylor | FL | Green 2006 |
| **CP1-BM 1** |  | m3 | 155 | 94.5 | Aucilla River | Jefferson/Taylor | FL | Green 2006 |
| **CP1-BM 8** |  | m3 | 201.3 | 111.6 | Aucilla River | Jefferson/Taylor | FL | Green 2006 |
| **UF 200661** |  | m3 | 174.8 | 91.5 | Aucilla River Sloth Hole | Jefferson/Taylor | FL | Green 2006 |
| **UF 200681** |  | m3 | 182.3 | 96 | Aucilla River Sloth Hole | Jefferson/Taylor | FL | Green 2006 |
| **DMAS 578** |  | m3 | 191.4 | 98.1 | Daytona Beach | Volusia | FL | Green 2006 |
| **CP1-HM 9** |  | m3 | 185 | 98.4 | Dickerson Pit | St. Lucie | FL | Green 2006 |
| **UF 51192** |  | m3 | 178.4 | 89.7 | Palm Beach Farm | Palm Beach | FL | Green 2006 |
| **G47** | Right | m3 | 163 | 90.2 | St. Petersburg | Pinellas | FL |  |
| **UF 210251** |  | m3 | 181 | 95.7 | Waccasassa River 3 | Levy | FL | Green 2006 |
| **UF 210252** |  | m3 | 189.6 | 96.9 | Waccasassa River 3 | Levy | FL | Green 2006 |
| **UF 18504** |  | m3 | 187.6 | 95.9 | West Palm Beach | Palm Beach | FL | Green 2006 |
| **FMP 39395** |  | m3 | 167 | 85 | Croven Cave | Monroe | IL |  |
| **No number** |  | m3 | 191 | 104 | Hawthorne Farm | Ford | IL |  |
| **FM P UC 1509** |  | m3 | 183.2 | 96.8 | Kittleson Farm | Kendall | IL |  |
| **FM P 14057** |  | m3 | 197.7 | 108.5 | Minooka | Grundy | IL |  |
| **FMP 14591** |  | m3 | 184.3 | 100.6 | Minooka | Grundy | IL |  |
| **FMP 61120** |  | m3 | 182.4 | 99.1 | Whitewillow | Kendall | IL |  |
| **FM P 12780** |  | m3 | 198.7 | 102.4 | Whitewillow | Kendall | IL |  |
| **FMP 12777** |  | m3 | 199.9 | 108.1 | Whitewillow | Kendall | IL |  |
| **IUPUI-2** |  | m3 | 170.2 | 91.7 | Christensen | Hancock | IN |  |
| **DMNH 1496** | Right | m3 | 195.9 | 97.7 | De Kalb Co. | De Kalb | IN |  |
| **FMP 1173** |  | m3 | 202.3 | 99 | Delco Farm | Lake | IN |  |
| **71.3.39.1** | Left | m3 | 196 | 108 | Dollens | Madison | IN | Richards et al. 1987 |
| **LM 90** | Left | m3 | 188.5 | 106 | Lewis | Wabash | IN | Hunt and Richards 1992 |
| **71.981.70** | Left | m3 | 197 | 102 | Orange Co. | Orange | IN | Richards 1984 |
| **USNM 8204** |  | m3 | 180 | 102 | Pulaski Co. | Pulaski | IN | Gidley 1926 |
| **CMC VP-1** | Right | m3 | 164 | 99 | Rochester (Overmeyer) | Fulton | IN | Woodman and Branstrator 2008 |
| **JMM VP-20** | Right | m3 | 171.3 | 95.8 | Wayne Co. | Wayne | IN |  |
| **KU 4929** |  | m3 | 187 | 96.2 | McPherson | McPherson | KS | Hibbard 1952 |
| **LACM 154598** | Right | m3 | 203 | 105.27 | Arkansas River 50mi S of Wichita | Cowley | KS |  |
| **UNSM 516-62** |  | m3 | 199.1 | 101.1 | Big Bone Lick | Boone | KY |  |
| **MNHN 1643 Lectotype** |  | m3 | 202.7 | 101.1 | Big Bone Lick | Boone | KY |  |
| **MNHN 1644** |  | m3 | 165 | 90.9 | Big Bone Lick | Boone | KY |  |
| **MNHN 1657** |  | m3 | 182.5 | 98.8 | Big Bone Lick | Boone | KY |  |
| **MNHN 1640** |  | m3 | 201.3 | 116.5 | Big Bone Lick | Boone | KY |  |
| **MNHN 315** |  | m3 | 181 | 94.3 | Big Bone Lick | Boone | KY |  |
| **LSUMG V-14527** | Left | m3 | 183.3 | 98 | Tunica Hills | West Feliciana | LA |  |
| **LSUMG V-14529** | Right | m3 | 183.9 | 94.8 | Tunica Hills | West Feliciana | LA |  |
| **LSUMG V-1604** | Left | m3 | 213.8 | 105.6 | Ward's Creek | East Baton Rouge | LA |  |
| **LSUMG V-8864** | Right | m3 | 226.5 | 110 | Ward's Creek | East Baton Rouge | LA |  |
| **LSUMG V-19517** | Left | m3 | 187.2 | 105.8 | unknown | Adams? | LA/MS |  |
| **LSUMG V-19521** | Right | m3 | 185.7 | 104.3 | unknown | Adams? | LA/MS |  |
| **GML2145/WCMFL289** | Left | m3 | 191.6 | 101.2 | unknown | Adams? | LA/MS |  |
| **WCMFL13** | Right | m3 | 188.6 | 99.9 | unknown | Adams? | LA/MS |  |
| **Yale unknown** |  | m3 | 188 | 93 | Natchez | Adams | MS | Hay 1923 |
| **Unknown** |  | m3 | 172.2 | 92.2 | Boney Spring | Benton | MO | Saunders 1977 (via GraphClick) |
| **Unknown** |  | m3 | 175.2 | 93.4 | Boney Spring | Benton | MO | Saunders 1977 (via GraphClick) |
| **Unknown** |  | m3 | 175.3 | 92.2 | Boney Spring | Benton | MO | Saunders 1977 (via GraphClick) |
| **Unknown** |  | m3 | 181.5 | 96.2 | Boney Spring | Benton | MO | Saunders 1977 (via GraphClick) |
| **Unknown** |  | m3 | 182.4 | 94.0 | Boney Spring | Benton | MO | Saunders 1977 (via GraphClick) |
| **Unknown** |  | m3 | 183.2 | 98.2 | Boney Spring | Benton | MO | Saunders 1977 (via GraphClick) |
| **Unknown** |  | m3 | 188.1 | 98.2 | Boney Spring | Benton | MO | Saunders 1977 (via GraphClick) |
| **Unknown** |  | m3 | 189.2 | 95.2 | Boney Spring | Benton | MO | Saunders 1977 (via GraphClick) |
| **Unknown** |  | m3 | 193.0 | 101.1 | Boney Spring | Benton | MO | Saunders 1977 (via GraphClick) |
| **Unknown** |  | m3 | 194.7 | 103.1 | Boney Spring | Benton | MO | Saunders 1977 (via GraphClick) |
| **Unknown** |  | m3 | 197.9 | 100.1 | Boney Spring | Benton | MO | Saunders 1977 (via GraphClick) |
| **Unknown** |  | m3 | 201.5 | 104.0 | Boney Spring | Benton | MO | Saunders 1977 (via GraphClick) |
| **Unknown** |  | m3 | 207.4 | 103.2 | Boney Spring | Benton | MO | Saunders 1977 (via GraphClick) |
| **Unknown** |  | m3 | 213.0 | 103.0 | Boney Spring | Benton | MO | Saunders 1977 (via GraphClick) |
| **Unknown** |  | m3 | 209.2 | 109.8 | Boney Spring | Benton | MO | Saunders 1977 (via GraphClick) |
| **Unknown** |  | m3 | 212.1 | 110.6 | Boney Spring | Benton | MO | Saunders 1977 (via GraphClick) |
| **Unknown** |  | m3 | 162.0 | 86.0 | Trolinger Spring | Hickory | MO | Saunders 1977 (via GraphClick) |
| **Unknown** |  | m3 | 173.2 | 89.8 | Trolinger Spring | Hickory | MO | Saunders 1977 (via GraphClick) |
| **Unknown** |  | m3 | 186.8 | 99.5 | Trolinger Spring | Hickory | MO | Saunders 1977 (via GraphClick) |
| **Unknown** |  | m3 | 184.0 | 96.5 | Trolinger Spring | Hickory | MO | Saunders 1977 (via GraphClick) |
| **UNSM 1465** |  | m3 | 183.9 | 98.5 | Beatrice, Gage Co. | Gage | NE |  |
| **UNSM 1685** |  | m3 | 177.9 | 101.5 | Hebron | Thayer | NE |  |
| **UNSM 1657** |  | m3 | 178.2 | 99.8 | Nemaha River | Johnson? | NE |  |
| **UNSM 88534** |  | m3 | 184.7 | 100.6 | Rh-101 |  | NE |  |
| **NMMNH P-25546** | Right | m3 | 153 | 79.5 | Las Lunas | Valencia | NM | Lucas and Morgan 1997 |
| **NMMNH P-25098** | Left | m3 | 168 | 94.5 | Lemitar | Socorro | NM | Lucas and Morgan 1997 |
| **No number** |  | m3 | 178 | 93 | Trapped Rock | McKinley | NM |  |
| **Boonshoft AccN 225-46** | Left | m3 | 188.4 | 103.6 | Darke Co. | Darke | OH |  |
| **JMM VP-2992** | Right | m3 | 222.2 | 106.9 | Harper | Logan | OH |  |
| **CMC VP 1120** | Left | m3 | 194 | 100.1 | Mill Creek, Evendale | Hamilton | OH |  |
| **CMNH 10334** |  | m3 | 160.6 | 87 | Portage Co. | Portage | OH |  |
| **No number** |  | m3 | 136 | 79 | Lac Saint-Jean |  | Quebec |  |
| **TMM 30967-1650** |  | m3 | 195 | 100 | Ingleside | San Patricio | TX | Lundelius 1972 |
| **TMM 30967-339** |  | m3 | 168 | 93 | Ingleside | San Patricio | TX | Lundelius 1972 |
| **TMM 30967-352** |  | m3 | 200 | 106 | Ingleside | San Patricio | TX | Lundelius 1972 |
| **TMM 30967-414** |  | m3 | 198 | 102 | Ingleside | San Patricio | TX | Lundelius 1972 |
| **TMM 30967-904** |  | m3 | 183 | 96 | Ingleside | San Patricio | TX | Lundelius 1972 |
| **BYUVP 4378** | Right | m3 | 170 | 84 | Sanpete Co. | Sanpete | UT | Miller 1987 |
| **BYUVP 4379** | Right | m3 | 169 | 81 | Sanpete Co. | Sanpete | UT | Miller 1987 |
| **No number** |  | m3 | 165.6 | 89.5 | Saltville | Smyth | VA |  |
| **UWBM 88099** | Left | m3 | 165.68 | 85.58 | Winlock area C0856 | Lewis | WA |  |
| **UWBM 14491** | Right | m3 | 164.6 | 78.01 | Port Angeles | Clallam | WA |  |
| **No number** |  | m3 | 177 | 97 | Cottonwood Creek | Roane | WV |  |
| **YG 361.31** |  | m3 | 163.05 | 82.34 |  |  | Yukon |  |
| **NMC 42552** | Left | m3 | 157.75 | 81.41 | "Sixtymile Loc. 3, Y.T." |  | Yukon |  |
| **LACM 117689** | Left | m3 | 175.13 | 80.16 | Minaca Mesa | Chihuahua | Mexico |  |
| **NCSM 083** | Left | m3 | 190.5 | 90.7 | Goldsboro | Wayne | NC |  |
| **NCSM 041** | Right | m3 | 187.3 | 93.6 | Maysville | Jones | NC |  |
| **NCSM 047** | Left | m3 | 190.6 | 94.4 | Maysville | Jones | NC |  |
| **NCSM-013** | Left | m3 | 153.4 | 87.8 | Walton Farm |  | NC |  |
| **LACM 1864** | Left | m3 | 168.97 | 81.6 | Lago de Chapala, Loc.1135 Chapala Fm | Jalisco | Mexico |  |
| **NYSM-VP 54** | Right | m3 | 196.7 | 97.6 | Perkinsville | Steuben | NY |  |
| **M&M** | Right | M2 | 123 | 88 | M&M site | Yavapai | AZ | Pasenko, 2011 |
| **No number (Bowie)** | Right | M2 | 113.7 | 85.6 | San Simon Valley | Cochise | AZ | Pasenko, 2012 |
| **DMNH 69333** | Right | M2 | 135.9 | 97.8 | Zeigler Reservoir | Pitkin | CO |  |
| **DMNH 69331** | Left | M2 | 117.2 | 92.8 | Zeigler Reservoir | Pitkin | CO |  |
| **DMNH 60700** | Right | M2 | 125.2 | 98.4 | Zeigler Reservoir | Pitkin | CO |  |
| **DMNH 70760** | Left | M2 | 126.3 | 92.4 | Zeigler Reservoir | Pitkin | CO |  |
| **DMNH 70759** | Left | M2 | 128.1 | 97.3 | Zeigler Reservoir | Pitkin | CO |  |
| **DMNH 70773** | Right | M2 | 125.6 | 96.3 | Zeigler Reservoir | Pitkin | CO |  |
| **DMNH 70782** | Right | M2 | 123.5 | 94.1 | Zeigler Reservoir | Pitkin | CO |  |
| **DMNH 69327** | Left | M2 | 122.7 | 95.9 | Zeigler Reservoir | Pitkin | CO |  |
| **DMNH 60675** | Right | M2 | 113 | 93.3 | Zeigler Reservoir | Pitkin | CO |  |
| **DMNH 69335** | Left | M2 | 120 | 89.3 | Zeigler Reservoir | Pitkin | CO |  |
| **DMNH 70758** | Right | M2 | 119.4 | 91 | Zeigler Reservoir | Pitkin | CO |  |
| **UF 40001** | Right | M2 | 129.8 | 89.9 | Ichetucknee River | Columbia | FL | Green and Hulbert 2005 |
| **UF 135709** | Right | M2 | 107.1 | 83.9 | Aucilla River | Jefferson/Taylor | FL | Green and Hulbert 2005 |
| **UF 13505** | Right | M2 | 118.2 | 87.5 | West Palm Beach | Palm Beach | FL | Green and Hulbert 2005 |
| **UF 135564** | Right | M2 | 108.9 | 89.8 | Wekiwa River 1 | Seminole | FL | Green and Hulbert 2005 |
| **UF 192226** | Left | M2 | 123.5 | 93 | Aucilla 3J | Taylor | FL | Green and Hulbert 2005 |
| **UF 124351** | Right | M2 | 119.1 | 90.7 | Wakulla Springs | Wakulla | FL | Green and Hulbert 2005 |
| **TA100 - 532** | Right | M2 | 120.5 | 95.1 | Aucilla River | Taylor | FL | Green and Hulbert 2005 |
| **UF 53138** | Right | M2 | 112.2 | 90.6 | Santa Fe River 17A | Gilchrist | FL | Green and Hulbert 2005 |
| **CPI BM 2** | Right | M2 | 113.3 | 90.2 | Aucilla River (Half Mile Rise) | Taylor | FL | Green and Hulbert 2005 |
| **CPI BM 4** | Left | M2 | 112.8 | 97.7 | Aucilla River (Half Mile Rise) | Taylor | FL | Green and Hulbert 2005 |
| **CPI BM 5** | Right | M2 | 111.7 | 97.6 | Aucilla River (Half Mile Rise) | Taylor | FL | Green and Hulbert 2005 |
| **CPI BM 7** | Right | M2 | 107.1 | 81.5 | Aucilla River (Half Mile Rise) | Taylor | FL | Green and Hulbert 2005 |
| **71.3.39.10** | Left | M2 | 119.5 | 95.5 | Dollens | Madison | IN | Richards et al. 1987 |
| **71.3.39.9** | Right | M2 | 121 | 95 | Dollens | Madison | IN | Richards et al. 1987 |
| **LM 90** | Right | M2 | 121 | 89.5 | Lewis | Wabash | IN | Hunt and Richards 1992 |
| **USNM 8204** |  | M2 | 119 | 90 | Pulaski Co. | Pulaski | IN | Gidley 1926 |
| **CMC VP-1** | Left | M2 | 100 | 84 | Rochester | Fulton | IN | Woodman and Branstrator 2008 |
| **CMC VP 7451** | Right | M2 | 125.6 | 92.1 | Big Bone Lick? | Boone | KY |  |
| **CMC VP 885** | Left | M2 | 123.8 | 92.3 |  |  | KY |  |
| **LSUMG V-634** | Right | M2 | 121 | 99.8 | Jones Creek | East Baton Rouge | LA |  |
| **LSUMG V-14509** | Right | M2 | 122.6 | 91.9 | Tunica Hills | West Feliciana | LA |  |
| **LSUMG V-8854** | Right | M2 | 124.4 | 117.7 | Ward's Creek | East Baton Rouge | LA |  |
| **Unknown** |  | M2 | 110.1 | 88.8 | Boney Spring | Benton | MO | Saunders 1977 (via GraphClick) |
| **Unknown** |  | M2 | 111.2 | 85.8 | Boney Spring | Benton | MO | Saunders 1977 (via GraphClick) |
| **Unknown** |  | M2 | 113.0 | 83.0 | Boney Spring | Benton | MO | Saunders 1977 (via GraphClick) |
| **Unknown** |  | M2 | 118.1 | 85.7 | Boney Spring | Benton | MO | Saunders 1977 (via GraphClick) |
| **Unknown** |  | M2 | 118.1 | 93.8 | Boney Spring | Benton | MO | Saunders 1977 (via GraphClick) |
| **Unknown** |  | M2 | 118.1 | 92.7 | Boney Spring | Benton | MO | Saunders 1977 (via GraphClick) |
| **Unknown** |  | M2 | 118.9 | 90.6 | Boney Spring | Benton | MO | Saunders 1977 (via GraphClick) |
| **Unknown** |  | M2 | 121.9 | 87.7 | Boney Spring | Benton | MO | Saunders 1977 (via GraphClick) |
| **Unknown** |  | M2 | 121.0 | 90.7 | Boney Spring | Benton | MO | Saunders 1977 (via GraphClick) |
| **Unknown** |  | M2 | 123.1 | 86.7 | Boney Spring | Benton | MO | Saunders 1977 (via GraphClick) |
| **Unknown** |  | M2 | 123.0 | 91.7 | Boney Spring | Benton | MO | Saunders 1977 (via GraphClick) |
| **Unknown** |  | M2 | 124.9 | 97.8 | Boney Spring | Benton | MO | Saunders 1977 (via GraphClick) |
| **Unknown** |  | M2 | 126.9 | 98.8 | Boney Spring | Benton | MO | Saunders 1977 (via GraphClick) |
| **Unknown** |  | M2 | 128.8 | 91.8 | Boney Spring | Benton | MO | Saunders 1977 (via GraphClick) |
| **Unknown** |  | M2 | 128.7 | 97.8 | Boney Spring | Benton | MO | Saunders 1977 (via GraphClick) |
| **Unknown** |  | M2 | 128.8 | 96.7 | Boney Spring | Benton | MO | Saunders 1977 (via GraphClick) |
| **Unknown** |  | M2 | 129.7 | 103.7 | Boney Spring | Benton | MO | Saunders 1977 (via GraphClick) |
| **Unknown** |  | M2 | 129.9 | 102.6 | Boney Spring | Benton | MO | Saunders 1977 (via GraphClick) |
| **Unknown** |  | M2 | 130.9 | 100.8 | Boney Spring | Benton | MO | Saunders 1977 (via GraphClick) |
| **Unknown** |  | M2 | 136.5 | 102.6 | Boney Spring | Benton | MO | Saunders 1977 (via GraphClick) |
| **Unknown** |  | M2 | 112.1 | 79.7 | Trolinger Spring | Hickory | MO | Saunders 1977 (via GraphClick) |
| **Unknown** |  | M2 | 113.0 | 88.8 | Trolinger Spring | Hickory | MO | Saunders 1977 (via GraphClick) |
| **Unknown** |  | M2 | 115.1 | 87.8 | Trolinger Spring | Hickory | MO | Saunders 1977 (via GraphClick) |
| **Unknown** |  | M2 | 116.0 | 93.6 | Trolinger Spring | Hickory | MO | Saunders 1977 (via GraphClick) |
| **Unknown** |  | M2 | 123.7 | 95.6 | Trolinger Spring | Hickory | MO | Saunders 1977 (via GraphClick) |
| **G26524** | Right | M2 | 124.6 | 89.5 | 8 mi S of Dayton | Montgomery | OH |  |
| **G25650** | Left | M2 | 105.8 | 92.4 | Carter's Bog | Darke | OH |  |
| **BYUVP 4379** | Right | M2 | 100 | 80 | Sanpete Co. | Sanpete | UT | Miller 1987 |
| **NYSM-VP 102** | Right | M2 | 111.5 | 88.4 | Arborio | Orange | NY |  |
| **USNM 4911** | Left | M2 | 111.6 | 79.7 | Dixie Creek | Baker | OR |  |
| **NCSM 048** | Left | M2 | 105.6 | 90.1 | Maysville | Jones | NC |  |
| **NCSM 045** | Left | M2 | 105.3 | 92.4 | Maysville | Jones | NC |  |
| **No number (Bowie)** | Left | m2 | 105.7 | 74.9 | San Simon Valley | Cochise | AZ | Pasenko, 2012 |
| **DMNH 69928** | Right | m2 | 117.8 | 86.6 | Zeigler Reservoir | Pitkin | CO |  |
| **DMNH 69921** | Left | m2 | 134.9 | 87.8 | Zeigler Reservoir | Pitkin | CO |  |
| **DMNH 69927** | Right | m2 | 127.8 | 90 | Zeigler Reservoir | Pitkin | CO |  |
| **DMNH 69922** | Left | m2 | 109.9 | 81.6 | Zeigler Reservoir | Pitkin | CO |  |
| **DMNH 69923** | Right | m2 | 108.6 | 88.5 | Zeigler Reservoir | Pitkin | CO |  |
| **DMNH 60701** | Left | m2 | 124.6 | 95.3 | Zeigler Reservoir | Pitkin | CO |  |
| **DMNH 69926** | Left | m2 | 113.8 | 84.4 | Zeigler Reservoir | Pitkin | CO |  |
| **DMNH 69929** | Right | m2 | 116.8 | 81 | Zeigler Reservoir | Pitkin | CO |  |
| **DMNH 69925** | Right | m2 | 111.4 | 79.9 | Zeigler Reservoir | Pitkin | CO |  |
| **DMNH 69924** | Right | m2 | 115.1 | 78.9 | Zeigler Reservoir | Pitkin | CO |  |
| **G47** | Right | m2 | 106.2 | 79.8 | St. Petersburg | Pinellas | FL |  |
| **UF 135702** | Right | m2 | 112.5 | 84.1 | Aucilla River | Jefferson/Taylor | FL | Green and Hulbert 2005 |
| **UF 135701** | Right | m2 | 112.5 | 88.5 | Aucilla River | Jefferson/Taylor | FL | Green and Hulbert 2005 |
| **UF 200660** | Left | m2 | 115 | 87.1 | Aucilla River, Sloth Hole | Taylor | FL | Green and Hulbert 2005 |
| **UF 135703** | Left | m2 | 100.5 | 78.6 | Aucilla River | Taylor | FL | Green and Hulbert 2005 |
| **UF 200656** | Right | m2 | 124.4 | 100.6 | Aucilla River, Sloth Hole | Taylor | FL | Green and Hulbert 2005 |
| **UF 211300** | Left | m2 | 108 | 82 | Aucilla River | Taylor | FL | Green and Hulbert 2005 |
| **UF 135711** | Right | m2 | 120.7 | 98.3 | Aucilla River | Taylor | FL | Green and Hulbert 2005 |
| **DMAS 578** | Left | m2 | 110 | 86.1 | Daytona Beach Bone Bed | Volusia | FL | Green and Hulbert 2005 |
| **UF 51194** | Left | m2 | 108.5 | 84.8 | West Palm Beach | Palm Beach | FL | Green and Hulbert 2005 |
| **CPI HM 8** | Left | m2 | 99.6 | 80.1 | Dickerson Pit | St. Lucie | FL | Green and Hulbert 2005 |
| **DMNH 1496** | Right | m2 | 103.3 | 78.9 | DeKalb Co. | DeKalb | IN |  |
| **71.3.39.1** | Left | m2 | 114.5 | 98 | Dollens | Madison | IN | Richards et al. 1987 |
| **LM 90** | Left | m2 | 122 | 98 | Lewis | Wabash | IN | Hunt and Richards 1992 |
| **USNM 8204** |  | m2 | 120 | 90 | Pulaski Co. | Pulaski | IN | Gidley 1926 |
| **CMC VP-1** | Right | m2 | 102 | 82 | Rochester | Fulton | IN | Woodman and Branstrator 2008 |
| **CMC VP 7515** | Right | m2 | 129.2 | 94.5 | Blue Licks, Nichols County | Nicholas | KY |  |
| **Yale unknown** |  | m2 | 115 | 87 | Natchez | Adams | MS | Hay 1923 |
| **Unknown** |  | m2 | 101.3 | 80.9 | Boney Spring | Benton | MO | Saunders 1977 (via GraphClick) |
| **Unknown** |  | m2 | 104.3 | 81.9 | Boney Spring | Benton | MO | Saunders 1977 (via GraphClick) |
| **Unknown** |  | m2 | 111.3 | 84.0 | Boney Spring | Benton | MO | Saunders 1977 (via GraphClick) |
| **Unknown** |  | m2 | 113.4 | 85.1 | Boney Spring | Benton | MO | Saunders 1977 (via GraphClick) |
| **Unknown** |  | m2 | 119.3 | 83.9 | Boney Spring | Benton | MO | Saunders 1977 (via GraphClick) |
| **Unknown** |  | m2 | 116.2 | 89.0 | Boney Spring | Benton | MO | Saunders 1977 (via GraphClick) |
| **Unknown** |  | m2 | 116.2 | 89.0 | Boney Spring | Benton | MO | Saunders 1977 (via GraphClick) |
| **Unknown** |  | m2 | 116.4 | 82.2 | Boney Spring | Benton | MO | Saunders 1977 (via GraphClick) |
| **Unknown** |  | m2 | 117.3 | 86.7 | Boney Spring | Benton | MO | Saunders 1977 (via GraphClick) |
| **Unknown** |  | m2 | 117.5 | 89.1 | Boney Spring | Benton | MO | Saunders 1977 (via GraphClick) |
| **Unknown** |  | m2 | 118.1 | 91.9 | Boney Spring | Benton | MO | Saunders 1977 (via GraphClick) |
| **Unknown** |  | m2 | 118.2 | 93.3 | Boney Spring | Benton | MO | Saunders 1977 (via GraphClick) |
| **Unknown** |  | m2 | 119.2 | 91.0 | Boney Spring | Benton | MO | Saunders 1977 (via GraphClick) |
| **Unknown** |  | m2 | 119.2 | 91.8 | Boney Spring | Benton | MO | Saunders 1977 (via GraphClick) |
| **Unknown** |  | m2 | 121.5 | 90.0 | Boney Spring | Benton | MO | Saunders 1977 (via GraphClick) |
| **Unknown** |  | m2 | 122.2 | 92.0 | Boney Spring | Benton | MO | Saunders 1977 (via GraphClick) |
| **Unknown** |  | m2 | 123.3 | 88.9 | Boney Spring | Benton | MO | Saunders 1977 (via GraphClick) |
| **Unknown** |  | m2 | 125.2 | 90.9 | Boney Spring | Benton | MO | Saunders 1977 (via GraphClick) |
| **Unknown** |  | m2 | 125.1 | 94.0 | Boney Spring | Benton | MO | Saunders 1977 (via GraphClick) |
| **Unknown** |  | m2 | 127.4 | 91.1 | Boney Spring | Benton | MO | Saunders 1977 (via GraphClick) |
| **Unknown** |  | m2 | 131.4 | 92.0 | Boney Spring | Benton | MO | Saunders 1977 (via GraphClick) |
| **Unknown** |  | m2 | 132.2 | 95.2 | Boney Spring | Benton | MO | Saunders 1977 (via GraphClick) |
| **Unknown** |  | m2 | 133.2 | 88.0 | Boney Spring | Benton | MO | Saunders 1977 (via GraphClick) |
| **Unknown** |  | m2 | 108.3 | 80.8 | Trolinger Spring | Hickory | MO | Saunders 1977 (via GraphClick) |
| **Unknown** |  | m2 | 108.0 | 83.6 | Trolinger Spring | Hickory | MO | Saunders 1977 (via GraphClick) |
| **Unknown** |  | m2 | 109.1 | 84.5 | Trolinger Spring | Hickory | MO | Saunders 1977 (via GraphClick) |
| **Unknown** |  | m2 | 115.1 | 84.6 | Trolinger Spring | Hickory | MO | Saunders 1977 (via GraphClick) |
| **Unknown** |  | m2 | 114.1 | 89.6 | Trolinger Spring | Hickory | MO | Saunders 1977 (via GraphClick) |
| **Unknown** |  | m2 | 119.0 | 81.7 | Trolinger Spring | Hickory | MO | Saunders 1977 (via GraphClick) |
| **Unknown** |  | m2 | 122.0 | 91.4 | Trolinger Spring | Hickory | MO | Saunders 1977 (via GraphClick) |
| **NCSM unknown** | Left | m2 | 122 | 88 | Goldsboro | Wayne | NC | Hay 1923 |
| **NCSM 084** | Left | m2 | 117 | 86.3 |  | Pender | NC |  |
| **NCSM 046** | Left | m2 | 113.3 | 89.1 | Maysville | Jones | NC |  |
| **NMMNH P-25098** | Left | m2 | 98 | 68 | Lemitar | Socorro | NM | Lucas and Morgan 1997 |
| **G25650** | Right | m2 | 112.6 | 87.8 | Carter's Bog | Darke | OH |  |
| **CMC VP 1120** | Left | m2 | 115 | 95.6 | Mill Creek, Evendale | Hamilton | OH |  |
| **TMM 30967-1650** | Right | m2 | 113 | 91 | Ingleside | San Patricio | TX | Lundelius 1972 |
| **TMM 30967-50** | Left | m2 | 98 | 87 | Ingleside | San Patricio | TX | Lundelius 1972 |
| **BYUVP 4379** | Right | m2 | 101 | 76 | Sanpete Co. | Sanpete | UT | Miller 1987 |
| **VMNH 2320** | Left | m2 | 108 | 69.6 | Saltville | Smyth | VA |  |
| **ETMNH 19306** | Left | m2 | 83.5 | 74 | Saltville | Smyth | VA | Silverstein 2017 |
| **UWBM 88099** | Left | m2 | 104.45 | 74.27 | Winlock area C0856 | Lewis | WA |  |
| **UWBM 14491** | Right | m2 | 109.18 | 73.87 | Port Angeles | Clallum | WA |  |
| **YG 361.31** |  | m2 | 92.99 | 74.33 |  |  | Yukon |  |
| **LACM 117689** | Left | m2 | 105.25 | 72.91 | Minaca Mesa | Chihuahua | Mexico |  |
| **NMC 8147** |  | M1 | 83.4 | 74.9 | Bonanza Creek |  | AK | Harington, 1977 |
| **NMC 26601** |  | M1 | 81.2 | 61.3 | Old Crow Loc 11a |  | AK |  |
| **DMNH 69333** | Right | M1 | 96.4 | 78.4 | Zeigler Reservoir | Pitkin | CO |  |
| **DMNH 70776** | Right | M1 | 98.4 | 76.5 | Zeigler Reservoir | Pitkin | CO |  |
| **DMNH 70770** | Right | M1 | 94 | 77.7 | Zeigler Reservoir | Pitkin | CO |  |
| **DMNH 70761** | Left | M1 | 110.2 | 90.2 | Zeigler Reservoir | Pitkin | CO |  |
| **DMNH 70766** | Left | M1 | 109.4 | 86.4 | Zeigler Reservoir | Pitkin | CO |  |
| **DMNH 70762** | Right | M1 | 112.8 | 90.1 | Zeigler Reservoir | Pitkin | CO |  |
| **DMNH 72110** | Left | M1 | 96.1 | 78.1 | Zeigler Reservoir | Pitkin | CO |  |
| **DMNH 69327** | Left | M1 | 95 | 81.3 | Zeigler Reservoir | Pitkin | CO |  |
| **DMNH 69335** | Left | M1 | 94.6 | 80.4 | Zeigler Reservoir | Pitkin | CO |  |
| **UF 1013** | Left | M1 | 92.3 | 68.3 | Hornsby Springs | Alachua | FL | Green and Hulbert 2005 |
| **UF 118543** | Left | M1 | 81.3 | 68.1 | Ichetucknee River | Columbia | FL | Green and Hulbert 2005 |
| **UF 200677** | Left | M1 | 86.9 | 70.4 | Aucilla River, Sloth Hole | Taylor | FL | Green and Hulbert 2005 |
| **UF 200659** | Right | M1 | 90.5 | 75.3 | Aucilla River, Sloth Hole | Taylor | FL | Green and Hulbert 2005 |
| **CPI RM 1** | Left | M1 | 83.5 | 69.8 | Wacissa River | Jefferson | FL | Green and Hulbert 2005 |
| **CPI HM 5** | Left | M1 | 77.9 | 65.5 | Aucilla River | Taylor | FL | Green and Hulbert 2005 |
| **CPI HM 7** | Right | M1 | 85.4 | 75.5 | Suwanee River (Boys Ranch) | Hamilton | FL | Green and Hulbert 2005 |
| **CPI BM 12** | Right | M1 | 95.4 | 81.3 | Aucilla River (Half Mile Rise) | Taylor | FL | Green and Hulbert 2005 |
| **CPI BM 9** | Right | M1 | 101 | 75 | Aucilla River (Half Mile Rise) | Taylor | FL | Green and Hulbert 2005 |
| **G25650** | Left | M1 | 89 | 75.8 | Carter's Bog | Darke | OH |  |
| **F910** | Right | M1 | 104.53 | 76.67 | UO2311, Ward’s Creek, Grants Pass Quad | Jackson | OR |  |
| **ETMNH 19330** | Right | M1 | 92 | 78 | Saltville | Smyth | VA | Silverstein 2017 |
| **ETMNH 19328** | Right | M1 | 92.5 | 69 | Saltville | Smyth | VA | Silverstein 2017 |
| **DMNH 69921** | Left | m1 | 90.5 | 71.1 | Zeigler Reservoir | Pitkin | CO |  |
| **DMNH 70768** | Left | m1 | 92.5 | 68.5 | Zeigler Reservoir | Pitkin | CO |  |
| **DMNH 70784** | Left | m1 | 92.1 | 71.1 | Zeigler Reservoir | Pitkin | CO |  |
| **DMNH 70775** | Right | m1 | 89.4 | 68.3 | Zeigler Reservoir | Pitkin | CO |  |
| **DMNH 70781** | Left | m1 | 94.2 | 68.2 | Zeigler Reservoir | Pitkin | CO |  |
| **DMNH 69924** | Right | m1 | 86 | 65.6 | Zeigler Reservoir | Pitkin | CO |  |
| **UF 52919** | Right | m1 | 85.6 | 68.5 | Kennedy-La Belle | Hendry | FL | Green and Hulbert 2005 |
| **UF 135708** | Left | m1 | 86 | 62.1 | Aucilla River | Jefferson/Taylor | FL | Green and Hulbert 2005 |
| **DL 950711** | Right | m1 | 84.7 | 62.1 | Withlacoochee River | Marion | FL | Green and Hulbert 2005 |
| **UF 135707** | Right | m1 | 86.3 | 61.3 | Aucilla River | Jefferson/Taylor | FL | Green and Hulbert 2005 |
| **UF 135702** | Right | m1 | 87.6 | 65.8 | Aucilla River | Jefferson/Taylor | FL | Green and Hulbert 2005 |
| **UF 135710** | Right | m1 | 90.6 | 68.9 | Aucilla River | Taylor | FL | Green and Hulbert 2005 |
| **71.3.39.2** | Right | m1 | 98.5 | 74.5 | Dollens | Madison | IN | Richards et al. 1987 |
| **G25650** | Right | m1 | 87.8 | 67.6 | Carter's Bog | Darke | OH |  |
| **VMNH unnumbered** | Left | m1 | 96.2 | 71.9 | Saltville | Smyth | VA |  |
| **ETMNH 19329** | Right | m1 | 88 | 64 | Saltville | Smyth | VA | Silverstein 2017 |
| **ETMNH 19317** | Right | m1 | 93 | 73 | Saltville | Smyth | VA | Silverstein 2017 |
| **ETMNH 19327** | Left | m1 | 86 | 73 | Saltville | Smyth | VA | Silverstein 2017 |
| **UWBM 14491** | Right | m1 | 76.78 | 60.6 | Port Angeles | Clallam | WA |  |
| **DMNH 70756** | Left | dP4 | 81.4 | 65.2 | Zeigler Reservoir | Pitkin | CO |  |
| **DMNH 70783** | Left | dP4 | 71.4 | 59.4 | Zeigler Reservoir | Pitkin | CO |  |
| **DMNH 72110** | Left | dP4 | 73.5 | 63.1 | Zeigler Reservoir | Pitkin | CO |  |
| **UF 1862** | Left | dP4 | 71 | 59.8 | Hornsby Springs | Alachua | FL | Green and Hulbert 2005 |
| **UF 988** | Right | dP4 | 71.9 | 58.1 | Hornsby Springs | Alachua | FL | Green and Hulbert 2005 |
| **UF 918** | Right | dP4 | 72.9 | 59.7 | Hornsby Springs | Alachua | FL | Green and Hulbert 2005 |
| **UF 991** | Right | dP4 | 69.6 | 58.3 | Hornsby Springs | Alachua | FL | Green and Hulbert 2005 |
| **CPI BM 9** | Right | dP4 | 76.5 | 62 | Aucilla River (Half Mile Rise) | Taylor | FL | Green and Hulbert 2005 |
| **CPI BM 10** | Left | dP4 | 74.1 | 64.5 | Aucilla River (Half Mile Rise) | Taylor | FL | Green and Hulbert 2005 |
| **CPI BM 11** | Left | dP4 | 73.3 | 63.2 | Aucilla River (Half Mile Rise) | Taylor | FL | Green and Hulbert 2005 |
| **UF 212609** | Right | dP4 | 72.7 | 60.6 | Wacassa River | Levy | FL | Green and Hulbert 2005 |
| **UF 205747** | Left | dP4 | 74.7 | 62.6 | Wekiwa River 1 | Seminole | FL | Green and Hulbert 2005 |
| **G-unknown** | Right | dP4 | 73.6 | 62.5 | Carters Bog | Darke | OH |  |
| **UALP 9330f** | Right | dp4 | 75 | 56 | Lehner site | Cochise | AZ | Mead et al 1979 |
| **DMNH 70785** | Left | dp4 | 74.3 | 53.4 | Zeigler Reservoir | Pitkin | CO |  |
| **DMNH 70757** | Left | dp4 | 81.6 | 59.3 | Zeigler Reservoir | Pitkin | CO |  |
| **DMNH 70765** | Right | dp4 | 82 | 57.4 | Zeigler Reservoir | Pitkin | CO |  |
| **DMNH 70764** | Left | dp4 | 77.4 | 54.3 | Zeigler Reservoir | Pitkin | CO |  |
| **DMNH 69924** | Right | dp4 | 63.1 | 60.2 | Zeigler Reservoir | Pitkin | CO |  |
| **UF 135708** | Left | dp4 | 62.4 | 49.1 | Aucilla River | Jefferson/Taylor | FL | Green and Hulbert 2005 |
| **UF 131987** | Left | dp4 | 73.5 | 54 | Oklawaha River | Marion | FL | Green and Hulbert 2005 |
| **UF 137929** | Left | dp4 | 76 | 53.2 | Silver Springs | Marion | FL | Green and Hulbert 2005 |
| **UF 51193** | Left | dp4 | 79 | 52.2 | Palm Beach Farm | Palm Beach | FL | Green and Hulbert 2005 |
| **UF 135706** | Left | dp4 | 74.6 | 56.8 | Aucilla River | Taylor | FL | Green and Hulbert 2005 |
| **CMC VP 11731** | Right | dp4 | 89.6 | 69.7 | Gum Branch? | Boone? | KY |  |
| **G25693** | Right | dp4 | 74.17 | 65.27 | Carters Bog | Darke | OH |  |
| **ETMNH 19342** | Right | dp4 | 71 | 52 | Saltville | Smyth | VA | Silverstein 2017 |
| **UALP 9330i** | Right | dP3 | 47 | 45 | Lehner site | Cochise | AZ | Mead et al 1979 |
| **DMNH 70756** | Left | dP3 | 48.4 | 46.8 | Zeigler Reservoir | Pitkin | CO |  |
| **DMNH 70793** | Left | dP3 | 41.6 | 43.6 | Zeigler Reservoir | Pitkin | CO |  |
| **DMNH 70767** | Left | dP3 | 48.1 | 46.3 | Zeigler Reservoir | Pitkin | CO |  |
| **UF 160000** | Right | dP3 | 41.1 | 39.4 | Surprise Cave | Alachua | FL | Green and Hulbert 2005 |
| **UF 1863** | Right | dP3 | 46.5 | 41.8 | Hornsby Springs | Alachua | FL | Green and Hulbert 2005 |
| **UF 212310** | Left | dP3 | 45.2 | 47 | Aucilla River (Half Mile Rise) | Taylor | FL | Green and Hulbert 2005 |
| **UF 135744** | Right | dP3 | 45.2 | 46.2 | Aucilla River | Taylor | FL | Green and Hulbert 2005 |
| **UF 212584** | Right | dP3 | 41.1 | 40.4 | Withlacoochee River | Marion | FL | Green and Hulbert 2005 |
| **UF 212641** | Right | dP3 | 41 | 38.6 | Withlacoochee River | Marion | FL | Green and Hulbert 2005 |
| **UF 212643** | Left | dP3 | 39.3 | 41.3 | Withlacoochee River | Marion | FL | Green and Hulbert 2005 |
| **UF 212618** | Left | dP3 | 40.4 | 40.4 | Santa Fe River | Gilchrist | FL | Green and Hulbert 2005 |
| **UF 212640** | Right | dP3 | 43.4 | 42.7 | Withlacoochee River | Marion | FL | Green and Hulbert 2005 |
| **UF 212644** | Left | dP3 | 44 | 39.4 |  | Sarasota | FL | Green and Hulbert 2005 |
| **UF 212615** | Right | dP3 | 43.6 | 43.8 | Santa Fe River | Gilchrist | FL | Green and Hulbert 2005 |
| **UF 212614** | Right | dP3 | 41.2 | 42.1 | Santa Fe River | Gilchrist | FL | Green and Hulbert 2005 |
| **UF 212616** | Right | dP3 | 45.1 | 45.4 | Santa Fe River | Gilchrist | FL | Green and Hulbert 2005 |
| **LSUMG V-14526** |  | dP3 | 48 | 47 | Kimball Creek | West Feliciana | LA |  |
| **G25693** | Left | dP3 | 45.7 | 47.5 | Carters Bog | Darke | OH |  |
| **F102** |  | dP3 | 40.56 | 35.35 | UO12984 | Jackson | OR |  |
| **VMNH 120136** | Left | dP3 | 48.1 | 50 | Saltville | Smyth | VA |  |
| **VMNH 51138** | Left | dP3 | 41.8 | 46.4 | Saltville | Smyth | VA |  |
| **VMNH 51172** | Right | dP3 | 41.4 | 42.3 | Saltville | Smyth | VA |  |
| **VMNH 51132** | Left | dP3 | 47.4 | 42.4 | Saltville | Smyth | VA |  |
| **ETMNH 19355** | Right | dP3 | 45.7 | 43.7 | Saltville | Smyth | VA | Silverstein 2017 |
| **UALP 9330e** | Right | dp3 | 46 | 42 | Lehner site | Cochise | AZ | Mead et al 1979 |
| **DMNH 70794** | Left | dp3 | 47.3 | 43.9 | Zeigler Reservoir | Pitkin | CO |  |
| **DMNH 70757** | Left | dp3 | 50.2 | 45.5 | Zeigler Reservoir | Pitkin | CO |  |
| **UF 92650** | Right | dp3 | 41.2 | 39.1 | Aucilla 2 | Taylor | FL | Green and Hulbert 2005 |
| **UF 135706** | Left | dp3 | 45.5 | 38.6 | Aucilla River | Taylor | FL | Green and Hulbert 2005 |
| **UF 160000** | Right | dp3 | 43.6 | 38 | Surprise Cave | Alachua | FL | Green and Hulbert 2005 |
| **G25693** | Left | dp3 | 48.55 | 45.03 | Carters Bog | Darke | OH |  |
| **ETMNH 19324** | Left | dp3 | 47.5 | 52 | Saltville | Smyth | VA | Silverstein 2017 |
| **UF 135747** | Right | dP2 | 34.7 | 34.7 | Aucilla River | Taylor | FL | Green and Hulbert 2005 |
| **UF 160000** | Right | dP2 | 33.1 | 30.6 | Surprise Cave | Alachua | FL | Green and Hulbert 2005 |
| **UF 212312** | Right | dP2 | 35.1 | 35.4 | Aucilla River | Taylor | FL | Green and Hulbert 2005 |
| **UF 212311** | Left | dP2 | 36.4 | 35.3 | Aucilla River | Taylor | FL | Green and Hulbert 2005 |
| **G-unknown** | Left | dP2 | 33.6 | 37.2 | Carters Bog | Darke | OH |  |
| **VMNH 51182** | Left | dP2 | 35 | 34.5 | Saltville | Smyth | VA |  |
| **UALP 9330d** | Right | dp2 | 35 | 31 | Lehner site | Cochise | AZ | Mead et al 1979 |
| **DMNH 70792** | Right | dp2 | 33.7 | 29.5 | Zeigler Reservoir | Pitkin | CO |  |
| **DMNH 70795** | Right | dp2 | 35.7 | 27.6 | Zeigler Reservoir | Pitkin | CO |  |
| **DMNH 70796** | Left | dp2 | 35.2 | 31.3 | Zeigler Reservoir | Pitkin | CO |  |
| **MCZ 17815** | Left | dp2 | 31.3 | 25 | Melbourne | Brevard | FL | Green and Hulbert 2005 |
| **UF 160000** | Right | dp2 | 31.5 | 27.5 | Surprise Cave | Alachua | FL | Green and Hulbert 2005 |
| **G25693** | Right | dp2 | 32.2 | 33.13 | Carters Bog | Darke | OH |  |
| **Irvingtonian teeth of *M. americanum* included in this study** | | | | | | | | |
| **UF 215058** |  | M3 | 162 | 86.7 | La Belle Hwy | Hendry | FL | Green 2006 |
| **UF 206855** |  | M3 | 159.7 | 83.4 | Tri-Britton | Hendry | FL | Green 2006 |
| **GO 6** |  | M3 | 151.1 | 93.5 | Leisey Shell Pit | Hillsborough | FL | Green 2006 |
| **BF 19** |  | M3 | 176 | 104.5 | Leisey Shell Pit | Hillsborough | FL | Green 2006 |
| **UF 215057** |  | m3 | 162 | 86.7 | La Belle Hwy | Hendry | FL | Green 2006 |
| **UF 215061** |  | m3 | 181.7 | 91.8 | La Belle Hwy | Hendry | FL | Green 2006 |
| **UF 215059** |  | m3 | 184 | 96 | La Belle Hwy | Hendry | FL | Green 2006 |
| **UF 206851** |  | m3 | 164.1 | 84 | Tri-Bretton Site | Hendry | FL | Green 2006 |
